# Cell Cycle Control of Nuclear Metabolism Couples Phosphatidylinositol Signaling to Histone Methylation

**DOI:** 10.1002/advs.202501083

**Published:** 2026-04-21

**Authors:** Antoni Gañez‐Zapater, Savvas Kourtis, Camilla Reiter Elbæk, Lorena Espinar, Carolina Toro‐Márquez, Albert Coll‐Manzano, Alfredo Smiriglia, Laura García‐López, Laura Wiegand, Maria Guirola, Frédéric Fontaine, Andrea Morandi, André C. Müller, Sara Sdelci

**Affiliations:** ^1^ Centre For Genomic Regulation (CRG) The Barcelona Institute of Science and Technology Barcelona Spain; ^2^ Department of Experimental and Clinical Biomedical Sciences University of Florence Florence Italy; ^3^ (Now) Charité – Universitätsmedizin Berlin Corporate Member of Freie Universität Berlin and Humboldt‐Universität zu Berlin, and Berlin Institute of Health Department of Hematology, Oncology, and Cancer Immunology Berlin Germany; ^4^ CeMM Research Center for Molecular Medicine of the Austrian Academy of Sciences Vienna Austria; ^5^ Universitat Pompeu Fabra (UPF) Barcelona Spain

**Keywords:** cell cycle, chromatin, epigenetics, nuclear metabolism, proteomics

## Abstract

Progression through the cell cycle requires coordinated regulation of transcription, chromatin state, and cellular metabolism. While metabolic enzymes are known to localize the nucleus and influence chromatin states, how nuclear metabolism itself oscillates during the cell cycle remains unexplored. Here, we combine a customized FUCCI‐3 reporter with chromatome mass spectrometry and high‐throughput imaging to systematically resolve nuclear and chromatin‐associated metabolic changes across cell cycle phases. We identify phosphatidylinositol metabolism as a nuclear pathway that oscillates with the cell cycle, with PIP5K1A, PLCD3, and PLD2 showing phase‐specific nuclear and chromatin dynamics. Nuclear PIP2 levels redistribute within the nucleus depending on cell cycle stage. Downregulation of PIP5K1A reduces nuclear PIP2 levels, whereas nuclear enrichment of PIP5K1A increases PIP2 abundance in the nucleus and nucleolus, functionally linking PIP5K1A nuclear localization to nuclear PIP2 synthesis. Moreover, perturbation of nuclear PIP2 synthesis alters chromatin methylation, with a pronounced impact on H4K20 monomethylation. Together, our results reveal that nuclear phosphatidylinositol metabolism is cell cycle regulated and functionally linked to chromatin methylation, establishing nuclear lipid metabolism as a previously unrecognized layer of cell cycle control.

## Graphical abstract

Nuclear metabolism oscillates during cell cycle progression. Quantitative chromatome proteomics and imaging reveal phase‐specific dynamics of PIP5K1A and nuclear PIP2, linking phosphatidylinositol metabolism to histone methylation. This work identifies nuclear lipid metabolism as a previously unrecognized regulatory axis coordinating chromatin organization with cell cycle progression.

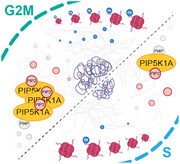

## 1 Introduction

The eukaryotic cell cycle is a highly regulated process that controls cell division and proliferation. Central to this regulatory network is the cell cycle‐coordinated control of chromatin, which determines major epigenetic modifications [1] that fine‐tune transcription [2], control DNA replication [3], and allow chromosome compaction and correct segregation [4]. Recent advances in live‐cell imaging technologies have provided unprecedented insights into the dynamics of cell cycle progression, revealing intricate spatial and temporal patterns of molecular activities [5]. One of such technologies is the Fluorescence Ubiquitination Cell Cycle Indicator (FUCCI) system that enables the visualization of cell cycle phases based on the expression levels of fluorescent gene reporters targeted to specific cell cycle regulators [6, 7]. The first generation of FUCCI only allowed discrimination of proliferating and non‐proliferating cells [8, 9]. By integrating the tracking of three different cell cycle markers [Chromatin Licensing And DNA Replication Factor 1 (Cdt1), stem‐loop binding protein (SLBP) and Geminin] and Histone 1, the more recent FUCCI‐4 system allows for real‐time monitoring of cell cycle transitions and chromatin condensation during mitosis, revealing insights into the heterogeneity and plasticity of cell cycle progression within populations of cells [6, 10].

Beyond its evident role in chromatin dynamics, emerging evidence suggests that the cell cycle is intricately linked to cellular metabolism [11, 12, 13]. Metabolic processes, including glycolysis, oxidative phosphorylation and lipid metabolism, provide the necessary energy and building blocks to support DNA duplication, cell growth, and proliferation [14, 15, 16, 17, 18, 19, 20, 21]. Interestingly, metabolic enzymes have been shown to exert regulatory functions beyond their canonical metabolic roles, influencing signaling pathways and epigenetic modifications that govern chromatin structure and function. Indeed, the nuclear localization of metabolic enzymes regulates chromatin states and fates, influencing histone posttranslational modification [22, 23, 24, 25] as well as facilitating the DNA damage repair [24, 26, 27], regulating transcription [28, 29], and controlling the faithful progression of cell cycle gene expression control [30] and mitosis [31].

Despite the complex relationship between metabolism and the cell cycle, and the evidence that metabolic enzymes can localize to the nucleus to regulate chromatin states and functions, there has been no attempt to elucidate how nuclear metabolism changes over the cell cycle, nor how these changes affect chromatin states. In this study, we employed a custom FUCCI reporter to couple cell cycle‐based sorting and the isolation of chromatin‐bound proteomes, with the objective of identifying metabolic enzymes that fluctuate in their presence on chromatin during the cell cycle. Our results demonstrate that phosphatidylinositol‐related enzymes oscillate on chromatin in a cell cycle‐dependent manner, regulating histone methylation.

## 2 Results

### 2.1 Optimization and Characterization of a FUCCI‐3 Stable Reporter

**FIGURE 1:**
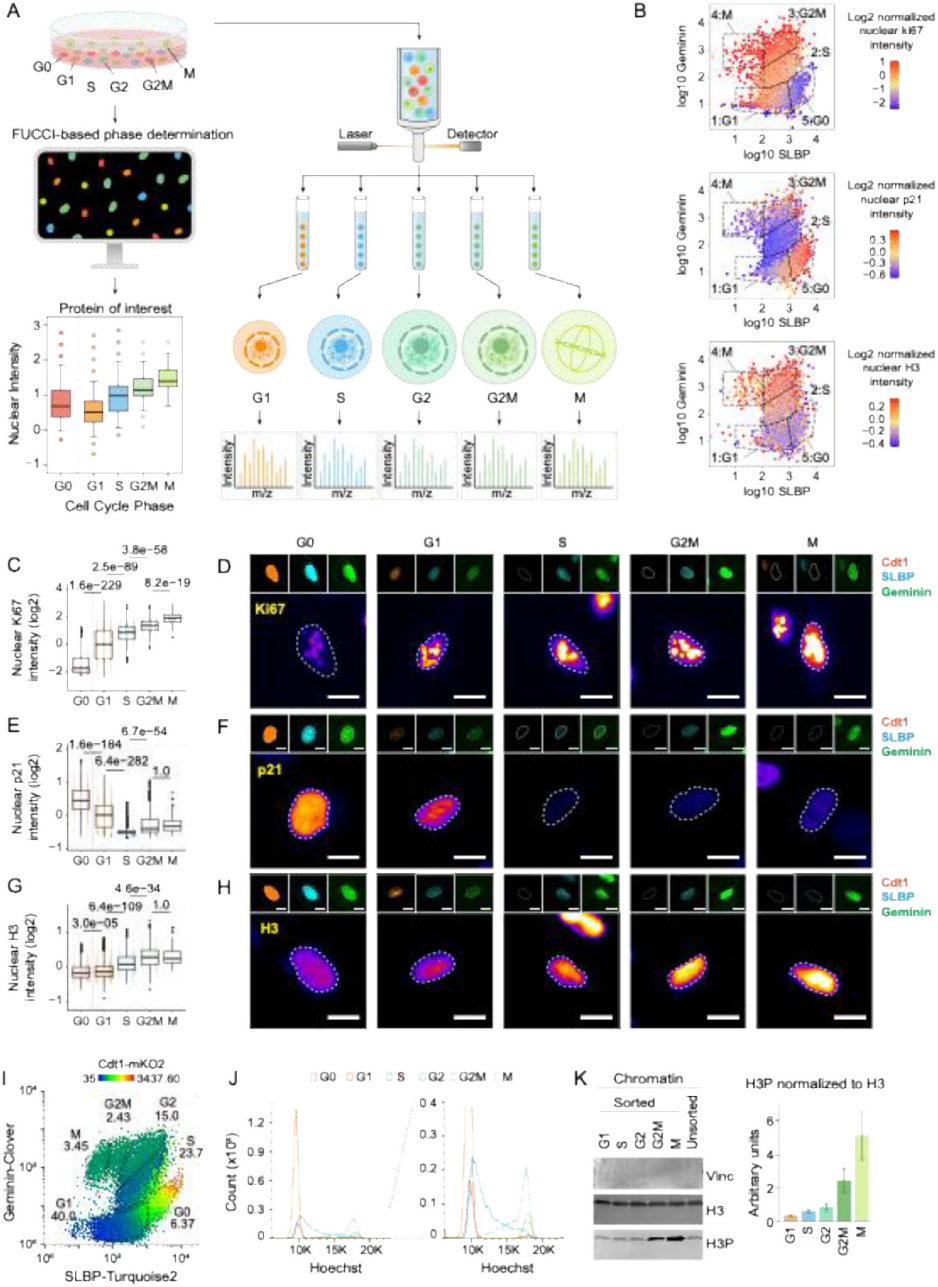
Characterization of U2OS‐FUCCI‐3 reporter system for the analysis of cell cycle‐dependent chromatin associations. (A) Experimental overview: U2OS‐FUCCI3 cells were cultured to maintain a stable distribution across different cell cycle phases. Subsequently, they were either subjected to microscopy for immunofluorescence or live‐cell imaging studies or sorted by FACS to obtain protein extracts for mass spectrometry analysis. (B) Representation of Ki67, p21, and H3 nuclear intensity measured by immunofluorescence and plotted accordingly to FUCCI‐3‐determined cell cycle phases. (C) Immunofluorescence‐based quantification of the intensity of Ki67 in the nuclear region identified as the combined signal from the FUCCI‐3 fluorescent markers, which has been used for the determination of cell cycle phases. 3 biological replicates were analyzed (*n*G0  =  1186, *n*G1  =  3071, *n*S  =  1129, *n*G2/M  =  530, *n*M  =  102; outliers removed, 3 SD; unpaired two‐tailed Wilcoxon test). (D) Representative images showing the changes of Ki67 inside the nucleus during cell cycle progression, as well as the respective signal of the FUCCI‐3 fluorescent markers of the illustrated cell, which confirm the belonging to the assigned cell cycle phase. Scale is 20 µm. (E) Immunofluorescence‐based quantification of the intensity of p21 in the nuclear region identified as the combined signal from the FUCCI‐3 fluorescent markers, which has been used for the determination of cell cycle phases. 3 biological replicates were analyzed (*n*G0  =  1382, *n*G1  =  2798, *n*S  =  1137, *n*G2/M  =  631, *n*M  =  128; outliers removed, 3 SD; unpaired two‐tailed Wilcoxon test). (F) Representative images showing the changes of p21 inside the nucleus during cell cycle progression, as well as the respective signal of the FUCCI‐3 fluorescent markers of the illustrated cell, which confirm the belonging to the assigned cell cycle phase. Scale is 20 µm. (G) Immunofluorescence‐based quantification of the intensity of H3 in the nuclear region identified as the combined signal from the FUCCI‐3 fluorescent markers, which has been used for the determination of cell cycle phases. 3 biological replicates were analyzed (*n*G0  =  1438, *n*G1  =  2867, *n*S  =  1176, *n*G2/M  =  644, *n*M  =  103; outliers removed, 3 SD; unpaired two‐tailed Wilcoxon test). (H) Representative images showing the changes of H3 inside the nucleus during cell cycle progression, as well as the respective signal of the FUCCI‐3 fluorescent markers of the illustrated cell, which confirm the belonging to the assigned cell cycle phase. Scale is 20 µm. (I) Comparison between FUCCI‐3 based identification of cell cycle phases and (J) FACS‐based cell cycle profiles obtained with Hoechst staining. (K) Western blot validation of the chromatin fractionation using Vinculin as cytoplasmic marker and H3 as chromatin marker accompanied by the quantification of Phosphorylated H3 (H3P, serine 10) normalized to H3 during cell cycle.

To characterize the chromatin binding proteomes at the different stages of the cell cycle, a stable U2OS reporter cell line was generated with a customized version of the FUCCI‐4 system [10] henceforth referred to as FUCCI‐3. The FUCCI‐3 system enabled the tracking of cells through the cell cycle phases using either high‐throughput immunofluorescence or fluorescence‐activated cell sorting (FACS), thereby facilitating the isolation of cell cycle‐specific populations and the quantification of selected markers in a cell cycle‐dependent manner (Figure 1A). In addition, since there was no need for cell cycle inhibitors to isolate or track different cell cycle populations, our system avoided the naturally introduced biases due to cell cycle arrest. As with the original FUCCI‐4, the FUCCI‐3 reporter system expresses fragments of Cdt1, SLBP, and Geminin, respectively tagged with mKO2, Turquoise2, and Clover. However, we eliminated the expression of Histone 1, tagged with the Maroon1 fluorescence marker, which proved to be deleterious to cell proliferation when stably expressed in U2OS cells following lentiviral transduction (Figure S1A). The integration of the expression levels of the Cdt1, SLBP, and Geminin fluorescent markers allowed the classification of cell cycle phases and phase transitions, as previously described [10]. Leveraging this, we developed a pipeline for the analysis of high‐throughput microscopy images in the context of the cell cycle (Figure S1B). In brief, images of the FUCCI‐3 fluorescent markers were acquired and merged, resulting in the identification of all nuclei, with the only exception of the cytokinetic nuclei. A perinuclear ring region around the nucleus was used to measure the background intensity of the FUCCI fluorescent markers. The background was then subtracted from the mean intensity of the nuclei, allowing for the determination of the corrected level of each of the FUCCI fluorescent markers inside the nucleus. This pipeline enabled a novel microscopy‐based analysis of cell cycle, thereby providing a valuable tool for the precise identification and characterization of cell cycle phases at the single nuclei resolution. Removing the Histone 1‐Maroon1 reporter allowed the imaging of proteins of interest. In addition, the use of high‐throughput microscopy instead of FACS, which is typically used for cell cycle studies, allowed information about cellular structures and subcellular localization of proteins to be preserved. To validate our U2OS FUCCI‐3 reporter cell line, we sought to identify cell cycle phases by integrating FUCCI‐3 fluorescent markers with nuclear quantification of three well‐known cell cycle‐associated proteins throughout cell cycle progression: Kiel antigen 67 (Ki67), which is highly expressed in proliferating cells [32], Cyclin‐dependent kinase inhibitor 1A (p21), whose expression promotes cell cycle arrest [33], and Histone h3 (H3), which is an integral part of nucleosomes and therefore reflects the amount of DNA present in a cell. U2OS FUCCI‐3 reporter cells were fixed and stained with antibodies that recognize these markers and analyzed using high‐throughput microscopy. The three stained populations (Ki67, p21, and H3) exhibited comparable cell cycle profiles, as illustrated by the FUCCI‐3 plots (Figure S1C). In these plots, the intensity of Geminin‐Clover is represented on the y‐axis, the intensity of the SLBP1‐Turquoise2 is represented on the x‐axis, and the intensity of Cdt1‐mKO2 is shown as a blue‐to‐red color gradient. The integration of the FUCCI‐3 reporters expression enabled the identification of five distinct cell populations. Population 1 was found to correspond to G1 cells, which typically express low levels of Geminin‐Clover, low levels of Cdt1‐mKO2, and medium levels of SLBP‐Turquoise2. Population 2 was identified as S phase cells, which are distinguished by an increase in Geminin‐Clover expression, reaching medium levels. Population 3 consisted of cells in G2 phase, which exhibited the highest expression of Geminin‐Clover. Population 4 consisted of cells undergoing the transition to mitosis, which was evidenced by the progressive loss of SLBP‐Turquoise2 expression. Furthermore, we identified a fifth population that was characterized by high levels of SLBP‐Turquoise2 and Cdt1‐mKO2 that had not been previously characterized with a FUCCI‐4‐derived system. The intensity of Ki67 in the nucleus increased with cell cycle progression. In contrast, p21 levels were significantly low in proliferating cells, reaching a minimum in S phase. In addition, the intensity of H3 was found to double from S phase onward, consistent with the DNA duplication process (Figure 1B). We observed that the fifth population, newly identified in this study, had the lowest Ki67 levels (Figure 1B–D), strong p21 expression (Figure 1B,E,F), and lower intensity of H3 like cells in G1 (Figure 1B,G,H). Based on these characteristics, we concluded that this population had left or was in the process of leaving the cell cycle and therefore designated it as G0.

Given that the choice of microscopy over FACS also enabled the analysis of nuclear characteristics, we incorporated the size of the nucleus into the evaluation of the expression of these cell cycle markers. As expected, cell cycle progression was accompanied by an increase in nuclear size (Figure S1D). The mean nuclear intensity reflects the concentration of the targeted protein; therefore, the total amount of protein can be estimated by multiplying the mean intensity by the nuclear area (i.e., the integrated intensity).

We observed that the behavior of Ki67 and p21 during the cell cycle was similar when considering the mean intensity (Figure 1C–F) or the integrated intensity (Figure S1E). In proliferating cells, H3 concentration and protein amounts also showed similar behavior. However, while the H3 concentration decreased in G0 cells compared to G1 cells (Figure 1G,H), the total amount of H3 protein was the same in G0 and G1 cells (Figure S1E). This effect can be explained by the enlarged nuclei that characterize the G0 population (Figure S1D) and rules out the possibility that G0 cells lost genetic material. Both G0 and senescent cells are characterized by exiting cell cycle, however in the case of the senescent this event is not reversible. To evaluate to which extent our G0 named population could contain senescent cells, we performed immunostaining for LaminB1, which concentration decreases in senescent cells [34], and p16, which typically increases in senescent cells [35]. The G0 population showed overall less LaminB1 and more p16 intensity compared to the G1 gate population (Figure S1F–J), suggesting that part of the cells in the G0 population may be senescent.

To validate our results, we generated a stable MCF7 FUCCI‐3 reporter cell line, which recapitulated the observations made in U2OS FUCCI‐3 cells in both proliferative (Figure S2A–I) and cell cycle arrested (Figure S2J–O) subpopulations, confirming the scalability of the system and the robustness of its interpretation.

Comparison of FUCCI‐3 fluorescence with Hoechst staining followed by FACS analysis showed that DNA content aligned with the FUCCI‐3‐defined cell cycle phases. The reporter also resolved G2, G2/M, and M phases, distinctions not achievable with Hoechst alone (Figure 1I,J). Unlike Hoechst, which intercalates into DNA, the FUCCI‐3 system does not impair cell viability over time, allowing long‐term tracking without perturbing the cell cycle. To test the suitability of the FUCCI‐3 reporter cell line for cell cycle tracking, we used drugs that arrest the cell cycle at specific phases. Cells were treated with either RO‐3306, a cyclin‐dependent kinase 1 (CDK1) inhibitor that arrests cells at the G2/M boundary [36], and the tubulin polymerization inhibitor nocodazole [37], which blocks cells in prometaphase. By performing live cell imaging, we observed that cell proliferation was affected by both treatments (Figure S3A). Moreover, tracking the different reporter expressions allowed us to identify the cell cycle arrest at the expected phases (Figure S3B,C), confirming the suitability of the generated reporter for live cell imaging studies. Thus, live‐cell imaging–based cell cycle tracking over 64 h was used to confirm the nature of the G0 population. During this period, most cells starting in G1 completed a full cell cycle within 32 h, whereas G0 cells showed a marked delay. A subset of G0‐gated cells never re‐entered the cycle, indicating that the G0 fraction comprises both quiescent cells capable of cell cycle reactivation and senescent cells that no longer proliferate (Figure S3D,E). Finally, we assessed the suitability of the FUCCI‐3 system for viable FACS sorting. Five populations corresponding to proliferative cell cycle phases and transitions (G1, S, G2, G2/M, and M) were isolated based on FUCCI‐3 fluorescence (Figure S3F). Sorted cells were re‐plated and monitored by live‐cell imaging for 40 h, confirming viability and expected cell cycle progression (Figure S3G). Each population was then subjected to an in‐house–optimized chromatin purification workflow, including sonication and benzonase digestion, to isolate chromatin‐associated proteins [26, 27, 31] (Figure S3H). For the M‐phase population, which lacks a nuclear membrane, the protocol was adapted by omitting the nuclear lysis step; instead, chromatin was recovered after cell‐membrane lysis, washing, and sonication before digestion. Sample purity and correct phase assignment were validated by Western blot using Vinculin (cytoplasmic marker), H3 (chromatin marker), and phospho‐H3 Ser10 (G2–M marker) (Figure 1K; Figure S3I).

In summary, these results showed that the optimized FUCCI‐3 reporter system allowed the generation of stable cell lines expressing cell cycle reporters for FACS and imaging‐based analysis that can be used for phenotypic and molecular dissection of cell cycle‐related events.

### 2.2 Cell‐Cycle–Resolved Chromatome Profiling Reveals Oscillatory Chromatin Association of Enzymes

To identify cell cycle–dependent changes in the chromatome, we performed data‐independent acquisition mass spectrometry (DIA‐MS) on FUCCI‐3‐sorted populations. PCA of normalized data showed clear clustering of biological replicates and strong separation between the sorted phases and the unsorted, asynchronous cells (Figure S4A,B). Chromatin purity was confirmed through relative compartment enrichment analysis (Figure S4C). Proteins displaying oscillatory behavior across the cell cycle were then clustered based on significant changes between at least two consecutive phases. These clusters highlighted distinct temporal profiles, such as proteins progressively increasing from G1 to M (cluster 1), enriched for cell cycle regulation, and proteins peaking toward mitosis (cluster 10), enriched for chromosome segregation (Figure S4D–F). Together, these analyses validated the robustness of our workflow for high‐throughput characterization of chromatin‐bound proteins during cell cycle progression.

**FIGURE 2:**
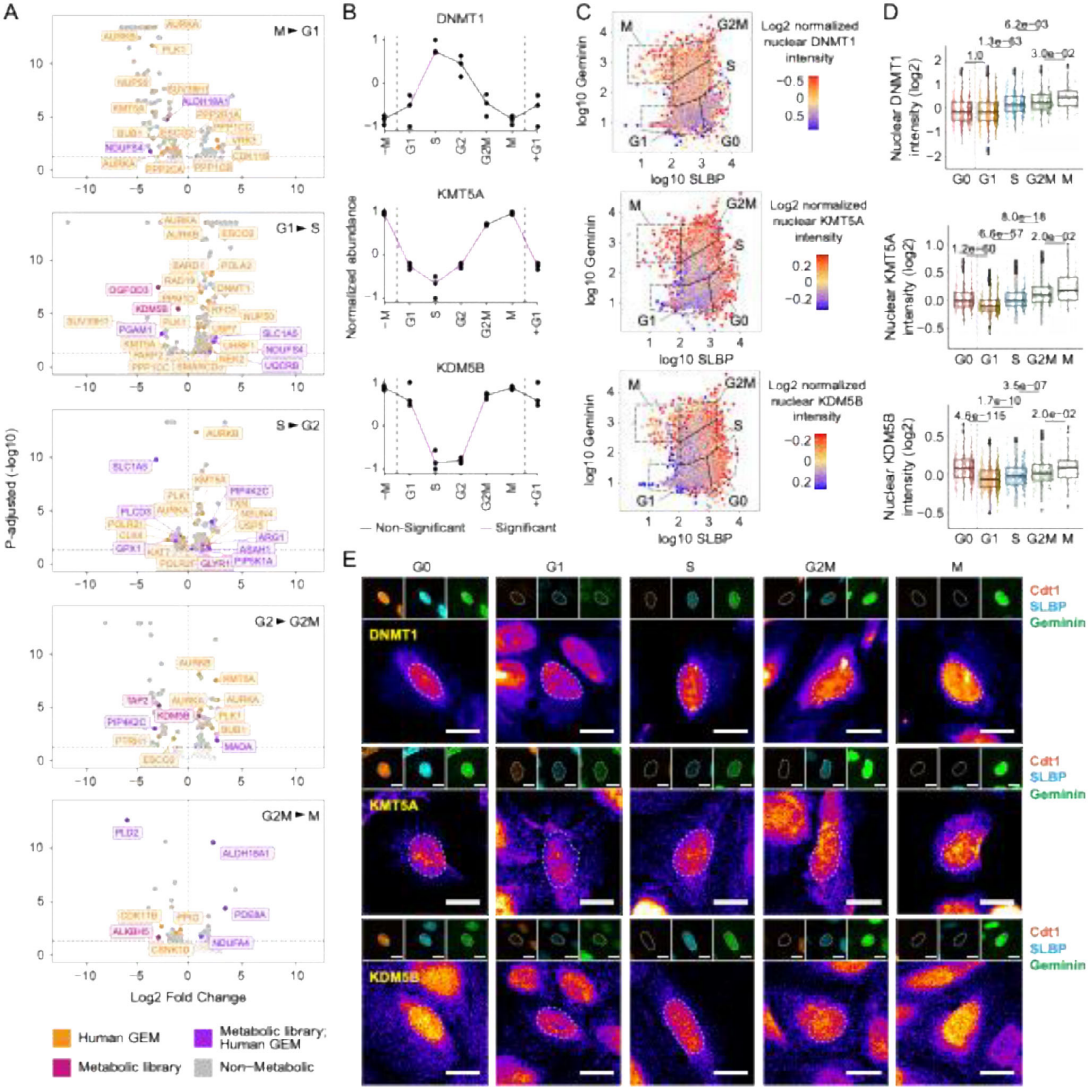
Cell cycle control of chromatin and nuclear localization of methylation‐related enzymes. (A) Volcano plots of mass spectrometry data showing the chromatin enrichment and depletion of proteins related to metabolism. Proteins belonging to the human genome‐scale metabolic model (GEM) [38] are represented in orange, proteins belonging to the metabolic sgRNA library [39] are represented in pink, proteins belonging to both groups are represented in violet. In gray there are proteins not related to metabolism. (B) Relative abundance of DNMT1, KMT5A and KDM5B on chromatin across cell cycle as determined by mass spectrometry on chromatin fractions. Magenta lines mean that the difference between the 2 consecutive phases was significant. Black lines mean that no significant difference was found between consecutive phases. Statistical analysis was performed according to the test_diff function in DEP R package [97], following intensity imputation, which internally uses limma statistical testing [109]. (C) Representation of DNMT1, KMT5A or KDM5B nuclear intensity measured by immunofluorescence and plotted according to FUCCI‐3‐determined cell cycle phases. (D) Immunofluorescence‐based quantification of nuclear DNMT1, KMT5A or KDM5B intensities across cell cycle phases determined with the FUCCI‐3 system. 3 biological replicates were analyzed (DNMT1 IF: *n*G0  =  1302, *n*G1  =  2793, *n*S  =  1073, *n*G2/M  =  543, *n*M  =  96; KMT5A IF: *n*G0  =  1183, *n*G1  =  2843, *n*S  =  939, *n*G2/M  =  564, *n*M  =  103; KDM5B IF: *n*G0  =  1258, *n*G1  =  3433, *n*S  =  1196, *n*G2/M  =  583, *n*M  =  117; outliers removed, 3 SD; unpaired two‐tailed Wilcoxon test). (E) Representative images showing the changes of DNMT1, KMT5A or KDM5B inside the nucleus during cell cycle progression, as well as the respective signal of the FUCCI‐3 fluorescent markers of the illustrated cell, which confirm the belonging to the assigned cell cycle phase. Scale is 20 µm.

To investigate whether nuclear metabolism is coordinated with the cell cycle, we next screened our chromatome dataset for metabolic genes and regulators, defined as members of the generic genome‐scale metabolic model of Homo sapiens (human GEM) [38] or targets of the human CRISPR‐Cas9 metabolic library [39] (Figure 2A; Figure S5A). Several metabolism‐related factors showed dynamic chromatin associations, including both metabolic regulators and canonical metabolic enzymes. Among the metabolic regulators, we identified Aurora kinases (AURKA and AURKB), which use ATP as phosphate donors [40, 41] and are linked to metabolic processes through interactions with ATP synthases [42] and phosphorylation of Pyruvate Kinase M2 (PKM2) [43]. Their chromatin association was minimal in G1 and gradually increased toward G2/M (Figure S5A, Cluster 3).

Epigenetic enzymes influencing methyl‐group balance, including DNMT1, KMT5A, SUV39H1, and KDM5B, also exhibited dynamic chromatin association during the cell cycle (Figure 2B; Figure S5A). SUV39H1 increased steadily (Cluster 3), similar to Aurora kinases. In contrast, DNMT1, KMT5A, and KDM5B showed phase‐specific patterns (DNMT1 in Cluster 2; KMT5A and KDM5B in Cluster 4), with DNMT1 rising from G1 to S before decreasing toward G2/M, and KMT5A/KDM5B showing the opposite trend.

To determine whether these chromatin fluctuations reflected differential chromatin binding or changes in overall nuclear abundance, we quantified nuclear intensities of DNMT1, KMT5A, and KDM5B by high‐throughput immunofluorescence in fixed U2OS FUCCI‐3 cells (Figure S5B–D). DNMT1 concentration and total amounts increased from G1 to S and then plateaued, while KMT5A and KDM5B increased from G1 through S and then again toward G2/M (Figure 2C–E; Figure S5E–G). Integrating DIA‐MS and IF data revealed that DNMT1's decrease on chromatin during G2/M occurred despite stable nuclear abundance, consistent with chromatin eviction during chromosome condensation (Figure 2B–E). For KMT5A and KDM5B, the chromatin association in S did not reflect nuclear abundance, although both increased in G2 (Figure 2B–E). These trends were reproduced in MCF7 FUCCI‐3 cells (Figure S6A–L).

Using this workflow, which distinguishes nuclear from chromatin‐bound proteins across the cell cycle, we uncovered the dynamic behavior of epigenetic enzymes involved in DNA and histone methylation. Our results further suggest that changes in nuclear size during the cell cycle influence nuclear enzyme concentration and thereby regulate their availability on chromatin.

### 2.3 Cell‐Cycle–Dependent Chromatin Association of Phosphatidylinositol‐Metabolic Enzymes

**FIGURE 3:**
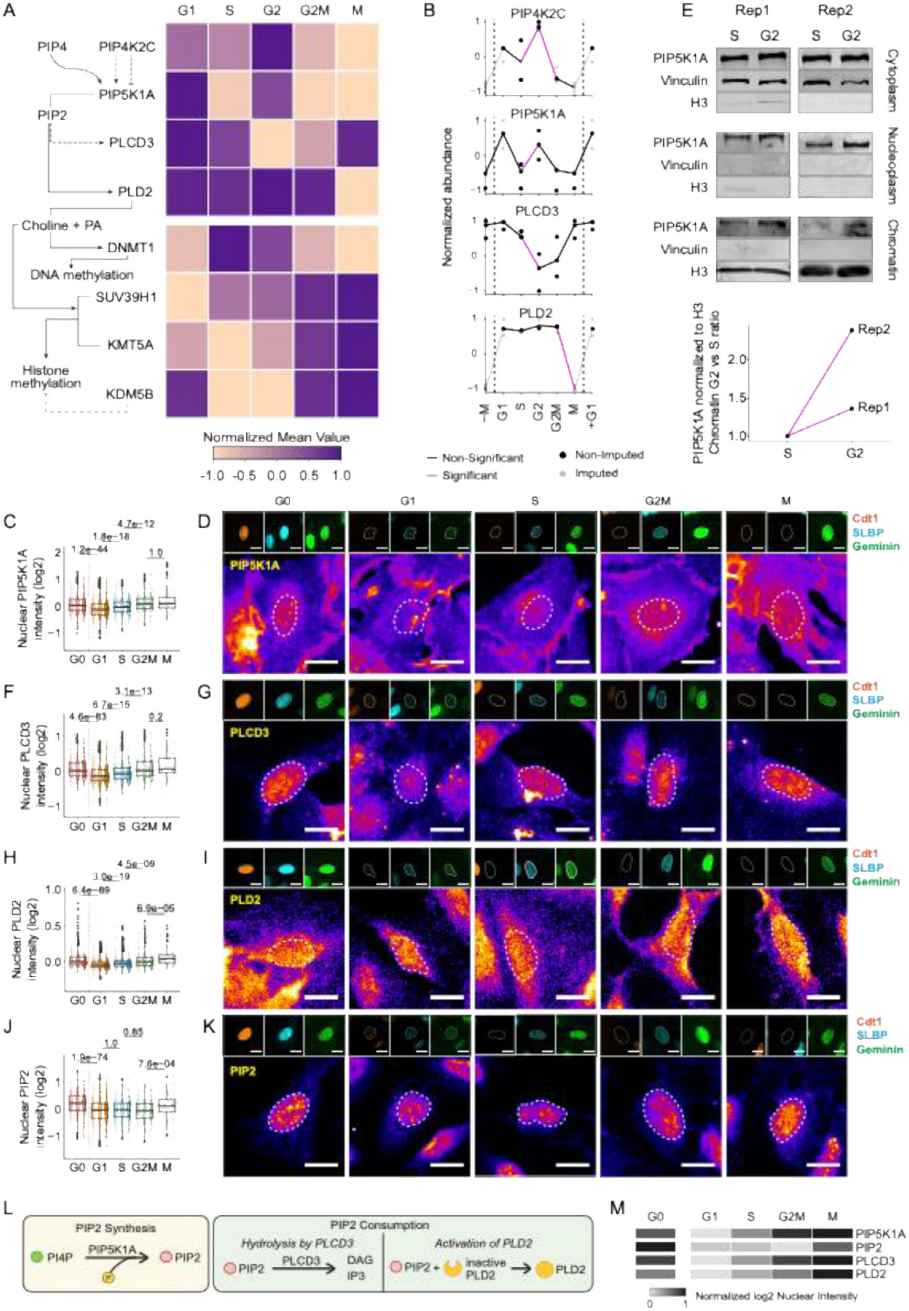
Phosphatidylinositol metabolism oscillates on chromatin during the cell cycle. (A) Reconstruction of a chromatin‐associated metabolic network representing the integration of phosphatidylinositol metabolism and chromatin methylation, obtained by plotting the normalized mean value of the abundance of each protein determined by mass spectrometry in each cell cycle phase. (B) Relative chromatin abundance of PIP5K1A, PLCD3 and PLD2 across cell cycle as determined by mass spectrometry on chromatin fractions. Magenta lines mean that the difference between the 2 consecutive phases was significant. Black lines mean that no significant difference was found between consecutive phases. Semi‐transparent dots correspond to imputed values. Statistical analysis was performed according to the test_diff function in DEP R package [97], following intensity imputation, which internally uses limma statistical testing [109]. (C) Immunofluorescence‐based quantification of nuclear PIP5K1A intensities across cell cycle phases determined with the FUCCI‐3 system. 3 biological replicates were analyzed (*n*G0  =  1261, *n*G1  =  3124, *n*S  =  1124, *n*G2/M  =  549, *n*M  =  123; outliers removed, 3 SD; unpaired two‐tailed Wilcoxon test). (D) Representative images showing the changes of PIP5K1A inside the nucleus during cell cycle progression, as well as the respective signal of the FUCCI‐3 fluorescent markers of the illustrated cell, which confirm the belonging to the assigned cell cycle phase. Scale is 20 µm. (E) Western blot analysis of PIP5K1A levels in chromatin, nucleoplasm and cytoplasm fractions in S and G2 phases in 2 biological replicates. H3 and Vinculin are used as chromatin and cytoplasmic controls, respectively. The S/G2 ratio of PIP5K1A was quantified using H3 as a loading control. Bottom panel show the relative PIP5K1A quantification. (F) Immunofluorescence‐based quantification of nuclear PLCD3 intensities across cell cycle phases determined with the FUCCI‐3 system. 3 biological replicates were analyzed (*n*G0  =  1353, *n*G1  =  3079, *n*S  =  1142, *n*G2/M  =  561, *n*M  =  106; outliers removed, 3 SD; unpaired two‐tailed Wilcoxon test). (G) Representative images showing the changes of PLCD3 inside the nucleus during cell cycle progression, as well as the respective signal of the FUCCI‐3 fluorescent markers of the illustrated cell, which confirm the belonging to the assigned cell cycle phase. Scale is 20 µm. (H) Immunofluorescence‐based quantification of nuclear PLD2 intensities across cell cycle phases determined with the FUCCI‐3 system. 3 biological replicates were analyzed (*n*G0  =  1180, *n*G1  =  3283, *n*S  =  1261, *n*G2/M  =  570, *n*M  =  128; outliers removed, 3 SD; unpaired two‐tailed Wilcoxon test). (I) Representative images showing the changes of PLD2 inside the nucleus during cell cycle progression, as well as the respective signal of the FUCCI‐3 fluorescent markers of the illustrated cell, which confirm the belonging to the assigned cell cycle phase. Scale is 20 µm. (J) Immunofluorescence‐based quantification of nuclear PIP2 intensities across cell cycle phases determined with the FUCCI‐3 system. 3 biological replicates were analyzed (*n*G0  =  1424, *n*G1  =  2970, *n*S  =  1127, *n*G2/M  =  643, *n*M  =  102; outliers removed, 3 SD; unpaired two‐tailed Wilcoxon test). (K) Representative images showing the changes of PIP2 inside the nucleus during cell cycle progression, as well as the respective signal of the FUCCI‐3 fluorescent markers of the illustrated cell, which confirm the belonging to the assigned cell cycle phase. Scale is 20 µm. (L) Schematic representation of PIP2 synthesis via PIP5K1A‐mediated phosphorylation of phosphatidylinositol‐4‐phosphate (PI4P), and PIP2 consumption through PLCD3‐mediated hydrolysis generating diacylglycerol (DAG) and inositol‐1,4,5‐trisphosphate (IP3), as well as activation of PLD2. (M) Heatmap representing phosphatidylinositol metabolism in the nucleus during cell cycle obtained by plotting the normalized mean intensity in log2 scale.

The metabolism of phosphatidylinositol is tightly linked to the availability of methylation substrates [44, 45] and is well known for its roles in cell signaling and membrane dynamics, including those of the plasma membrane, endosomes, lysosomes, and the Golgi apparatus [46, 47]. Several phosphatidylinositol phosphates and their associated enzymes have also been detected in the nucleus [48, 49], although their nuclear functions remain poorly understood. For instance, the nuclear localization of phosphatidylinositol 4,5‐bisphosphate (PIP2) changes during the cell cycle [50, 51], yet the basis for this behavior is still unclear. Our chromatome DIA‐MS analysis revealed the presence of numerous PIP2‐related enzymes on chromatin across the cell cycle, and their nuclear localization appears to extend into the regulation of histone methylation (Figure 3A). Analysis of OpenCell [52] showed that many of these enzymes interact with nuclear proteins, reinforcing their nuclear localization (Figure S7A). To confirm the presence of nuclear and cell cycle‐dependent PIP2 metabolism, we characterized the cell cycle‐associated behavior of phosphatidylinositol 4‐phosphate 5‐kinase type 1 alpha (PIP5K1A), which phosphorylates phosphatidylinositol 4‐phosphate (PI4P) to PIP2 [53, 54] and is thus a central component of PIP2 metabolism. Chromatome DIA‐MS analysis showed that PIP5K1A was associated with chromatin predominantly in G1 and G2 (Figure 3A), with a marked increase from S to G2 (Figure 3B; Figure S5A, Cluster 2). High‐throughput immunofluorescence using a PIP5K1A‐specific antibody in unsynchronized U2OS FUCCI‐3 cells (Figure S7B) revealed that its nuclear concentration rose from G1 to S and again in G2/M (Figure 3C,D; Figure S7C), mirroring the increase in its total nuclear abundance across the cell cycle (Figure S7D). Thus, both chromatin‐bound and nuclear pools of PIP5K1A display a consistent rise from S to G2. To validate these observations, we sorted U2OS FUCCI‐3 cells into S or G2, isolated cytosolic, nucleoplasmic, and chromatin fractions, and analyzed them by Western blot. PIP5K1A levels increased in both the nucleoplasm and chromatin from S to G2 (Figure 3E), suggesting that PIP5K1A is reloaded onto chromatin following DNA replication. We next examined PLCD3 and PLD2, two PIP2‐related metabolic enzymes downstream of PIP5K1A. Both enzymes rely on PIP2, PLCD3 uses it as a substrate, and PLD2 requires it for activation [55, 56], thereby drawing from the same lipid pool. Chromatome DIA‐MS showed that PLCD3 was enriched on chromatin at the beginning of the cell cycle and again in M phase (Figure 3A,B; Figure S5A, Cluster 6), whereas PLD2 levels decreased in mitosis (Figure 3A,B; Figure S5A, Cluster 2). High‐throughput immunofluorescence in U2OS FUCCI‐3 cells revealed that the nuclear concentrations of both proteins increased from G1 through S and G2/M in parallel with their total nuclear abundance (Figure 3F–I; Figure S7E–J), in parallel with their total nuclear abundance. Given that these enzymes compete for PIP2, these findings suggest that their cell cycle–associated functions may be regulated not only by nuclear localization but also by distinct subnuclear compartmentalization. The differential presence of PIP2‐related enzymes in the chromatin and nuclear environment during cell cycle progression, prompted us to quantify the level of PIP2 itself in the nucleus by integrating the quantification of the FUCCI signal (Figure S7K) with a PIP2‐specific antibody (Figure 3J,K). Nuclear PIP2 concentration remained largely stable from G1 to G2/M and increased in M phase (Figure 3J,K; Figure S7L), whereas total nuclear PIP2 abundance progressively increased throughout the cell cycle (Figure S7M). PIP2‐related enzymes and PIP2 itself also showed higher nuclear levels in G0 than in G1 (Figure 3C–K). Similar cell cycle–associated patterns were observed in MCF7 FUCCI‐3 cells for PIP5K1A, PLCD3, and PLD2 (Figure S8A–L). In contrast, nuclear PIP2 concentration decreased across the cycle in these cells (Figure S8M–O) and the total PIP2 remained stable (Figure S8P).

Altogether, our data demonstrates that PIP2 metabolism is actively regulated across the cell cycle within the nuclear compartment (Figure 3L). PIP5K1A, PLCD3, and PLD2 show distinct nuclear and chromatin‐associated dynamics, accompanied by coordinated changes in nuclear PIP2 levels (Figure 3M). These findings reveal a structured nuclear PIP2 metabolic network that varies with cell cycle state.

### 2.4 Nuclear PIP2 and PIP5K1A Across Healthy Tissues

**FIGURE 4:**
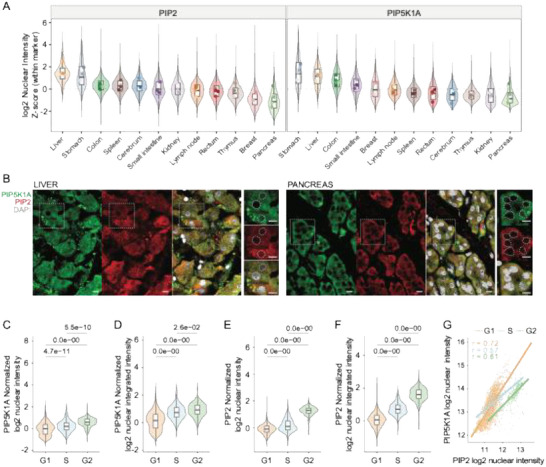
Phosphatidylinositol metabolism oscillates in the nucleus during the cell cycle in healthy tissues. (A) Immunofluorescence‐based quantification of nuclear PIP2 and PIP5K1A log2‐transformed mean intensity in TMA of healthy tissues. (B) Representative images of PIP5K1A and PIP2 inside the nucleus during cell cycle progression in TMA of healthy liver and pancreas tissues. Scale bar is 10 µm. (C) Immunofluorescence‐based quantification of nuclear PIP5K1A mean intensity in healthy liver tissue with proliferating cells, across cell cycle phases determined by DAPI staining (one‐way ANOVA with Tukey's HSD post hoc test). (D) Immunofluorescence‐based quantification of nuclear PIP5K1A integrated intensity in healthy liver tissue with proliferating cells, across cell cycle phases determined with the DAPI staining (one‐way ANOVA with Tukey's HSD post hoc test). (E) Immunofluorescence‐based quantification of nuclear PIP2 mean intensity in healthy liver tissue with proliferating cells, across cell cycle phases determined with the DAPI staining (unpaired two‐tailed Wilcoxon test). (F) Immunofluorescence‐based quantification of nuclear PIP2 integrated intensity in healthy liver tissue with proliferating cells, across cell cycle phases determined with the DAPI staining (one‐way ANOVA with Tukey's HSD post hoc test). (G) Immunofluorescence‐based correlation of nuclear PIP5K1A and PIP2 mean intensities in healthy liver tissue with proliferating cells, stratified by cell cycle phase determined by DAPI staining (Pearson correlation).

Cancer cell lines frequently undergo metabolic rewiring as part of malignant transformation. To assess whether the phosphatidylinositol enzymes we identified as cell cycle regulated in cancer cells are similarly regulated in normal tissues, we performed immunofluorescence staining for PIP2 and PIP5K1A on a multi‐organ tissue microarray (TMA) of healthy tissues (Figure. S9A). To reduce confounding by tissue architecture and cell‐type heterogeneity, we applied morphology‐ and intensity‐based filtering (Figure. S9B). After log2 transformation, per‐core normalization and outlier removal, DAPI, PIP2 and PIP5K1A intensities formed unimodal distributions suitable for cross‐tissue comparison (Figure. S9C). In line with our observations in U2OS cells, we detected nuclear staining of both PIP2 and PIP5K1A in multiple tissue types (Figure 4A,B). Among analysed tissues, liver nuclei displayed the highest nuclear PIP2 signal and the second highest PIP5K1A signal (Figure 4A,B; Figure S9D), whereas pancreatic tissue consistently showed lower nuclear levels of both PIP2 and PIP5K1A than the other tissues (Figure 4A,B; Figure S9D). To investigate cell cycle–dependent nuclear localization of PIP2 and PIP5K1A in situ, we next searched for TMA cores containing proliferating cells based on DAPI intensity distributions. One liver core exhibited a bimodal DAPI distribution characteristic of a cycling population (Figure S9E). For this core, we fit a two‐component Gaussian mixture model to the DAPI signal (Figure S9F), transformed it into DNA content units, and classified nuclei into G1, S, and G2 phases (Figure S9G). As in U2OS and MCF7 FUCCI‐3 cell lines, both the concentration and integrated nuclear intensity of PIP5K1A increased progressively as cells progressed through the cell cycle (Figure 4C,D). PIP2 concentration and total nuclear amount also rose toward G2 (Figure 4E,F), following a pattern like the U2OS dynamics. Furthermore, the concentration of PIP2 positively correlates with PIP5K1A concentration in all phases (Figure 4G), suggesting that PIP2 amounts depend on PIP5K1A across the cycle, and that PIP2 levels can be regulated by the fine tuning of PIP5K1A nuclear localization.

Overall, this analysis showed that nuclear PIP2 metabolism is not restricted to cancer cell lines but is also present in healthy human tissue.

### 2.5 Perturbation of Nuclear PIP5K1A Alters Nuclear PIP2 Distribution

Immunofluorescence revealed that PIP2 displays distinct localization patterns across nuclear compartments (Figure S10A), forming small, intense foci within fibrillarin‐labelled nucleoli and larger, less intense foci in fibrillarin‐negative non‐nucleolar regions, as previously reported [57, 58, 59, 60, 61]. We observed that nucleolar area increased with cell cycle progression in FUCCI‐3 U2OS cells (Figure S10B,C). The total nuclear amount of fibrillarin increased in a similar proportion to the nuclear size during cell cycle progression (Figure S10D) while its concentration in the nucleolus remains more stable (Figure S10E). Together, these data indicate that nucleolar area expands as cells progress through the cell cycle while fibrillarin concentration within nucleoli is maintained, supporting its use as a robust nucleolar proxy. Notably, G0 cells were characterized by larger, fibrillarin‐intense foci compared with G1 cells (Figure S10B–E).

Based on these observations, we integrated Hoechst staining to infer cell cycle phases (Figure S10G) and co‐stained fibrillarin and PIP2 to examine nuclear and nucleolar PIP2 dynamics across the cell cycle. Using this approach, fibrillarin‐labelled nucleolar area increased progressively both in U2OS and MCF7 cells (Figure S10G,H), validating this method for approximating cell cycle phases, albeit with lower resolution than the FUCCI‐3 system. Quantification of PIP2 levels revealed that PIP2 concentration remained stable both within and outside nucleolar regions throughout cell cycle progression (Figure S10I,J). The average area of individual PIP2 foci changed modestly across phases and followed opposite trends inside versus outside nucleoli (Figure S10K). Moreover, the number of PIP2 foci, normalized to the area of their respective compartments, slightly decreased from G1 to S phase and remained stable from S to G2 (Figure S10L,M). These data suggest that nuclear PIP2 is stably compartmentalized into distinct nucleolar and non‐nucleolar pools that are maintained across the cell cycle despite increases in nuclear and nucleolar size.

**FIGURE 5:**
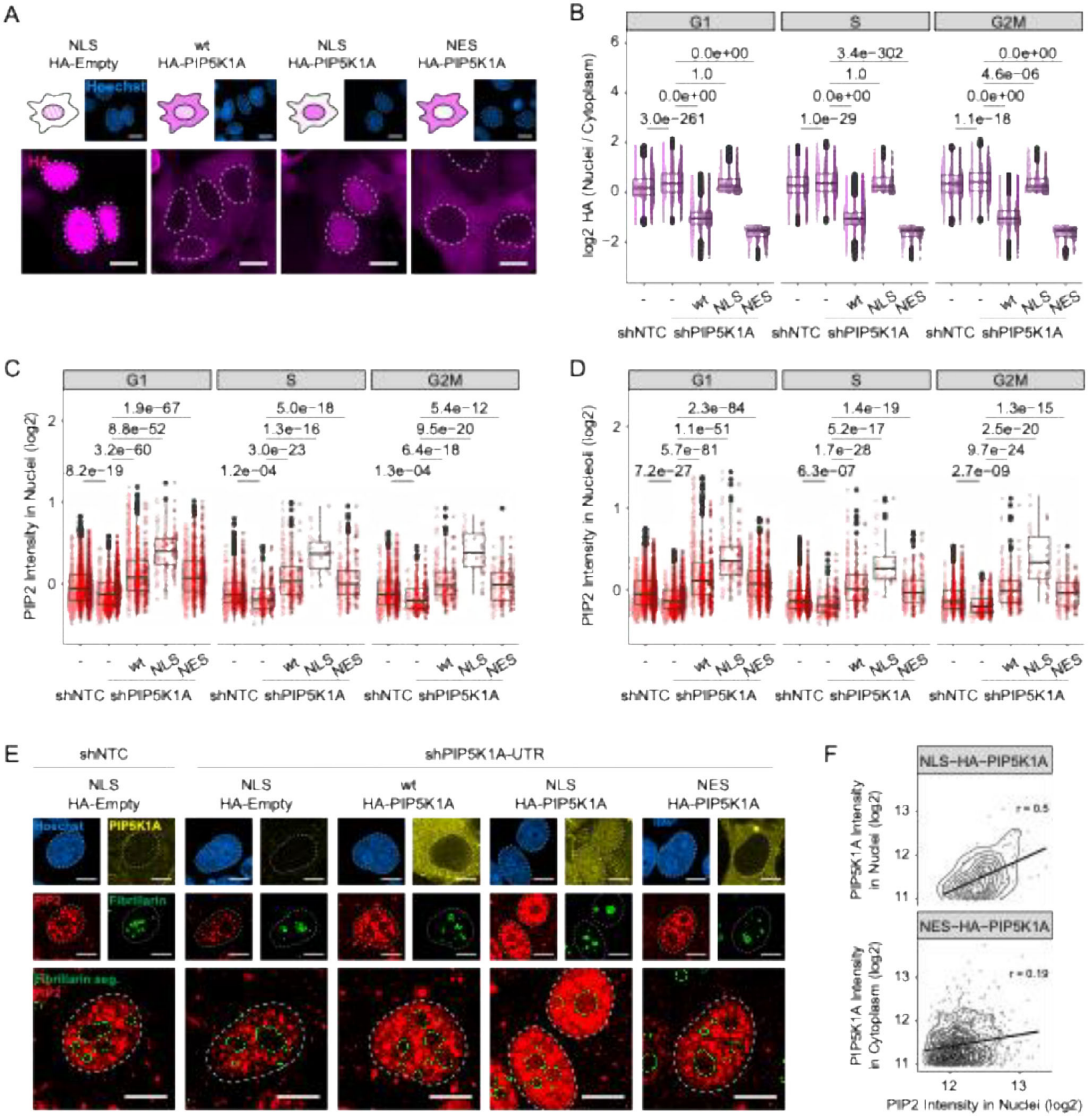
PIP2 nucleolar localization is dependent on nuclear phosphatidylinositol metabolism and cell cycle regulation. (A) Schematics and representative images of MCF7 expressing HA‐tagged PIP5K1A wt, NLS or NES variants. Scale bar is 10 µm. (B) Immunofluorescence‐based quantification nuclear/cytoplasmic ratio from HA mean intensities across cell cycle phases using Hoechst integrated intensity in MCF7 expressing shPIP5K1A‐UTR and HA‐tagged PIP5K1A‐wt, NLS or NES variants. shNTC and NLS‐HA‐Empty were used as negative control for knock‐down and overexpression respectively. 3 biological replicates were analyzed (*n*_shNTC_HA‐Empty*_*G1  =  23579, *n*_shPIP5K1A_HA‐Empty*_*G1  =  20637, *n*_shPIP5K1A_HA‐PIP5K1A‐wt*_*G1  =  12521, *n*_shPIP5K1A_HA‐PIP5K1A‐NLS*_*G1  =  2571, *n*_shPIP5K1A_HA‐PIP5K1A‐NES*_*G1  =  1700, *n*_shNTC_HA‐Empty*_*S  =  8630, *n*_shPIP5K1A_HA‐Empty*_*S  =  5886, *n*_shPIP5K1A_HA‐PIP5K1A‐wt*_*S  =  4501, *n*_ shPIP5K1A _HA‐PIP5K1A‐NLS*_*S  =  761, *n*_ shPIP5K1A _HA‐PIP5K1A‐NES*_*S  =  502, *n*_shNTC_HA‐Empty*_*G2  =  11737, *n*_shPIP5K1A_HA‐Empty*_*G2  =  8035, *n*_shPIP5K1A_HA‐PIP5K1A‐wt*_*G2  =  6184, *n*_shPIP5K1A_HA‐PIP5K1A‐NLS*_*G2  =  1280, *n*_ shPIP5K1A _HA‐PIP5K1A‐NES*_*G2  =  694; outliers removed, 3 SD; unpaired two‐tailed Wilcoxon test). (C) Immunofluorescence‐based quantification of nuclear PIP2 mean intensities across cell cycle phases using Hoechst integrated intensity in MCF7‐wt expressing shPIP5K1A‐UTR and HA‐tagged PIP5K1A‐wt, NLS or NES variants. shNTC and HA‐Empty were used as negative control for knock‐down and overexpression respectively. 3 biological replicates were analyzed (*n*_shNTC_HA‐Empty*_*G1  =  2613, *n*_shPIP5K1A_HA‐Empty*_*G1  =  1007, *n*_shPIP5K1A_HA‐PIP5K1A‐wt*_*G1  =  625, *n*_shPIP5K1A_HA‐PIP5K1A‐NLS*_*G1  =  115, *n*_shPIP5K1A_HA‐PIP5K1A‐NES*_*G1  =  836, *n*_shNTC_HA‐Empty*_*S  =  856, *n*_shPIP5K1A_HA‐Empty*_*S  =  245, *n*_shPIP5K1A_HA‐PIP5K1A‐wt*_*S  =  243, *n*_shPIP5K1A_HA‐PIP5K1A‐NLS*_*S  =  33, *n*_shPIP5K1A_HA‐PIP5K1A‐NES*_*S  =  210, *n*_shNTC_HA‐Empty*_*G2  =  667, *n*_shPIP5K1A_HA‐Empty*_*G2  =  255, *n*_shPIP5K1A_HA‐PIP5K1A‐wt*_*G2  =  195, *n*_shPIP5K1A_HA‐PIP5K1A‐NLS*_*G2  =  42, *n*_shPIP5K1A_HA‐PIP5K1A‐NES*_*G2  =  163; outliers removed, 3 SD; unpaired two‐tailed Wilcoxon test). (D) Immunofluorescence‐based quantification of nucleolar PIP2 mean intensities across cell cycle phases using Hoechst integrated intensity in MCF7‐wt expressing shPIP5K1A‐UTR and HA‐tagged PIP5K1A‐wt, NLS or NES variants. shNTC and HA‐Empty were used as negative control for knock‐down and overexpression respectively. 3 biological replicates were analyzed (*n*_shNTC_HA‐Empty*_*G1  =  2613, *n*_shPIP5K1A_HA‐Empty*_*G1  =  1008, *n*_shPIP5K1A_HA‐PIP5K1A‐wt*_*G1  =  624, *n*_shPIP5K1A _HA‐PIP5K1A‐NLS*_*G1  =  114, *n*_shPIP5K1A_HA‐PIP5K1A‐NES*_*G1  =  831, *n*_shNTC_HA‐Empty*_*S  =  858, *n*_shPIP5K1A_HA‐Empty*_*S  =  245, *n*_shPIP5K1A_HA‐PIP5K1A‐wt*_*S  =  242, *n*_shPIP5K1A _HA‐PIP5K1A‐NLS*_*S  =  33, *n*_ shPIP5K1A_HA‐PIP5K1A‐NES*_*S  =  208, *n*_shNTC_HA‐Empty*_*G2  =  671, *n*_shPIP5K1A_HA‐Empty*_*G2  =  253, *n*_shPIP5K1A _HA‐PIP5K1A‐wt*_*G2  =  195, *n*_ shPIP5K1A _HA‐PIP5K1A‐NLS*_*G2  =  42, *n*_ shPIP5K1A _HA‐PIP5K1A‐NES*_*G2  =  162; outliers removed, 3 SD; unpaired two‐tailed Wilcoxon test). (E) Representative confocal images showing the intensity and distribution of Fibrillarin (green), PIP2 (red) and PIP5K1A (yellow) in MCF7 expressing shPIP5K1A‐UTR and HA‐tagged PIP5K1A wt, NLS or NES variants. shNTC and HA‐Empty were used as negative control for knock‐down and overexpression respectively. The nucleus, identified with Hoechst staining, is shown in blue. Scale bar is 10 µm. (F) Scatter plot with linear regression and Pearson r showing the correlation between PIP2 nuclear intensity and PIP5K1A intensity at the intended localization: nuclear for NLS (top) and cytoplasmic for NES (bottom). 2D density contours indicate point density.

To test the role of PIP5K1A in maintaining nuclear PIP2 levels during the cell cycle, we performed UTR‐targeted PIP5K1A knockdown in MCF7 wild‐type cells, followed by rescue with HA–FLAG‐tagged PIP5K1A WT, PIP5K1A‐NLS, or PIP5K1A‐NES variants (Figure 5A). All rescue constructs exhibited the expected nuclear or cytoplasmic HA enrichment (Figure 5B; Figure S11A–D). Downregulation of PIP5K1A reduced PIP2 abundance in both nucleolar and non‐nucleolar compartments (Figure 5C–E). Although PIP2 levels in both compartments were restored by overexpression of either construct, cells rescued with the nuclear‐localized PIP5K1A‐NLS displayed a markedly greater increase in nucleolar and non‐nucleolar PIP2 abundance across all cell‐cycle phases (Figure 5C–E). Accordingly, nuclear PIP5K1A‐NLS abundance showed a substantially stronger correlation with nuclear PIP2 levels than the cytoplasmic levels of PIP5K1A‐NES (Figure 5F).

To assess functional sensitivity to reduced PIP2 synthesis, we inhibited PIP5K1A with ISA‐2011B in U2OS FUCCI‐3 cells. IC50 determination over 72 h (Figure S12A) identified doses that impaired proliferation (Figure S12B,C). Live‐cell imaging revealed rapid accumulation of cells in G1 upon inhibition (Figure S12D) and extended residence in the G2/M gate for cells initially in this stage (Figure S12E), in line with the phase‐dependent chromatin association of PIP5K1A (Figure 3A,E). A 48‐h treatment, selected to capture early effects while minimizing cytotoxicity (Figure S12F), caused a modest increase in nuclear PIP5K1A (Figure S12G–I). While overall nuclear PIP2 levels were unchanged (Figure S12J), nucleolar PIP2 concentrations decreased significantly in G1 and G2/M (Figure S12K,L), corresponding to phases in which PIP5K1A normally associates with chromatin.

Together, these results show that nuclear PIP2 distribution changes in a subnuclear‐specific manner during the cell cycle and that perturbing PIP5K1A affects both cell cycle progression and nucleolar PIP2 levels.

### 2.6 A Functional Link Between Nuclear PIP2 Metabolism and Chromatin Methylation

**FIGURE 6:**
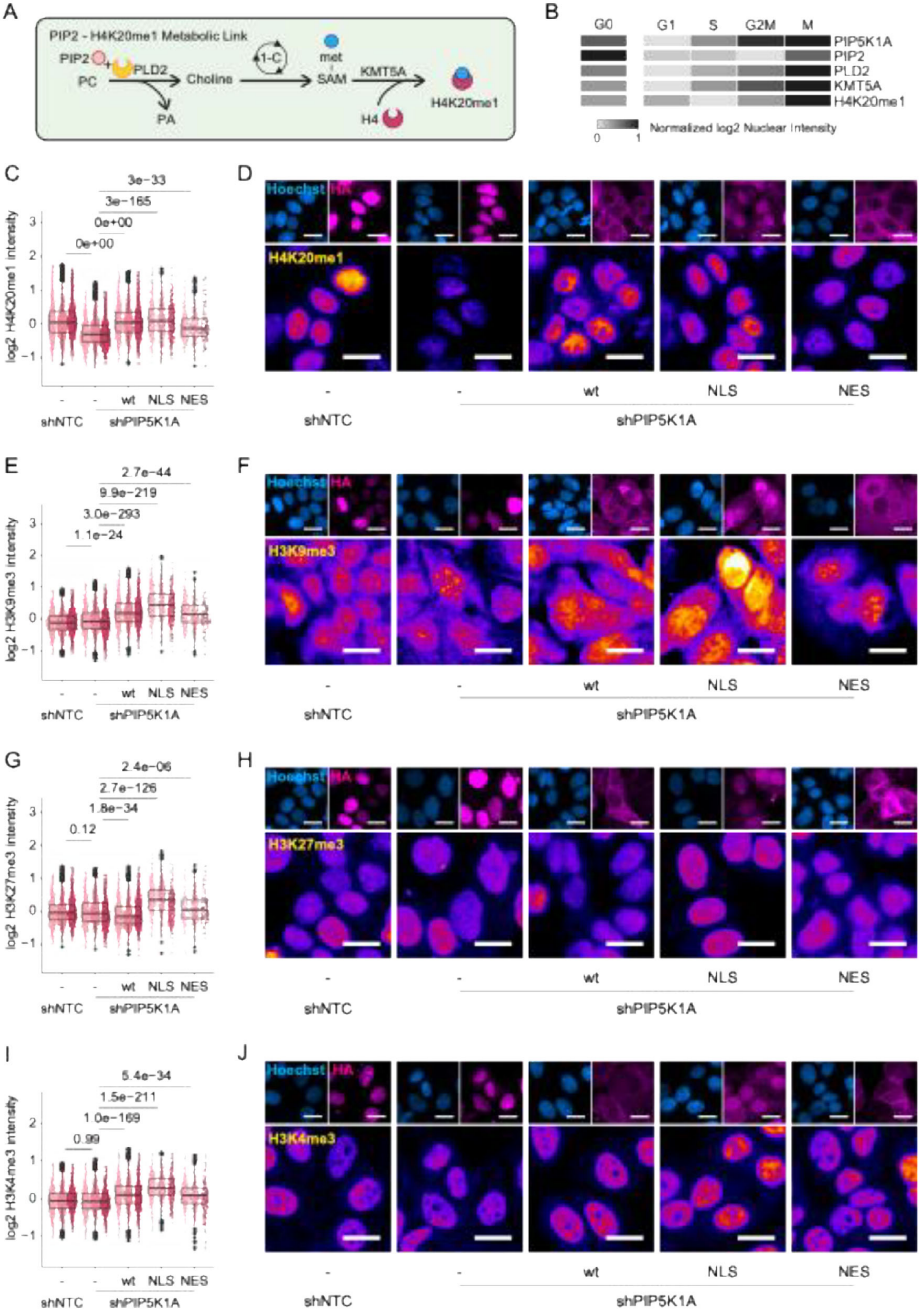
PIP2 perturbations differentially affect histone methylation. (A) Schematics of metabolic link between PIP2 and H4K20me1: PIP2‐activated PLD2 hydrolyzes phosphatidylcholine (PC) to generate phosphatidic acid (PA) and free choline. Choline feeds into one‐carbon (1C) metabolism to support S‐adenosylmethionine (SAM) synthesis, the methyl donor used by KMT5A to deposit H4K20 methylation. (B) Heatmap representing phosphatidylinositol metabolism and H4K20me1 deposition in the nucleus during cell cycle obtained by plotting the normalized mean intensity in log2 scale measured by immunofluorescence in MCF7 FUCCI‐3 cells. (C) Immunofluorescence‐based quantification of H4K20me1 intensity in nuclei region from MCF7 expressing shPIP5K1A‐UTR and HA‐tagged PIP5K1A wt, NLS or NES variants. 3 biological replicates were analyzed (*n*_shNTC_HA‐Empty  =  12920, *n*_shPIP5K1A_HA‐Empty  =  8778, *n*_ shPIP5K1A_HA‐PIP5K1A‐wt  =  6403, *n*_ shPIP5K1A_HA‐PIP5K1A‐NLS  =  1342, *n*_ shPIP5K1A_HA‐PIP5K1A‐NES =  763; outliers removed, 3 SD; unpaired two‐tailed Wilcoxon test). (D) Representative images showing the intensity and distribution of H4K20me1 (Fire) and HA‐tag (magenta) in MCF7 expressing shPIP5K1A‐UTR and HA‐tagged PIP5K1A wt, NLS or NES variants. shNTC and HA‐Empty were used as negative control for knock‐down and overexpression respectively. The nucleus, identified with Hoechst staining, is shown in blue. Scale bar is 10 µm. (E) Immunofluorescence‐based quantification of H3K9me3 intensity in nuclei region from MCF7 expressing shPIP5K1A‐UTR and HA‐tagged PIP5K1A wt, NLS or NES variants. 3 biological replicates were analyzed (*n*_shNTC_HA‐Empty  =  11479, *n*_shPIP5K1A_HA‐Empty  =  8724, *n*_shPIP5K1A_HA‐PIP5K1A‐wt  =  5628, *n*_shPIP5K1A_HA‐PIP5K1A‐NLS  =  1035, *n*_shPIP5K1A_HA‐PIP5K1A‐NES =  715; outliers removed, 3 SD; unpaired two‐tailed Wilcoxon test). (F) Representative images showing the intensity and distribution of H3K9me3 (Fire) and HA‐tag (magenta) in MCF7 expressing shPIP5K1A‐UTR and HA‐tagged PIP5K1A wt, NLS or NES variants. shNTC and HA‐Empty were used as negative control for knock‐down and overexpression respectively. The nucleus, identified with Hoechst staining, is shown in blue. Scale bar is 10 µm. (G) Immunofluorescence‐based quantification of H3K27me3 intensity in nuclei region from MCF7 expressing shPIP5K1A‐UTR and HA‐tagged PIP5K1A wt, NLS or NES variants. 3 biological replicates were analyzed (*n*_shNTC_HA‐Empty  =  9434, *n*_shPIP5K1A_HA‐Empty  =  8306, *n*_shPIP5K1A_HA‐PIP5K1A‐wt  =  5907, *n*_shPIP5K1A_HA‐PIP5K1A‐NLS  =  1085, *n*_shPIP5K1A_HA‐PIP5K1A‐NES =  510; outliers removed, 3 SD; unpaired two‐tailed Wilcoxon test). (H) Representative images showing the intensity and distribution of H3K27me3 (Fire) and HA‐tag (magenta) in MCF7 expressing shPIP5K1A‐UTR and HA‐tagged PIP5K1A wt, NLS or NES variants. shNTC and HA‐Empty were used as negative control for knock‐down and overexpression respectively. The nucleus, identified with Hoechst staining, is shown in blue. Scale bar is 10 µm. (I) Immunofluorescence‐based quantification of H3K4me3 intensity in nuclei region from MCF7 expressing shPIP5K1A‐UTR and HA‐tagged PIP5K1A wt, NLS or NES variants. 3 biological replicates were analyzed (*n*_shNTC_HA‐Empty  =  10449, *n*_shPIP5K1A_HA‐Empty  =  8863, *n*_shPIP5K1A_HA‐PIP5K1A‐wt  =  5753, *n*_shPIP5K1A_HA‐PIP5K1A‐NLS  =  1229, *n*_shPIP5K1A_HA‐PIP5K1A‐NES =  960; outliers removed, 3 SD; unpaired two‐tailed Wilcoxon test). (J) Representative images showing the intensity and distribution of H3K4me3 (Fire) and HA‐tag (magenta) in MCF7 expressing shPIP5K1A‐UTR and HA‐tagged PIP5K1A wt, NLS or NES variants. shNTC and HA‐Empty were used as negative control for knock‐down and overexpression respectively. The nucleus, identified with Hoechst staining, is shown in blue. Scale bar is 10 µm.

Nucleoli are integral to the distribution and function of heterochromatin in the nucleus, serving as hubs that facilitate chromatin organization and silencing [62, 63]. Because PIP2 behaved differently inside nucleoli upon PIP5K1A inhibition, we examined whether chromatin‐associated histone methylation marks were affected. We first analyzed Histone 4 lysine 20 monomethylation (H4K20me1), which accumulates at centromeres during mitosis [64, 65]. This choice was supported by the observation that PIP5K1A levels increase in the nucleus during G2/M (Figure 3C,D; Figures S7B–D and S8A–D), while nuclear PIP2 levels show a slight decrease (Figure 3J,K; Figures S7K–M and S8M–P), indicating active PIP2 production and usage during this transition. Notably, both nuclear PIP5K1A and PIP2 reach their highest levels in mitosis (Figure 3J,K; Figures S7K–M and S8M–P), consistent with an elevated requirement for PIP2 in this phase. In parallel, KMT5A, the sole writer of the H4K20me1 mark [66, 67], also peaks on chromatin in G2/M and mitosis (Figure 2C–E), further reinforcing the connection between PIP5K1A, PIP2 dynamics, and H4K20 monomethylation (Figure 6A). As controls, we also examined the constitutive heterochromatin mark H3K9me3 and the facultative heterochromatin mark H3K27me3. In untreated U2OS cells, H4K20me1 increased progressively from S phase and reached a maximum in M phase (Figure S13A–D) coinciding with the maximum concentration of PIP5K1A, PIP2 and KMT5A in the nucleus (Figure 6B); these results were reproduced in MCF7 cells (Figure S13E–H). After 48 h of PIP5K1A inhibition, H4K20me1 levels were reduced in all phases except G2/M and M (Figure S14A–C). H3K9me3 decreased slightly from G1 to G2/M and increased in M under untreated conditions (Figure S14D–F), and its levels significantly increased from G1 to G2/M phases after PIP5K1A inhibition (Figure S14E,F). H3K27me3 showed a similar pattern: modestly reduced from G1 to G2/M and increased in M in untreated cells (Figure S14G–I), with higher levels across G1 to G2/M following PIP5K1A inhibition (Figure S14H,I).

Since ISA‐2011B Inhibits PIP5K1A throughout the Cell, We next Asked Whether Histone‐mark Changes Depended Specifically on Nuclear versus Cytoplasmic PIP5K1A. We Therefore Performed Immunofluorescence for the Same Histone Marks in UTR‐targeted PIP5K1A Knockdown MCF7 Cells Rescued With HA‐Flag–tagged PIP5K1A Wild Type, PIP5K1A‐NLS, or PIP5K1A‐NES (Figure 5E).

H4K20me1 levels Were Reduced After shPIP5K1A Compared With shNTC (Figure 6C,D), Consistent With ISA‐2011B Treatment. Reduction of H4K20me1 levels Following PIP5K1A Downregulation Was Not Attributable to Cell Cycle Arrest, as the Decrease Was Observed Across all Cell Cycle Phases (Figure S15A). This Reduction Was Fully Rescued by WT and NLS‐localized PIP5K1A, but Only Partially by the NES Variant (Figure 6C,D), Indicating That Nuclear PIP5K1A Is More Effective Than Cytoplasmic PIP5K1A in Restoring H4K20me1. H3K9me3 Showed minor Changes Upon shPIP5K1A (Figure S15B) but Increased Strongly Upon Expression of Exogenous PIP5K1A, With the Largest Increase Observed With the NLS Variant (Figure 6E,F). H3K27me3 Was Unaffected by shPIP5K1A (Figure S15C) and Strongly Increased Upon Expression of PIP5K1A‐NLS (Figure 6G,H).

To Further Assess Whether these Changes Reflected a General Increase in Histone Methylation Capacity, We Analyzed H3K4me3, a Mark of Active Chromatin. H3K4me3 Levels Were Unchanged Upon PIP5K1A Knockdown (Figure S15D) but Increased Upon Rescue With Exogenous PIP5K1A, Especially With Nuclear Targeting (Figure 6I,J).

The patterns of H3K9me3, H3K27me3, and H3K4me3 were therefore consistent with more efficient deposition of these marks upon nuclear enrichment of PIP5K1A; however, only H4K20 monomethylation was reduced following PIP5K1A downregulation, demonstrating specificity for this modification.

In conclusion, the data indicates a link between nucleolar composition, nuclear PIP2 metabolism, and histone methylation during the cell cycle. PIP5K1A activity and its nuclear localization are functionally connected to H4K20 monomethylation and cell cycle progression, while nuclear enrichment of PIP5K1A enhances multiple histone methylation marks without being required for their basal maintenance.

## 3 Discussion

Recent studies in transcriptomics and proteomics have investigated cell cycle oscillations and demonstrated that gene expression and protein synthesis change during the cell cycle [68, 69, 70, 71, 72]. However, despite the importance of protein compartmentalization in regulating protein function [73], protein compartmentalization in the context of cell cycle regulation has not been studied systematically. Given the recent focus on nuclear compartmentalization of metabolic enzymes in chromatin functions [23, 24, 25, 26, 27, 28, 29, 30, 31, 74] here we asked whether metabolic enzymes could be relocated either on chromatin or in the nucleus in a cell cycle–dependent manner. By integrating high‐throughput immunofluorescence and mass spectrometry, we established an unbiased approach that discriminates sub pools of nuclear metabolic enzymes localized either in the nucleoplasm or on chromatin across defined cell cycle phases. Moreover, by focusing on PIP2 metabolism as a showcase, we demonstrate that phase‐specific nuclear and subnuclear PIP2 accumulation correlates with changes in histone methylation and cell cycle progression, suggesting that nuclear PIP2 metabolism may interface with chromatin regulation.

In this study, we generated stable FUCCI‐3 reporters enabling long‐term tracking of cell cycle progression without impairing proliferation (Figure S3A–G). Unlike other studies [75, 76, 77, 78, 79, 80], our FUCCI‐3 system allowed naïve sorting of cells in distinct cell cycle phases and transitions without the use of chemical arrestors, thereby avoiding artifacts associated with enforced cell cycle blockade. Using these sorted populations, we performed quantitative mass spectrometry on chromatin (the “chromatome”) to identify factors oscillating during the cell cycle. Because cell volume and total protein content increase as cells progress through the cycle [71], we performed the analysis using equal protein input; thus, our readouts reflect relative protein abundance rather than absolute changes in protein mass.

We found that the nuclear and chromatin localization of chromatin‐methylation regulators (Figure 2B) and phosphatidylinositol‐pathway enzymes (Figure 3B) are cell cycle regulated, including in human tissues (Figure 4C). Our workflow integrates nuclear imaging with chromatome data to track fluctuations between nuclear and chromatin pools of these enzymes. We analyzed Fibrillarin dynamics as a proxy for nucleolar behavior. Because PIP2 colocalizes with Fibrillarin (Figure 5A,B) and phosphatidylinositol synthesis and histone methylation vary across the cell cycle (Figures 3M and 6A–H), our data indicate a close association between subnuclear PIP2 localization and chromatin regulation. In particular, the rise of PIP5K1A in G2/M and M and the concurrent redistribution of nuclear PIP2 coincide with the accumulation of the mitotic mark H4K20me1 (Figure 6A). This relationship is supported by the observation that PIP5K1A depletion selectively reduces H4K20me1 levels in all phases without altering other methylation marks (Figure S15A–D), and that this reduction is fully rescued by wild‐type and nuclear‐targeted PIP5K1A, but only partially by the NES variant (Figure 6C,D). Moreover, forcing PIP5K1A into the nucleus increases several additional histone methylation marks, indicating that nuclear PIP2 synthesis enhances the methylation capacity of chromatin (Figure 6E–J). Together, these findings reveal that subnuclear PIP2 metabolism is dynamically reorganized during the cell cycle and is closely associated with the establishment of chromatin states required for mitotic progression.

We also detected cell cycle‐arrested (G0) cells using the FUCCI‐3 system (Figure 1B), which appear to contain both quiescent and senescent populations (Figure S1F,G). Interestingly, nuclear PIP5K1A, PLCD3, PLD2, and PIP2 itself were elevated in cells exhibiting a G0‐like phenotype (Figure 3C–K; Figure S7B–M), suggesting altered PIP2 metabolism in arrested states. These observations point to a relationship between nucleolar architecture and nuclear PIP2 metabolism and highlight that this relationship changes when cells exit the proliferative cycle. Furthermore, the association between PIP2 dynamics and histone methylation (Figure 6A–J) suggests that altered PIP2 metabolism or storage may accompany or reinforce changes in chromatin during cell cycle exit. In addition, because PIP2 has been associated with Fibrillarin and UBF in the nucleolus as part of the Pol I complex [57, 81], our findings raise the possibility that cell cycle regulated nuclear phosphatidylinositol‐pathway enzymes could influence the rRNA expression, needed for cell cycle progression, through the production of nucleolar PIP2.

Our study not only links protein compartmentalization to cell cycle progression but also indicates that specific subnuclear pools of metabolic enzymes and metabolites may be associated with cell states and fates. This establishes PIP2 metabolism as an additional layer of nuclear organization with potential relevance for proliferation and arrest. While in this study we observed PIP2 and PIP5K1A nuclear localization also in terminally differentiated human tissues (Figure 4A–E), in the future, it will be important to determine whether cancer cells exploit nuclear PIP2 metabolism to facilitate aberrant cell cycle re‐entry. Beyond PIP2 metabolism, advances in spatial metabolomics and subnuclear proteomics will be essential to uncover how enzymes and metabolites are dynamically compartmentalized through the cell cycle once current technical limitations in quantifying subnuclear metabolite pools are overcome.

## 4 Materials and Methods

### 4.1 Lentiviral Vectors

#### 4.1.1 FUCCI‐3 Lentiviral Vectors

To generate the FUCCI‐3 lentiviral plasmids, the neomycin and hygromycin coding regions driven by the SV40 promoter were PCR amplified from the pcDNA3 [82] and GW209_pCRIS‐PITChv2‐C‐d vectors [83], respectively, with primers 1 and 2 (Table 1), using Phusion High‐Fidelity DNA Polymerase (ThermoFisher Scientific; #F530). The resulting products were purified using the QIAquick Gel Extraction Kit (Qiagen, 28706). The PCR products and the backbones pLL3.7m‐mTurquoise2‐SLBP and pLL3.7m‐Clover‐Geminin(1‐110)‐IRES‐mKO2‐Cdt were cut with MluI‐HF (NEB, R3198S), followed by dephosphorylation with rSAP (NEB, M0371S). The cut vectors were gel‐purified using the QIAquick Gel Extraction Kit (Qiagen, 28706). The PCR products were cloned into each lentiviral vector overnight by classical ligation with T4 ligase (NEB, M0202S). The resulting ligation product was transformed in 25 µL of DH5α (Thermo Fisher Scientific, 18265017) and selected at 100 µg/mL in house ampicillin plates. Individual colonies were analyzed by Sanger sequencing using primers 3, 4, and 5 (Table 1). The coding sequence of Histone 1 fused to Maroon1 from pLL3.7m‐mTurquoise2‐SLBP was eliminated by excision of the vector with EcoRI‐HF (NEB, R3101S). After gel purification using the QIAquick Gel Extraction Kit (Qiagen, 28706), the resulting cut vector was religated overnight with T4 ligase (NEB, M0202S). The resulting ligation product was transformed with 25 µL of DH5α (Thermo Fisher Scientific, 18265017) and selected at 100 µg/mL in house ampicillin plates. Individual colonies were analyzed by Sanger sequencing using primer 6 (Table 1).

**TABLE 1:** Oligonucleotide primers and synthetic DNA sequences used for lentiviral vector generation.

Class	Name	Sequence 5'‐3'
Primer	Primer 1	caatgacttacaaggcagctgacgcgtgtgtgtcagttagggtgtggaaag
Primer	Primer 2	cttttcttttaaaatcacgcgttaagatacattgatgagtttggacaaacca
Primer	Primer 3	atcggcactttgcatcggc
Primer	Primer 4	gcagggtcgatgcgacg
Primer	Primer 5	cagcagctaccaatgctgatt
Primer	Primer 6	ctaggtacaattcgatatcaagcttatcg
Primer	Primer 7	caaagcatgcatctcaattagtcagcaaccaggtgtggaaagtccccagg
Primer	Primer 8	taagatacattgatgagtttggacaaacc
Geneblock	gb_bsd	gaggcctaggcttttgcaaaaagctcccgggagcttgtatatccattttcggatctgatcagcacgtgatggccaagcctttgtctcaagaagaatccaccctcattgaaagagcaacggctacaatcaacagcatccccatctctgaagactacagcgtcgccagcgcagctctctctagcgacggccgcatcttcactggtgtcaatgtatatcattttactgggggaccttgtgcagaactcgtggtgctgggcactgctgctgctgcggcagctggcaacctgacttgtatcgtcgcgatcggaaatgagaacaggggcatcttgagcccctgcggacggtgccgacaggtgcttctcgatctgcatcctgggatcaaagccatagtgaaggacagtgatggacagccgacggcagttgggattcgtgaattgctgccctctggttatgtgtgggagggctaacacgtgctacgagatttcgattccaccgccgccttctatgaaaggttgggcttcggaatcgttttccgggacgccggctggatgatcctccagcgcggggatctcatgctggagttcttcgcccaccccaacttgtttattgcagcttataatggttacaaataaagcaatagcatcac
Geneblock	PIPK5A	ggctacccatacgacgtcccagactacgctggccagggcgtacgtccacgcgggcgcgccgcgtcggcctcctccgggccgtcgtcttcggtcggtttttcatcctttgatcccgcggtcccttcctgtaccttgtcctcagcagcatctggaatcaagagacccatggcatctgaggtcttggaagctagacaggattcttacatctcattggtgccttatgcctctggcatgcccatcaagaaaataggccatagaagtgttgattcctcaggagagacaacatataaaaagacaacctcatcagccttgaaaggtgccatccagttaggcattacccacactgtggggagcctgagtaccaaaccagagcgtgatgtcctcatgcaagatttctacgtggttgagagtatcttctttcccagtgaagggagcaacctgacccctgctcatcactacaatgactttcgtttcaagacctatgcacctgttgccttccgctacttccgggagctatttggtatccggcccgatgattacttgtattccctctgcagtgagccgctgattgaactctgtagctctggagctagtggttccctattctatgtgtccagcgacgatgagttcattattaagacagtccaacataaagaggcggaatttctgcagaagctgcttccaggatactacatgaacctcaaccagaaccctcggactttgctgcctaaattctatggactgtactgtgtgcaggcaggtggcaagaacattcggattgtggtgatgaacaatcttttaccaagatcggtaaaaatgcatatcaaatatgacctcaaaggctcaacctacaaacggcgggcttcccagaaagagcgagagaagcctcttcccacatttaaagacctagacttcttacaagacatccctgatggtctttttttggatgctgacatgtacaacgctctctgtaagaccctgcagcgtgactgtttggtgctgcagagcttcaagataatggattacagcctcttgatgtcaatccataatatagatcatgcacaacgagagcccttaagcagtgaaacacagtactcagttgatactcgaagaccggccccccaaaaggctctgtattccacagccatggaatccatccagggagaggctcgacggggtggtaccatggagactgatgaccatatgggtggcatccctgcccggaatagtaaaggggaaaggcttctgctttatattggcatcattgacattctacagtcttacaggtttgttaagaagttggagcactcttggaaagccctggtacatgacggagacactgtctcagtgcatcgcccaggcttctacgctgaacggttccagcgcttcatgtgcaacacagtatttaagaagattcccttgaagccttctccttccaaaaagtttcggtctggctcatctttctctcggcgagcaggctccagtggcaactcctgcattacttaccagccatcggtctctggggaacacaaggcacaagtgacaacaaaggcagaagtggagccaggcgttcaccttggtcgtcctgatgttttacctcagactccacctttggaggaaatcagtgagggctcgcctattcctgaccccagtttctcacctctagttggagagactttgcaaatgctaactacaagtacaaccttggaaaagcttgaagttgcagagtcagagttcacccattaagaattcgtcgagggacctaataacttcgtatagcatacattatac

#### 4.1.2 Knock‐Down Lentiviral Vectors

To induce knock‐down of PIP5K1A, lentiviral vector containing pre‐designed shRNA targeting the 3’UTR sequence from mRNA (NM_003557, RefSeq) was obtained from bacterial glycerol stock (Merck, clone TRCN0000231481). A non‐targeting shRNA (shNTC, 5′‐ CTTACGCTAGTACTTCGA‐3′) cloned into pLKO.1 plasmid with Puromycin resistance was used as control.

#### 4.1.3 Over‐Expression Lentiviral Vectors

To obtain the lentiviral plasmids containing the HA‐WT‐PIP5KA, 3xNLS‐HA‐PIP5KA, NES‐HA‐PIP5KA gene and the control lentiviral plasmid without PIP5KA referred as EMPTY‐3NLS‐HA‐BSD, different strategies were followed. The plasmids KO‐WT, KO‐NLS, KO‐NES, and KO‐EMPTY [27] were modified changing the antibiotic resistance from Puromycin to Blasticidine. Plasmids were cut with XmaI (New England Biolabs; # R0180S) and PsiI (New England Biolabs; # R0744S), and a geneblock containing the bsd gene was ordered (gb_bsd) and cloned in the cut vector via Gibson assembly in the purified vector for 3 h at 50°C followed by DH5α E. coli competent cells (Thermo Fisher Scientific; #18265017). Single clones were sequenced using primers 7 and 8 (Table 1). The obtained vectors were cut with AscI (New England Biolabs; # R0558S) and EcoRI‐HF (New England Biolabs; # R3101S) and were purified with QIAquick PCR & Gel Cleanup Kit (Qiagen; #28506). A geneblock containing the PIP5KA coding sequence (Table 1) was ordered (IDT) and cloned into the purified vectors via Gibson assembly in the purified vector for 3 h at 50°C followed by DH5α E. coli competent cells (Thermo Fisher Scientific; #18265017). Single clones were full plasmid sequenced.

### 4.2 Cell Culture

All cell lines used in this study were cultured in Petri dishes at 37°C and 5% CO2 with DMEM (Gibco; #11966025) supplemented with 10% Fetal Bovine Serum (FBS) (Gibco; #10270106) and 1% penicillin/streptomycin (Gibco; #15140122).

### 4.3 Lentiviral Particles Production and Cell Transduction

Lentiviral particles were generated using HEK293T cells cultured in 150 mm dishes and transfected via a polyethyleneimine (PEI)‐mediated protocol (Polysciences; #23966‐1). Briefly, 5.5 µg of the pCMV‐dR8_91 plasmid and 4.2 µg of the pVSV‐G envelope plasmid were combined with 8.4 µg of the plasmid carrying the gene of interest in 1 mL of Opti‐MEM medium (Gibco; #11058021). Separately, 54.6 µL of 1 mg/mL PEI solution was diluted with 900 µL of Opti‐MEM. After 5 min, the plasmid mix and the PEI solution were combined, incubated for 20 min to form transfection complexes, and added dropwise to the HEK293T cells in DMEM (Gibco; #11966025) supplemented with 10% Fetal Bovine Serum (FBS, Gibco; #10270106) and 1% penicillin/streptomycin (Gibco; #15140122). Six hours later, the medium was replaced with fresh growth medium. Supernatant containing lentiviral particles was harvested 48 h post‐transfection. For lentiviral transduction of U2OS and MCF7 cells, 1 mL of viral supernatant was added per well of a 6‐well plate, along with Polybrene (Sigma‐Aldrich; #TR‐1003) at a final concentration of 10 µg/mL.

### 4.4 FUCCI‐3 Reporter Cell Line Generation

To establish a stable FUCCI‐3 system, U2OS cells and MCF7 cells were infected with lentiviral particles containing the modified plasmids derived from the original FUCCI‐4 system [10]: pLL3.7m‐mTurquoise2‐SLBP(18‐126)‐Neomycin and pLL3.7m‐Clover‐Geminin(1‐110)‐IRES‐mKO2‐Cdt(30‐120)‐Hygromycin. Following transduction, cells were cultured in media containing 200 µg/mL Geneticin (Thermo Scientific; #10131027) and 150 µg/mL Hygromycin (Sigma‐Aldrich; #H3274) to select for successfully transduced cells, with the selection process lasting four to seven days. Fluorescence‐activated cell sorting (FACS) using a BD Influx sorter was performed to enrich cells displaying proper FUCCI‐3 expression dynamics. This FUCCI‐3 system was specifically designed to track the cell cycle by monitoring three cell cycle‐regulated proteins: Clover‐Geminin, SLBP‐Turquoise2, and Cdt1‐mKO2. To ensure the long‐term correct expression of the FUCCI‐3 reporter system and homogeneity of the population, the FUCCI‐3 reporter cells were not maintained in culture for more than one month. Another batch of cells was then thawed and sorted for the expected color pattern of the FUCCI‐3 reporter system.

### 4.5 PIP5K1A Knock‐Down and Overexpression Cell Line Generation

To Achieve Combined Endogenous PIP5K1A Knock‐Down and Exogenous Overexpression, 30 µL Containing 1500 Cells MCF7 Cells Were Added in 384 Plates and Then Were Infected With 30 µL of Filtered Lentiviral Particles Expressing shRNA Targeting 3’UTR mRNA and 30 µL of Filtered Lentiviral Particles Expressing HA‐tagged PIP5K1A cDNA (CDS From NM_003557) and either no Additional C‐terminal Sequence or a C‐terminal NLS or NES. Infection's Negative Control for Knock‐Down or Overexpression Was Done by Adding 30 µL of Culture media. Cells Were Left Incubating Overnight, Washed 3 Times With PBS 1x and Left With 100 µL of Culture media Containing 1ug/Ml Puromycin (Sigma‐Aldrich; #P8833) and 1ug/Ml Blasticidine (Sigma‐Aldrich; #15205) for 4 Days

### 4.6 FACS Sorting

To ensure proper expression of the FUCCI‐3 fluorescent markers in the U2OS‐FUCCI3 cell line or to collect cells according to their cell cycle phase, the cells were incubated for at least 48 h after the final splitting to obtain a homogeneous and asynchronous population. Subsequently, the cells were treated with Trypsin (Thermo Scientific; #25200072), resuspended in DMEM (Gibco; #11966025) supplemented with 10% Fetal Bovine Serum (FBS, Gibco; #10270106) and 1% penicillin/streptomycin (Gibco; #15140122), and placed on ice. The cells were sorted according to their cell cycle stage, as indicated by the FUCCI‐3 fluorescent markers, on a BD Influx Cell Sorter (BD Biosciences) with a stable temperature of 4°C. The 457 nm laser and CFP parameter were used for SLBP‐Turquoise2 detection, the 488 nm laser and FITC parameter were used for Clover‐Geminin detection, and the 561 nm laser and PE parameter were used for Cdt1‐mKO2 detection. For the FUCCI‐3 system's validation, a 355 nm laser was employed to detect Hoechst, and the results were analyzed with Flowjo_V10.

### 4.7 Immunofluorescence

#### 4.7.1 Cell Culture Immunofluorescence

Immunofluorescence experiments were conducted by seeding cells on transparent, flat‐bottom 96‐ or 384‐well plates (Revvity; #6055302, #6057302) and incubating them for a minimum of 48 h prior to fixation. The fixation was conducted by the addition of 16% methanol‐free formaldehyde (Thermo Fisher Scientific; #28908) to the culture, resulting in a final concentration of 4%, for a period of 10 min. The cells were then washed three times with PBS (1x) and permeabilized with 0.2% Triton X‐100 in PBS (1x) for 20 min. The preparation was blocked with 5% bovine serum albumin (BSA) diluted in PBS (1x) for a period of 40 min. Subsequently, the cells were incubated with the primary antibodies for a period of 2.5 h, after which they were washed once with 0.05% Tween 20 in PBS (1x) and twice with PBS (1x). The secondary antibodies were then applied for a period of 45 min, after which the cells were washed once with 0.05% Tween 20 in PBS (1x) and twice with PBS (1x). Furthermore, for U2OS and MCF7 wild‐type cells, a 10‐min incubation with Hoechst 33342 (Thermo Fisher Scientific; #H3570) diluted in PBS (1x) was conducted for DNA staining. All immunofluorescence experiments were performed in parallel with secondary‐antibody–only controls and only antibodies showing a signal above background were included in the analyses.

The following primary antibodies were used: Ki67 (Cell Signaling; #9129; 1:400), p21 (BD Bioscience; #AB_396415; 1:200), H3 (Cell Signaling; #14269), DNMT1 (ProteinTech; #24206‐1‐AP; 1:100), p16 (Cell Signalling; #18769; 1:800), LaminB1 (ProteinTech; #12987‐1‐AP; 1:200), KMT5A (Thermo Fisher Scientific; #PA5‐31467; 1:250), KDM5B (Cell Signaling; #3273; 1:250), PIP5K1A (ProteinTech; #15713‐1‐AP; 1:200), PLCD3 (ProteinTech; #16792‐1‐AP; 1:250), PLD2 (Cell Signaling; #13904; 1:250), Fibrillarin (Cell Signaling; #2639; 1:400), PIP2 (Thermo Fisher Scientific; #MA3‐500; 1:200), HA‐tag (Cell Signalling; #2367; 1:200), H4K20me1 (Diagenode; #C15410034; 1:500), H3K9me3 (Diagenode; #C15410193; 1:1000), and H3K27me3 (Diagenode; #C15410195; 1:200). The following secondary antibodies were used: Alexa Fluor 488 donkey anti‐rabbit (Thermo Fisher Scientific; #A21206; 1:1000), Alexa Fluor 647 goat anti‐rabbit (Thermo Fisher Scientific; #A21244; 1:1000) and Alexa Fluor 633 goat anti‐mouse (Thermo Fisher Scientific; #A21050; 1:1000).

#### 4.7.2 Tissue Micro Array (TMA) Immunofluorescence

Tissue Micro Array From Normal Tissue Were Obtained From TissueArray.Com LLC (#BN1021a). The Fixation Was Conducted by the Addition of 16% Methanol‐free Formaldehyde (Thermo Fisher Scientific; #28908) to the Culture, Resulting in a Final Concentration of 4%, for a Period of 10 min. The Cells Were Then Washed Three Times With PBS (1x) and Permeabilized With 0.2% Triton X‐100 in PBS (1x) for 20 min. The Preparation Was Blocked With 5% Bovine Serum Albumin (BSA) Diluted in PBS (1x) for a Period of 40 min. Subsequently, the Cells Were Incubated With the Primary Antibodies for a Period of 2.5 h, After Which They Were Washed Once With 0.05% Tween 20 in PBS (1x) and Twice With PBS (1x). The Secondary Antibodies Were Then Applied for a Period of 45 min, After Which the Cells Were Washed Once With 0.05% Tween 20 in PBS (1x) and Twice With PBS (1x). Finally, a 10‐min Incubation With Hoechst 33342 (Thermo Fisher Scientific; #H3570) Diluted in PBS (1x) Was Conducted for DNA Staining. The Following Primary Antibodies Were Used: PIP5K1A (ProteinTech; #15713‐1‐AP; 1:200) and PIP2 (Thermo Fisher Scientific; #MA3‐500; 1:200). The Following Secondary Antibodies Were Used: Alexa Fluor 488 Donkey Anti‐rabbit (Thermo Fisher Scientific; #A21206; 1:1000), Alexa Fluor 647 Goat Anti‐rabbit (Thermo Fisher Scientific; #A21244; 1:1000).

### 4.8 Cell Culture High‐Throughput Microscopy

Immunofluorescent images were captured using the Operetta High Content Screening System (PerkinElmer). Cell culture immunofluorescence images were randomly acquired with the 20X air objective in non‐confocal mode, except experiments with PIP2 and Fibrillarin co‐staining, which were acquired with the water 63X in confocal mode. For each experiment, the number of cells analyzed after outlier removal (see *Data analysis and statistics*) is specified in the figure legends. Images were quantified using the Harmony software (version 4.9) and the results were analyzed with the R programming environment (v 4.4).

#### 4.8.1 Cell Cycle Phase Determination

To determine the cell cycle phase based on the FUCCI‐3 system, the excitation filter 390–420 and emission 430–500 was used to measure SLBP‐Turquoise2 signal, the excitation filter 460–490 and emission 500–550 was used to measure Clover‐Geminin signal, and the excitation filter 530–560 and emission 570–650 was used to measure Cdt1‐mKO2 signal. The signal of the 3 colors was merged using Harmony's building block *Calculate Image* and the nuclei were segmented based on this image using the building block *Find Nuclei*. The nuclei crossing the edge of the image were discarded with *Select Population* and the option *Remove Borders Objects*, and their roundness, area, and SER‐Edge were determined with *Calculate Morphology Properties*. The incorrect, low‐quality segmented or unfocused nuclei were visually inspected and removed by applying thresholds considering the roundness, size, and SER‐Edge in the *Select Population* building block. A ring region of 8 pixels was further determined around the segmented nuclei for measuring the background using the building block *Select Cell Region*. The mean intensity for each of the 3 FUCCI fluorescent markers, in the nuclei and their corresponding ring region, was measured with the building block *Calculate Intensity Properties*. The results were exported in a tab separated table, which was used as input for a custom R script to perform further calculations including the compensation for the bleed through signal between Clover and Turquoise2 and subtraction of the background signal (signal from the ring region) from the corresponding nuclei. A scatterplot, named FUCCI‐3 plot, was then generated with the *ggplot2* [84] package by representing the nuclear mean intensity signal of the 3 FUCCI fluorescent markers: log10 Clover‐Geminin in the Y axis, the log10 SLBP‐Turquoise2 in the X axis and the log2 normalized Cdt1‐mKO2 as 3 color scale of the dots. To ensure the visualization of all the dots representing a nucleus, the *geom_voronoi* option from the *ggforce* [85] package was used. Based on the FUCCI‐3 plot pattern, cell cycle gates were drawn. Across different experiments, the shape of the overall pattern varied depending on the specific acquisition conditions. To keep the gating criteria as homogeneous as possible between experiments, the data and the gates were further transformed to show a similar pattern in the control conditions (untreated, DMSO controls or 0 h timepoint). The coordinates from each gate's vertex were used in the *points_in_polygon* tool from the *sp* package [86, 87] to identify in which gate each nucleus was contained, and this was added as a new feature (Phase_Group column) for each nuclei with the *mutate* tool from the *dplyr* [88] package.

To determine the cell cycle phase based on Hoechst staining, we used a strategy adapted from previously published methods [89, 90]. Briefly, the excitation filter 355–385 and emission 430–500 was used to measure Hoechst signal, and the nuclei were segmented based on this image using the building block *Find Nuclei*. The nuclei crossing the edge of the image were discarded with *Select Population* and the option *Remove Borders Objects*, and their roundness and area were determined with *Calculate Morphology Properties*. The incorrect or low‐quality segmented nuclei were visually inspected and removed by applying thresholds considering the roundness and size in the *Select Population* building block. A ring region of 8 pixels was further determined around the segmented nuclei for measuring the background using the building block *Select Cell Region*. The results were exported in a tab separated table, which was used as input for a custom R script to perform further calculations. A histogram was then generated with the *ggplot2* [84] package by representing the nuclear mean intensity signal in the X axis. The histogram was visually inspected to find the signature peaks of G1 and G2 typical and thresholds were determined for the Hoechst signal; these were then used to classify the nuclei into the different cell cycle phases with the *mutate* tool from the *dplyr* [88] package.

#### 4.8.2 Quantification of Nuclear Proteins

The mean intensity, interpreted as the concentration of the targeted protein in the overall nucleus, was calculated using the building block *Calculate Intensity Properties* and selecting the filtered segmented nuclei as the population. The integrated intensity, interpreted as the total amount of protein in the nuclei, was calculated from multiplying the mean intensity times the area of the nucleus in R.

#### 4.8.3 Filtering of Cells Based on PIP5K1A Rescue Construct Localization

For experiments combining endogenous PIP5K1A knockdown with exogenous expression of HA‐tagged PIP5K1A variants (WT, NLS, or NES), cells were pre‐filtered to ensure appropriate subcellular localization of the rescue constructs. Nuclei were segmented based on Hoechst staining using the Harmony building block *Find Nuclei*. Cytoplasmic regions were identified using *Find Cytoplasm*, after which a peri‐nuclear ring region of 9 pixels thickness positioned 3 pixels away from the nuclear boundary was defined. This approach was used to standardize the cytoplasmic sampling region and minimize variability caused by heterogeneous cytoplasmic signal across the cell body. Nuclear and peri‐nuclear cytoplasmic HA signal was quantified from the segmented nuclei and the defined ring region. Cells exhibiting the expected enrichment pattern (predominantly nuclear for NLS constructs, predominantly cytoplasmic for NES constructs) were retained for downstream analyses, while cells displaying inconsistent localization were excluded. This filtering step ensures that only cells expressing the intended construct localization are included in subsequent quantifications.

#### 4.8.4 Data Normalization

To represent the oscillation of protein intensity during the cell cycle in “FUCCI plots”, raw measurements were normalized by dividing the intensity of each nucleus by the population mean, followed by a log2 transformation. The color scale was adjusted to range from ‐1 to 1, emphasizing populations with low and high intensities. For boxplotsand statistical comparisons across treatments and/or cell cycle phases, normalization was performed to account for biological replicates. This was done by extracting each object's intensity value by the population mean (in log2 scale), ensuring data consistency and enabling reliable comparisons across biological replicates.

#### 4.8.5 Data Representation

To represent any feature of interest in the context of cell cycle FUCCI plots were generated. As described before, these plots consist of representing log10 Clover‐Geminin in the Y axis, the log10 SLBP‐Turquoise2 in the X axis and the log2 normalized Cdt1‐mKO2 as 3 color scale of the dots. However, once the cell cycle phases have been visually determined, the color dimension is used for the representation of the feature of interest. These plots were generated with the *ggplot2* package [84] and the *geom_vorono*i option from the *ggforce* package [85]. Boxplots were generated using *geom_boxplot* and each dot representing a nucleus was generated with *geom_sina* from the *ggforce* package [85].

#### 4.8.6 Region Segmentation and Analysis of Foci and Colocalization

PIP2 spots were segmented using Harmony's building block *Find Spots*, using as a population the filtered segmented nuclei and restricting the analysis to this area (other areas like cytoplasm were not considered by the algorithm). Fibrillarin areas were segmented using the building block *Find Region*, selecting the signal above a visually determined threshold with the option *Custom Threshold*. The area of these zones was calculated with *Calculate Morphology Propertie*s and zones with smaller area than 20 pixels were removed from subsequent analysis with *Select Population*. Colocalization of PIP2 spots in Fibrillarin Regions was determined with *Find Position Properties*, selecting the *Cross Population* option. The PIP2 Fibrillarin‐like areas were determined by first identifying the PIP2 spots as previously described. Second, the small and intense spots were selected and their area was extended by 2 pixels. Finally, the touching spots were merged as a single zone and zones bigger than 20 pixels remained segmented. Furthermore, a ring region of 2 pixels thickness was determined with Find Region, selecting as population the PIP2 Fibrillarin‐like areas.

#### 4.8.7 Data Analysis and Statistics

All analyses were carried out in the R programming environment (v 4.4). Statistical parameters, including the exact value of biological replicates (e.g. total number of experiments), deviations, *P*‐values and type of statistical test, are reported in the respective figure legends. Statistical analysis was performed across biological replicates by averaging the respective technical replicates where appropriate. Error bars in graphs represent the mean and standard deviation (SD) of at least three biologically independent experiments. Outliers were defined as single‐cell measurements exceeding ±3 standard deviations from the mean of the corresponding analysis group. The analysis group was defined according to the comparison being performed (e.g., cell‐cycle phase, drug treatment, knockdown condition, or combinations of these factors). Outlier filtering was applied within these groups prior to statistical analysis to avoid bias between experimental conditions. Statistical significance was analyzed using non‐parametric Wilcoxon test with the *wilcox_test* tool from the *rstatix* package [91]. *P* < 0.05 was considered significant.

### 4.9 Tissue Micro Array (TMA) High‐Throughput Microscopy

Immunofluorescent images were captured using the Operetta High Content Screening System (PerkinElmer) with a 40X air objective in confocal mode. Images were collected for DAPI and immunofluorescence channels (546 and 647) using constant acquisition settings across the slide. Image processing and quantification were performed using an in‐house Python pipeline, and downstream analysis and visualization were carried out in the R programming environment (v4.4).

#### 4.9.1 Nuclear Segmentation and Tissue Core Assignment

DAPI signal was acquired using the excitation filter 355–385 and emission filter 430–500. Z‐stacks were selected using the best‐focused plane from an edge‐based sharpness metric computed on the DAPI channel. Nuclei were segmented from the selected DAPI plane using Cellpose (nuclei model). Each acquisition field was mapped to its spatial position using a reconstructed serpentine scan order derived from the filename coordinates (R, C, F). Fields were then assigned to a 10×10 tissue microarray core grid, and core‐level annotations were joined using vendor‐provided metadata for the BN1021a array (TissueArray.Com LLC).

#### 4.9.2 Quantification of Nuclear Proteins

For each segmented nucleus, morphological and per‐channel intensity features were extracted using a custom pipeline. Mean nuclear intensity and integrated intensity (mean × area) were used as measures of relative and total nuclear signal, respectively, with background subtraction performed using field‐level and local ring‐based estimates.

#### 4.9.3 Quality Control and Data Normalization

Morphological features (area, equivalent diameter, eccentricity, perimeter, and solidity) were used for quality control. Morphological outliers were removed using robust univariate Z‐scores and a robust multivariate distance metric. Intensities were log2‐transformed, and nuclei with extreme values were filtered using Z‐score thresholds, with optional per‐channel cutoffs. For cross‐core comparisons, log2 intensities were median‐centered within each core. For cross‐tissue visualization, intensities were Z‐scored per marker to represent deviation from the marker‐specific mean.

#### 4.9.4 Cell Cycle Phase Determination

To determine cell‐cycle phase based on DAPI staining, we used a strategy adapted from previously published methods [89, 90]. A histogram of log2‐transformed nuclear DAPI intensity was generated using the ggplot2 package [84]. A two‐component Gaussian mixture model was fitted to the DAPI intensity distribution using the mclust package to identify the G1 and G2M peaks. Based on the fitted Gaussian means, DAPI intensities were linearly rescaled to DNA‐content units (G1 ≈ 2C and G2M ≈ 4C). Cells with intermediate DNA content (2.5C–3.5C) were assigned to S phase. Cells outside this range were assigned to G1 or G2M based on the posterior probability of belonging to the corresponding Gaussian component (probability ≥ 0.8).

#### 4.9.5 Data Representation

Distributions were visualized using violin plots (geom_violin) overlaid with boxplots (geom_boxplot) from the ggplot2 package [84]. Where indicated, per‐core summary values were displayed as points using geom_point.

#### 4.9.6 Data Analysis and Statistics

All analyses were carried out in the R programming environment (v4.4). Statistical parameters, including the exact number of biological replicates, P‐values, and statistical tests, are reported in the respective figure legends. Where applicable, technical replicates were averaged prior to statistical testing. Error bars represent mean ± SD of at least three biologically independent experiments. Statistical significance for comparisons across cell‐cycle phases was assessed by one‐way ANOVA followed by Tukey's HSD post hoc test using the anova_test and tukey_hsd functions from the rstatix package [91]. Correlations between different markers were calculated using Pearson correlation (cor and cor.test functions from the stats package [92]). P < 0.05 was considered statistically significant.

### 4.10 Live Cell Imaging

Cells were seeded in transparent, flat‐bottom 96‐ or 384‐well plates (Revvity; #6055302, #6057302) and allowed to incubate for at least 18 h before starting the acquisition. For experiments involving DMSO or drug treatments, the compounds were added immediately prior to the start of data acquisition. Images were captured using the Operetta High Content Screening System (PerkinElmer) with the 20X air objective in non‐confocal. Images were quantified using the Harmony software (version 4.9) and the results analyzed with R programming environment (v 4.4).

#### 4.10.1 Tracking

Individual U2OS FUCCI‐3 nuclei were first segmented as described in the *Cell culture High‐Throughput Microscopy*—*Cell cycle phase determination* section and subsequently tracked over time using Harmony's *Track Cells* building block. Cell cycle phase was then assigned to each tracked nucleus at each time point as detailed in the same section. Only tracks spanning at least 20 time points and initiating in G1 or G0 were retained for downstream analyses.

### 4.11 Drug Treatments

Cells were cultured for 24 h before being treated with DMSO (PanReac AppliChem; #A3672) or specific compounds, including Nocodazole (MedChem Express; #HY‐13520), RO‐3306 (MedChem Express; #HY‐12529), and ISA‐2011B (MedChem Express; #HY‐16937), for the durations and concentrations specified in the manuscript. DMSO controls were prepared using the same volume and dilution as the corresponding drug treatments in each experiment.

### 4.12 Chromatome

One million cells were initially lysed in 0.5% CHAPS (3‐cholamidopropyl dimethylammonio 1‐propanesulfonate) (Roche; #10810118001) in PBS (1x) for a period of 20 min, with the objective of disrupting the cytosolic membrane. Following this, the lysate was subjected to centrifugation for a duration of 5 min at 720 g at a temperature of 4°C. The supernatant was harvested as a cytosolic fraction, while the nuclear pellet was resuspended in Cytoplasmic Lysis Buffer (comprising IGEPAL 0.1%, NaCl 150 mM, Tris‐HCl 10 mM, pH 7, in H_2_O). The sorted cells from each phase, with the exception of the mitotic phase, were placed on top of a sucrose gradient buffer (NaCl 150 mM, sucrose 25%, Tris‐HCl 10 mM pH 7 in H2O) and subjected to centrifugation for a period of 5 min at a speed of 1200 g at a temperature of 4°C. The purified nuclei or lysed mitotic cells were then washed three times by resuspension in Nuclei Washing Buffer (EDTA 1 mM, IGEPAL 0.1% in PBS) and centrifugation for 5 min at 1200 g at 4°C. Subsequently, the pellet was resuspended in Nuclei Resuspension Buffer (EDTA 1 mM, NaCl 75 mM, 50% sucrose, Tris‐HCl 20 mM pH 8 in H2O), and the nuclear membrane was lysed by adding Nuclei Lysis Buffer (EDTA 0.2 mM, HEPES 20 mM pH 7.5, IGEPAL 0.1%, NaCl 300 mM in H2O), vortexing, and incubating for 5 min. Following a 2‐min centrifugation at 16,000 rpm at 4°C, the supernatant was harvested as the nucleoplasmic fraction, while the resulting chromatin pellet was resuspended in Benzonase Digestion Buffer (15 mM HEPES pH 7. The chromatin pellet was resuspended in 0.1% IGEPAL, 5 mg/mL TPCK, and sonicated on a Bioruptor Pico (Diagenode) for 15 cycles of 30 s on and 30 s off in 1.5 mL Diagenode tubes (Diagenode; #C30010016). Subsequently, the sonicated chromatin was digested with Benzonase enzyme (VWR; #706643; 2.5U) for 30 min at room temperature. The resulting sample was harvested as a Chromatome fraction. Unless otherwise stated, all steps were conducted on ice, and all buffers were supplemented with proteinase inhibitors (Roche; #4693132001). The concentrations of the cytosolic and chromatome extracts were determined using the Pierce BCA Protein Assay Kit (Thermo Scientific; #PIER23225).

### 4.13 Western Blot

The samples were combined with 4X Laemmli sample buffer (Bio‐Rad; #1610747) and heated to 95°C for 3 min. The proteins were separated by sodium dodecyl sulphate–polyacrylamide gel electrophoresis and transferred to a nitrocellulose membrane by wet transfer (25 mM Tris‐Base, 192 mM Glycine, 20% methanol in H_2_O). The membranes were then blocked for a minimum of 15 min at room temperature in 5% skim milk in 0.05% Tween 20 in PBS (1x). The membranes were incubated with the primary antibodies diluted in 0.05% Tween 20 in PBS (1x), for either 16 h at 4°C or for 1 h at room temperature. The membranes were washed 3 times with 0.05% Tween 20 in PBS (1x), the secondary antibodies, diluted in 0.05% Tween 20 in PBS (1x), were then applied for a period of 1 h at room temperature. Finally, the membrane was washed 3 times with 0.05% Tween 20 in PBS (1x). The detection of the protein was done using the Odyssey CLx (Li‐Cor) system, and the resulting data were analyzed using Image Studio Lite (version 5.2.5).

The following primary antibodies were used: Vinculin (Cell Signaling; #13901; 1:1000), H3 (Cell Signaling; #14269; 1:1000), H3S10ph (Merck; #06‐570; 1:2000). The following secondary antibodies were used: Alexa Fluor 800 donkey anti‐rabbit (Thermo Fisher Scientific; #A32735; 1:10000) and Alexa Fluor 680 goat anti‐mouse (Thermo Fisher Scientific; #A21244; 1:10000).

### 4.14 Mass Spectrometry

#### 4.14.1 Liquid Chromatography Coupled to Tandem Mass Spectrometry (LC‐MS/MS)

The protein concentrations from chromatin enriched samples were determined using the BCA protein assay kit (Applichem CmBH, Darmstadt,Germany), and 10 µg per sample was processed using an adapted Single‐Pot solid‐phase‐enhanced sample preparation (SP3) methodology [93]. Briefly, equal volumes (125 µL containing 6250 µg) of two different kind of paramagnetic carboxylate modified particles (SpeedBeads 45152105050250 and 65152105050250; GE Healthcare) were mixed, washed three times with 250 µL water and reconstituted to a final concentration of 50 µg/µL with LC‐MS grade water (LiChrosolv; MERCK KgaA). Samples were filled up to 100 µL with stock solutions to reach a final concentration of 2% SDS, 100 mM HEPES, pH 8.0, and proteins were reduced by incubation with a final concentration of 10 mM DTT for 1 h at 56°C. After cooling down to room temperature, reduced cysteines were alkylated with iodoacetamide at a final concentration of 55 mM for 30 min in the dark. For tryptic digestion, 400 µg of mixed beads were added to reduced and alkylated samples, vortexed gently and incubated for 5 min at room temperature. The formed particles‐protein complexes were precipitated by addition of acetonitrile to a final concentration of 70% [V/V], mixed briefly via pipetting before incubating for 18 min at room temperature. Particles were then immobilized using a magnetic rack (DynaMag‐2 Magnet; Thermo Fisher Scientific) and supernatant was discarded. SDS was removed by washing two times with 200 µL 70% ethanol and one time with 180 µL 100% acetonitrile. After removal of organic solvent, particles were resuspended in 100 µL of 50 mM NH4HCO3 and samples digested by incubating with 1 µg of Trypsin overnight at 37°C. Samples were acidified to a final concentration of 1% Trifluoroacetic acid (Uvasol; MERCK KgaA) prior to immobilizing the beads on the magnetic rack. Peptides were desalted using C18 solid phase extraction spin columns (Pierce Biotechnology, Rockford, IL). Finally, eluates were dried in a vacuum concentrator and reconstituted in 10 µL of 0.1% TFA.

Mass spectrometry was performed on an Orbitrap Fusion Lumos mass spectrometer (ThermoFisher Scientific, San Jose, CA) coupled to an Dionex Ultimate 3000RSLC nano system (ThermoFisher Scientific, San Jose, CA) via nanoflex source interface. Tryptic peptides were loaded onto a trap column (Pepmap 100 5 µm, 5 × 0.3 mm, ThermoFisher Scientific, San Jose, CA) at a flow rate of 10 µL/min using 0.1% TFA as loading buffer. After loading, the trap column was switched in‐line with a 50 cm, 75 µm inner diameter analytical column (packed in‐house with ReproSil‐Pur 120 C18‐AQ, 3 µm, Dr. Maisch, Ammerbuch‐Entringen, Germany). Mobile‐phase A consisted of 0.4% formic acid in water and mobile‐phase B of 0.4% formic acid in a mix of 90% acetonitrile and 10% water. The flow rate was set to 230 nL/min and a 90 min gradient used (4% to 24% solvent B within 82 min, 24% to 36% solvent B within 8 min and, 36% to 100% solvent B within 1 min, 100% solvent B for 6 min before bringing back solvent B at 4% within 1 min and equilibrating for 18 min).

Analysis was performed in a data‐independent acquisition (DIA) mode. Full MS scans were acquired with a mass range of 375 – 1250 m/z in the orbitrap, an RF lens set at 40%, and at a resolution of 120,000 (at 200 m/z). The automatic gain control (AGC) was set to a target of 4 × 105, and a maximum injection time of 54 ms was applied, scanning data in profile mode. MS1 scans were followed by 41 MS2 customed windows. The MS2 scans were acquired in the Orbitrap at a resolution of 30,000 (at 200 m/z), with an AGC set to target 2 × 105, for a maximum injection time of 54 ms. Fragmentation was achieved with higher energy collision induced dissociation (HCD) at a fixed normalized collision energy (NCE) of 35%. A single lock mass at m/z 445.120024 was employed [94]. Xcalibur version 4.3.73.11 and Tune 3.4.3072.18 were used to operate the instrument.

The mass spectrometry data has been deposited to the ProteomeXchange Consortium via the PRIDE partner repository [95] with the dataset identifier PXD055357.

#### 4.14.2 Data Analysis

Chromatin Data Were Analyzed Using DIA‐NN Software [96] Which Were Subsequently Normalized Using the *Normalize_Vsn* and *Median_Normalisation* Functions From the *DEP* [97] and *proDA* [98] Packages, Respectively [92]. The Rest of the Pipeline Was Followed According to the *DEP* Package, With the Inclusion of *Impute.Mi* Function for Protein‐imputation From the *imp4p* Package [99]. Relative Enrichment of each Sample Was Estimated as the Relative Abundances of Known Histone Proteins in each Sample [100] and all Biological Enrichment Performed Using the *clusterProfiler* Tool [101], and Regression Correction Was Performed Using the *Rlm* Function [102]. Significantly Change Their Levels of Chromatin, Between at Least Two Consecutive Phases, Was Performed Using the CORREP R Package [103]. The Profiles Were Sorted According to Their Similarities by Calculating a Distance Matrix With the Tool *Dist*, With the *Euclidean* Method, Followed by Hierarchical Clustering With the Tool *Hclust* With the *Ward.D2* Method, and Further Grouped in 6 Clusters With the Function *Cutree*; these Functions Belong to the *Stats* Package [92]. Known Subcellular Localisations for Proteins Were Obtained From the *pRoloc* R Package, hyperLOPITU2OS2018 [104]. Analysis Was Facilitated by the Tidyverse [105] and Data Table [105] Collection of Packages. Essentiality and Drug Sensitivity Data Were Conducted by Comparing Chromatin Abundances to Gene Essentialities and Drug Sensitivities [106]. Prediction of Nuclear Localisation Signals Was Performed on the Input Fasta File Using PredictNLS [107]. Evolutionary Ages of Proteins Were Derived From Gene‐Ages [108]. Interactors and Sub Cellular Localization Were Obtained From OpenCell [52].

## Author Contributions

A.G.Z.: Conceptualization; formal analysis; validation; investigation; visualization; methodology; writing – original draft. S.K.: Data curation; formal analysis; investigation; writing – original draft. C.R.E.: Data curation; formal analysis; writing – review & editing. M.L.E.C.: Investigation; writing – original draft. C.T.M.: Data curation; formal análisis. A.C.M.: Investigation. A.S.: Investigation. L.G.‐L.: Investigation. L.W.: Investigation. M.G.: investigation. F.F.: Data curation; formal analysis; investigation. A.M.: Supervision. A.C.M.: Data curation; formal analysis; investigation; methodology. S.S.: Conceptualization; supervision; funding acquisition; visualization; methodology; writing – original draft; project administration.

## Conflicts of Interest

The authors declare no conflicts of interest.

## Supporting information




**Supporting File 1**: advs75068‐sup‐0001‐SuppMat.docx.

**Supporting File 2**: advs75068‐sup‐0002‐FigureS1‐S15.pdf.

## Data Availability

The raw mass spectrometry proteomics data have been deposited to the PRIDE repository [95] under accession PXD055357. Analysis scripts used for figure generation are available at Github (https://github.com/SdelciLab/Cell_Cycle_Chromatome). Processed data and the corresponding code have been deposited in Zenodo (DOI reserved: 10.5281/zenodo.18605830) and will be made publicly available upon acceptance.
