# Supplementary material for: Cell Cycle Control of Nuclear Metabolism Couples Phosphatidylinositol Signaling to Histone Methylation
Source: Adv Sci (Weinh). 2026 Apr 21;13(34):e01083. doi: 10.1002/advs.202501083 (PMC13285136; doi:10.1002/advs.202501083)
Supplement: Supplementary file 1 — Supporting File 1: advs75068‐sup‐0001‐SuppMat.docx. [file ADVS-13-e01083-s002.docx]

## Supplementary Figures

Supplementary Figure 1. Validation of the FUCCI-3 U2OS reporter cell line. (A) Number of U2OS FUCCI-3 and FUCCI-4 (FUCCI-3 + H1-Maroon - H1M -) cells in G1, S and G2/M determined by integration of the FUCCI fluorescent markers measured by FACS. At least 3 biological replicates were analyzed per cell line. In each of the conditions, at least 47489 cells were considered and statistical analysis was performed using a t-test. (B) The illustration depicts the pipeline for the analysis of high-throughput microscopy images in the context of the cell cycle. The images of the FUCCI-3 fluorescent markers were acquired and subsequently merged, thereby facilitating the identification of all nuclei. A ring of eight pixels was drawn around the region of interest to measure the perinuclear intensity of the FUCCI fluorescent markers and the resulting value was used as background reference. The background was subtracted from the mean intensity of the nuclei, thereby enabling the determination of the corrected level of each of the FUCCI fluorescent markers within the nucleus. The specific combinations of the corrected expression levels of these fluorescent markers facilitated the identification of the cell cycle phase. For the purposes of analysis, a gating procedure was performed using an XYZ projection (x-axis = SLBP-Turquoise2, y-axis = Clover-Geminin, z-axis plotted as a color gradient = Cdt1-mKO2), resulting in the FUCCI-3 plot representations, which will henceforth be referred to as such. (C) The three FUCCI-3 plots here represent the cell cycle integrity of the U2OS cell population fixed and stained for either Ki27, p21 or H3. (D) Immunofluorescence-based quantification of nuclear areas across cell cycle phases determined with the FUCCI-3 system of the U2OS cell population fixed and stained for H3. 3 biological replicates were analyzed (*n*G0 = 1436, *n*G1 = 2838, *n*S = 1176, *n*G2/M = 638, *n*M = 104; outliers removed, 3 SD; unpaired two-tailed Wilcoxon test). (E) Immunofluorescence-based quantification of the integrated intensity of Ki67, p21 and H3 across cell cycle phases determined with the FUCCI-3 system. 3 biological replicates were analyzed (Ki67 IF: *n*G0 = 1187, *n*G1 = 3055, *n*S = 1130, *n*G2/M = 527, *n*M = 102; p21 IF: *n*G0 = 1369, *n*G1 = 2775, *n*S = 1138, *n*G2/M = 631, *n*M = 126; H3 IF: *n*G0 = 1433, *n*G1 = 2844, *n*S = 1173, *n*G2/M = 640, *n*M = 102; outliers removed, 3 SD; unpaired two-tailed Wilcoxon test). (F) FUCCI-3 plots representing the cell cycle integrity of the U2OS cell population fixed and stained for laminB1 quantification and representation of laminB1 nuclear intensity measured by immunofluorescence and plotted according to FUCCI-3-determined cell cycle phases. (G) Immunofluorescence-based quantification of the mean intensity of laminB1 in the nuclear region identified as the combined signal from the FUCCI-3 fluorescent markers, which has been used for the determination of cell cycle phases. 3 biological replicates were analyzed (*n*G0 = 1401, *n*G1 = 2600, *n*S = 1186, *n*G2/M = 674, *n*M = 119; outliers removed, 3 SD; unpaired two-tailed Wilcoxon test). (H) Representative images showing the changes of laminB1 in the nucleus during cell cycle progression, as well as the respective signal of the FUCCI-3 fluorescent markers of the illustrated cell, which confirm the belonging to the assigned cell cycle phase. Scale bar is 20µm. (I) FUCCI-3 plots representing the cell cycle integrity of the U2OS cell population fixed and stained for p16 quantification and representation of p16 nuclear intensity measured by immunofluorescence and plotted according to FUCCI-3-determined cell cycle phases. (J) Immunofluorescence-based quantification of the mean intensity of p16 in the nuclear region identified as the combined signal from the FUCCI-3 fluorescent markers, which has been used for the determination of cell cycle phases. 3 biological replicates were analyzed (*n*G0 = 1383, *n*G1 = 2955, *n*S = 1242, *n*G2/M = 661, *n*M = 110; outliers removed, 3 SD; unpaired two-tailed Wilcoxon test).

Supplementary Figure 2. Validation of the FUCCI-3 MCF7 reporter cell line. (A) FUCCI-3 plots representing the cell cycle integrity of the MCF7 FUCCI-3 cell population fixed and stained for ki67 quantification. (B) Representation of ki67 nuclear mean intensity measured by immunofluorescence and plotted according to FUCCI-3-determined cell cycle phases. (C) Immunofluorescence-based quantification of the mean intensity of ki67 in the nuclear region identified as the combined signal from the FUCCI-3 fluorescent markers, which has been used for the determination of cell cycle phases. 3 biological replicates were analyzed (*n*G0 = 2309, *n*G1 = 7137, *n*S = 3229, *n*G2/M = 1598, *n*M = 646; outliers removed, 3 SD; unpaired two-tailed Wilcoxon test). (D) FUCCI-3 plots representing the cell cycle integrity of the MCF7 FUCCI-3 cell population fixed and stained for p21 quantification. (E) Representation of p21 nuclear mean intensity measured by immunofluorescence and plotted according to FUCCI-3-determined cell cycle phases. (F) Immunofluorescence-based quantification of the mean intensity of p21 in the nuclear region identified as the combined signal from the FUCCI-3 fluorescent markers, which has been used for the determination of cell cycle phases. 3 biological replicates were analyzed (*n*G0 = 2394, *n*G1 = 7046, *n*S = 3465, *n*G2/M = 1646, *n*M = 695; outliers removed, 3 SD; unpaired two-tailed Wilcoxon test). (G) FUCCI-3 plots representing the cell cycle integrity of the MCF7 FUCCI-3 cell population fixed and stained for H3 quantification. (H) Representation of H3 nuclear mean intensity measured by immunofluorescence and plotted according to FUCCI-3-determined cell cycle phases. (I) Immunofluorescence-based quantification of the mean intensity of H3 in the nuclear region identified as the combined signal from the FUCCI-3 fluorescent markers, which has been used for the determination of cell cycle phases. 3 biological replicates were analyzed (*n*G0 = 2470, *n*G1 = 7626, *n*S = 3845, *n*G2/M = 1945, *n*M = 711; outliers removed, 3 SD; unpaired two-tailed Wilcoxon test). (J) FUCCI-3 plots representing the cell cycle integrity of the MCF7 FUCCI-3 cell population fixed and stained for laminB1 quantification. (K) Representation of laminB1 nuclear mean intensity measured by immunofluorescence and plotted according to FUCCI-3-determined cell cycle phases. (L) Immunofluorescence-based quantification of the mean intensity of laminB1 in the nuclear region identified as the combined signal from the FUCCI-3 fluorescent markers, which has been used for the determination of cell cycle phases. 3 biological replicates were analyzed (*n*G0 = 2772, *n*G1 = 7768, *n*S = 3872, *n*G2/M = 2098, *n*M = 738; outliers removed, 3 SD; unpaired two-tailed Wilcoxon test). (M) FUCCI-3 plots representing the cell cycle integrity of the MCF7 FUCCI-3 cell population fixed and stained for p16 quantification. (N) Representation of p16 nuclear mean intensity measured by immunofluorescence and plotted according to FUCCI-3-determined cell cycle phases. (O) Immunofluorescence-based quantification of the mean intensity of p16 in the nuclear region identified as the combined signal from the FUCCI-3 fluorescent markers, which has been used for the determination of cell cycle phases. 3 biological replicates were analyzed (*n*G0 = 2127, *n*G1 = 6519, *n*S = 3461, *n*G2/M = 1674, *n*M = 678; outliers removed, 3 SD; unpaired two-tailed Wilcoxon test).

Supplementary Figure 3. FUCCI-3 U2OS reporter cell line live cell validation. (A) Growth curve of U2OS cells treated with DMSO (negative control), RO-3306 (4.5 μM; 60 hours) or Nocodazole (0.5 μM; 60 hours). FUCCI-3 U2OS cells were monitored for 60 hours. Nuclei were identified by the expression of FUCCI-3 fluorescent markers and counted over time to determine cell growth. Tracking was performed with a minimum of 200 cells per treatment. (B) Live cell imaging of cells treated with RO-3306 (4.5 μM) or Nocodazole (0.5 μM) and monitored for 60 hours. Changes in the number of Clover-Geminin negative (G1) and positive (S & G2) cells are shown in the ridge plot, illustrating the cell cycle distribution from the beginning of the treatment and every 6 hours. (C) FUCCI-3 plots showing cell cycle changes after treatment with DMSO, RO-3306 (4.5 μM) or Nocodazole (0.5 μM) for 18h. (D) FUCCI-3 plots timepoints of tracked cells starting the live cell imaging at either G1 or G0 phases. 3 biological replicates were analyzed. (E) Stacked bars representing the percentage of cells in each phase during a 60h timelapse, for cells starting at G1 or G0 phases. Tracked which last timepoint fell in G2M or M gates were considered as “Divided”, or “Lost” otherwise, for the rest of the timepoints. (F) Illustration of the gating strategy based on the FUCCI-3 plot representation used to sort the displayed populations. (G) Live cell imaging U2OS FUCCI-3 at time points 0h, 20h and 40h after sorting, showing the progression of the cell cycle based on the synchronous oscillation of the FUCCI-3 colors. Scale bar is 50µm. (H) Visual illustration of the in-house chromatome protocol showing how the different subcellular fractions are obtained. (I) Western blot validation of the cytoplasmic fraction using Vinculin as a cytoplasmic marker and H3 as a chromatin marker.

**Supplementary Figure 4. Curation and validation of cell cycle chromatome data.** (A) Mass spectrometry data distribution pre (raw data) and post (normalized data) normalization. Normalization of input levels of sample material was performed using the *vsn* and *median_normalisation* functions of the DEP R package. (B) PCA plots (1 vs 2 and 3 vs 4) of mass spectrometry data of chromatome cell cycle samples and chromatomes from unsorted/unsynchronized cells. PCA plots were generated using the DEP R package^112^ excluding any proteins which contained missing values. (C) Enrichment of known proteins to be present at different subcellular compartments in the different cell cycle phases was based on the U2OS hyperLOPIT annotations. (D) Clustering of proteins found to significantly change their levels of chromatin, between at least two consecutive phases, was performed using the CORREP R package. (E) Clustered heatmap of proteins significantly changing chromatin abundance across phases. (F) Significantly enriched GeneOntology terms the different clusters, against a background of all chromatin detected proteins.

**Supplementary Figure 5. Metabolic enzymes oscillating on chromatin during the cell cycle in U2OS FUCCI-3 cell line.** (A) Relative chromatin abundance of metabolism-related proteins found in association with chromatin in a cell cycle dependent manner in our mass spectrometry analysis of phase-specific chromatin fractions. Magenta lines mean that the difference between the 2 consecutive phases was significant. Black lines mean that no significant difference was found between consecutive phases. Semi-transparent dots mean that the value was imputed. Statistical analysis was performed as in Figure S4B. The proteins were sorted using a hierarchical clustering algorithm based on similarities of protein abundance on chromatin across the cell cycle, and grouped in 6 different clusters, shown here in different colors. (B) FUCCI-3 plots showing the integrity of the cell cycle in the cell population used for the immunofluorescence detection of DNMT1, (C) KMT5A and (D) KDM5B. (E) Immunofluorescence-based quantification of the integrated intensity of DNMT1, (F) KMT5A and (G) KDM5B across cell cycle phases determined with the FUCCI-3 system. (E-G) Immunofluorescence-based quantification of the integrated intensity of (E) DNMT1, (F) KMT5A and (G) KDM5B across cell cycle phases determined with the FUCCI-3 system. 3 biological replicates were analyzed (DNMT1 IF: *n*G0 = 1308, *n*G1 = 2793, *n*S = 1068, *n*G2/M = 538, *n*M = 96; KMT5A IF: *n*G0 = 1182, *n*G1 = 2841, *n*S = 937, *n*G2/M = 561, *n*M = 101; KDM5B IF: *n*G0 = 1257, *n*G1 = 3408, *n*S = 1192, *n*G2/M = 581, *n*M = 117; outliers removed, 3 SD; unpaired two-tailed Wilcoxon test).

**Supplementary Figure 6. Metabolic enzymes oscillating on chromatin during the cell cycle in MCF7 FUCCI-3 cell line.** (A-C) FUCCI-3 plots showing the integrity of the cell cycle in the cell population used for the immunofluorescence detection of (A) DNMT1, (B) KMT5A and (C) KDM5B. (D-F) Representation of (D) DNMT1, (E) KMT5A or (F) KDM5B nuclear intensity measured by immunofluorescence and plotted according to FUCCI-3-determined cell cycle phases. (G-I) Immunofluorescence-based quantification of nuclear (G) DNMT1, (H) KMT5A or (I) KDM5B mean intensities across cell cycle phases determined with the FUCCI-3 system. 3 biological replicates were analyzed (DNMT1 IF: *n*G0 = 2767, *n*G1 = 7899, *n*S = 4027, *n*G2/M = 2126, *n*M = 751; KMT5A IF: *n*G0 = 2445, *n*G1 = 7223, *n*S = 3730, *n*G2/M = 1927, *n*M = 753; KDM5B IF: *n*G0 = 2363, *n*G1 = 6459, *n*S = 3464, *n*G2/M = 1882, *n*M = 683; outliers removed, 3 SD; unpaired two-tailed Wilcoxon test). (J-L) Immunofluorescence-based quantification of nuclear (J) DNMT1, (K) KMT5A or (L) KDM5B mean intensities across cell cycle phases determined with the FUCCI-3 system. 3 biological replicates were analyzed (DNMT1 IF: *n*G0 = 2756, *n*G1 = 7875, *n*S = 4015, *n*G2/M = 2108, *n*M = 747; KMT5A IF: *n*G0 = 2447, *n*G1 = 7204, *n*S = 3712, *n*G2/M = 1910, *n*M = 752; KDM5B IF: *n*G0 = 2382, *n*G1 = 6457, *n*S = 3451, *n*G2/M = 1861, *n*M = 686; outliers removed, 3 SD; unpaired two-tailed Wilcoxon test).**Supplementary Figure 7.** **Analysis of PIP2 metabolism in the nucleus of U2OS FUCCI-3 cell line.** (A) Circus plot representing the interactions between chromatome-MS identified PIP2 metabolism proteins PIP5K1A, PIP4K2C, PLCD3 and PLD2 with known nuclear or cytoplasmatic localized proteins extracted from the OpenCell database. Bar plots represent the median abundance in each cell cycle phase found in our chromatome-MS data. (B) FUCCI-3 plot representing the cell cycle integrity of the cell population fixed and stained for PIP5K1A quantification. (C) Representation of PIP5K1A nuclear intensity measured by immunofluorescence and plotted according to FUCCI-3-determined cell cycle phases. (D) Immunofluorescence-based quantification of the integrated intensity of PIP5K1A across cell cycle phases determined with the FUCCI-3 system. 3 biological replicates were analyzed (*n*G0 = 1267, *n*G1 = 3146, *n*S = 1131, *n*G2/M = 548, *n*M = 122; outliers removed, 3 SD; unpaired two-tailed Wilcoxon test). (E) FUCCI-3 plot representing the cell cycle integrity of the cell population fixed and stained for PLCD3 quantification. (F) Representation of PLCD3 nuclear intensity measured by immunofluorescence and plotted accordingly to FUCCI-3-determined cell cycle phases. (G) Immunofluorescence-based quantification of the integrated intensity of PLCD3 across cell cycle phases determined with the FUCCI-3 system. 3 biological replicates were analyzed (*n*G0 = 1352, *n*G1 = 3060, *n*S = 1140, *n*G2/M = 558, *n*M = 105; outliers removed, 3 SD; unpaired two-tailed Wilcoxon test). (H) FUCCI-3 plot representing the cell cycle integrity of the cell population fixed and stained for PLD2 quantification. (I) Representation of PLD2 nuclear intensity measured by immunofluorescence and plotted accordingly to FUCCI-3-determined cell cycle phases. (J) Immunofluorescence-based quantification of the integrated intensity of PLD2 across cell cycle phases determined with the FUCCI-3 system. 3 biological replicates were analyzed (*n*G0 = 1173, *n*G1 = 3290, *n*S = 1252, *n*G2/M = 570, *n*M = 128; outliers removed, 3 SD; unpaired two-tailed Wilcoxon test). (K) FUCCI-3 plot representing the cell cycle integrity of the cell population fixed and stained for PIP2 quantification. (L) Representation of PIP2 nuclear intensity measured by immunofluorescence and plotted according to FUCCI-3-determined cell cycle phases. (M) Immunofluorescence-based quantification of the integrated intensity of PIP2 across cell cycle phases determined with the FUCCI-3 system. 3 biological replicates were analyzed (*n*G0 = 1417, *n*G1 = 2953, *n*S = 1226, *n*G2/M = 639, *n*M = 101; outliers removed, 3 SD; unpaired two-tailed Wilcoxon test).

**Supplementary Figure 8.** **Analysis of PIP2 metabolism in the nucleus of MCF7 FUCCI-3 cell line.** (A) FUCCI-3 plot representing the cell cycle integrity of the cell population fixed and stained for PIP5K1A quantification. (B) Representation of PIP5K1A nuclear intensity measured by immunofluorescence and plotted according to FUCCI-3-determined cell cycle phases. (C) Immunofluorescence-based quantification of the mean intensity of PIP5K1A across cell cycle phases determined with the FUCCI-3 system. 3 biological replicates were analyzed (*n*G0 = 2448, *n*G1 = 7341, *n*S = 3819, *n*G2/M = 1986, *n*M = 715; outliers removed, 3 SD; unpaired two-tailed Wilcoxon test). (D) Immunofluorescence-based quantification of the integrated intensity of PIP5K1A across cell cycle phases determined with the FUCCI-3 system. 3 biological replicates were analyzed (*n*G0 = 2449, *n*G1 = 7285, *n*S = 3793, *n*G2/M = 1967, *n*M = 711; outliers removed, 3 SD; unpaired two-tailed Wilcoxon test). (E) FUCCI-3 plot representing the cell cycle integrity of the cell population fixed and stained for PLCD3 quantification. (F) Representation of PLCD3 nuclear intensity measured by immunofluorescence and plotted according to FUCCI-3-determined cell cycle phases. (G) Immunofluorescence-based quantification of the mean intensity of PLCD3 across cell cycle phases determined with the FUCCI-3 system. 3 biological replicates were analyzed (*n*G0 = 2316, *n*G1 = 6939, *n*S = 3372, *n*G2/M = 1844, *n*M = 736; outliers removed, 3 SD; unpaired two-tailed Wilcoxon test). (H) Immunofluorescence-based quantification of the integrated intensity of PLCD3 across cell cycle phases determined with the FUCCI-3 system. 3 biological replicates were analyzed (*n*G0 = 2308, *n*G1 = 6901, *n*S = 3351, *n*G2/M = 1833, *n*M = 731; outliers removed, 3 SD; unpaired two-tailed Wilcoxon test). (I) FUCCI-3 plot representing the cell cycle integrity of the cell population fixed and stained for PLD2 quantification. (J) Representation of PLD2 nuclear intensity measured by immunofluorescence and plotted according to FUCCI-3-determined cell cycle phases. (K) Immunofluorescence-based quantification of the mean intensity of PLD2 across cell cycle phases determined with the FUCCI-3 system. 3 biological replicates were analyzed (*n*G0 = 2565, *n*G1 = 7506, *n*S = 4048, *n*G2/M = 2145, *n*M = 724; outliers removed, 3 SD; unpaired two-tailed Wilcoxon test). (L) Immunofluorescence-based quantification of the integrated intensity of PLD2 across cell cycle phases determined with the FUCCI-3 system. 3 biological replicates were analyzed (*n*G0 = 2571, *n*G1 = 7492, *n*S = 4030, *n*G2/M = 2139, *n*M = 721; outliers removed, 3 SD; unpaired two-tailed Wilcoxon test). (M) FUCCI-3 plot representing the cell cycle integrity of the cell population fixed and stained for PIP2 quantification. (N) Representation of PIP2 nuclear intensity measured by immunofluorescence and plotted according to FUCCI-3-determined cell cycle phases. (O) Immunofluorescence-based quantification of the mean intensity of PIP2 across cell cycle phases determined with the FUCCI-3 system. 3 biological replicates were analyzed (*n*G0 = 2512, *n*G1 = 7342, *n*S = 3710, *n*G2/M = 1966, *n*M = 782; outliers removed, 3 SD; unpaired two-tailed Wilcoxon test). (P) Immunofluorescence-based quantification of the integrated intensity of PIP2 across cell cycle phases determined with the FUCCI-3 system. 3 biological replicates were analyzed (*n*G0 = 2507, *n*G1 = 7342, *n*S = 3706, *n*G2/M = 1961, *n*M = 784; outliers removed, 3 SD; unpaired two-tailed Wilcoxon test).

**Supplementary Figure 9.** **Analysis of PIP2 metabolism healthy tissues.** (A) Computational reconstruction of healthy tissue TMA based on stitched nuclear coordinates, showing spatial distribution of segmented nuclei from DAPI-stained sections across tissue cores. (B) Density plots of morphological parameters used to homogenize the segmented nuclei population across all analyzed tissues. Top panels show before (orange) and after (blue) filtering populations; bottom panels show the post-filtering population only (blue). (C) Density plots of log2 mean intensity and log2 integrated intensity for DAPI, PIP2, and PIP5K1A in healthy tissues, showing before (orange) and after (blue) filtering populations. (D) Immunofluorescence-based quantification of nuclear PIP2 and PIP5K1A log2 integrated intensity in TMAs of healthy tissues. Three biological replicates were analyzed; data are shown as distributions with per-core means. (E) Density plots of normalized DAPI intensity from the QC-filtered (homogenized) segmented nuclei population across healthy tissues. Each shade represents a different tissue section. (F) Histogram of normalized DAPI intensity from healthy liver tissue containing proliferating cells. Density lines indicate Gaussian fits used to identify peaks corresponding to G1 (orange) and G2M (green) populations.
(G) Histogram of normalized DAPI intensity from healthy liver tissue containing proliferating cells, with intervals corresponding to G1 (orange), S (blue), and G2M (green) populations.

**Supplementary Figure 10.** **PIP2 and nucleoli variation across cell cycle in U2OS and MCF7 cell lines.** (A) Example of Fibrillarin segmentation (shown in green) and the colocalization of PIP2 foci (shown in red) inside Fibrillarin regions on confocal images. The nucleus is shown in blue via Hoechst staining; scale bar is 10µm. (B) Immunofluorescence-based quantification of the nucleolar area across cell cycle phases determined with the FUCCI-3 system. 3 biological replicates were analyzed (*n*G0 = 1310, *n*G1 = 3073, *n*S = 1162, *n*G2/M = 594, *n*M = 140; outliers removed, 3 SD; unpaired two-tailed Wilcoxon test). (C) Representative images showing the Fibrillarin staining in the nucleus during cell cycle progression in U2OS FUCCI-3 cel line, as well as the corresponding signal of the FUCCI-3 fluorescent marker of the depicted cell, confirming the assigned cell cycle phase. Scale bar is 10µm. (D) Immunofluorescence-based quantification of the nuclear fibrillarin integrated intensity across cell cycle phases determined with the FUCCI-3 system. 3 biological replicates were analyzed (*n*G0 = 1306, *n*G1 = 3065, *n*S = 1164, *n*G2/M = 594, *n*M = 140; outliers removed, 3 SD; unpaired two-tailed Wilcoxon test). (E) Immunofluorescence-based quantification of the nucleolar fibrillarin mean intensity across cell cycle phases determined with the FUCCI-3 system. 3 biological replicates were analyzed (*n*G0 = 1317, *n*G1 = 3091, *n*S = 1169, *n*G2/M = 601, *n*M = 140; outliers removed, 3 SD; unpaired two-tailed Wilcoxon test). (F) Example of detection of cell cycle profiles and assignment to G1, S or G2M based on Hoechst integrated intensity. (G) Immunofluorescence-based quantification of the nucleoli area from U2OS-wt cell line across cell cycle phases determined by Hoeschst integrated intensity. 3 biological replicates were analyzed (*n*G1 = 1107, *n*S = 177, *n*G2/M = 339; outliers removed, 3 SD; unpaired two-tailed Wilcoxon test). (H) Immunofluorescence-based quantification of the nucleoli area from MCF7-wt cell line across cell cycle phases determined by Hoeschst integrated intensity. 3 biological replicates were analyzed (*n*G1 = 13906, *n*S = 3996, *n*G2/M = 3683; outliers removed, 3 SD; unpaired two-tailed Wilcoxon test). (I) Immunofluorescence-based quantification of nuclear PIP2 intensity from U2OS-wt cell line across cell cycle phases determined by Hoeschst integrated intensity. 3 biological replicates were analyzed (*n*G1 = 1099, *n*S = 178, *n*G2/M = 337; outliers removed, 3 SD; unpaired two-tailed Wilcoxon test). (J) Immunofluorescence-based quantification of the nucleolar PIP2 intesity from U2OS-wt cell line across cell cycle phases determined by Hoeschst integrated intensity. 3 biological replicates were analyzed (*n*G1 = 1105, *n*S = 179, *n*G2/M = 336; outliers removed, 3 SD; unpaired two-tailed Wilcoxon test). (K-L) Immunofluorescence-based quantification of the (K) area of PIP2 foci and (L) their number normalized to the Fibrillarin area size, classified as either inside nucleoli (identified by Fibrillarin staining) or outside nucleoli (Fibrillarin-negative regions). Experiments were performed in U2OS cells grouped by cell cycle phase using Hoechst integrated intensity. 3 biological replicates were analyzed (*n*G1 = 1102, *n*S = 177, *n*G2M = 341; outliers removed, 3 SD; unpaired two-tailed Wilcoxon test). (M) Representative confocal images showing the intensity and distribution of Fibrillarin (green) and PIP2 (red) in each identified cell cycle phase. The nucleus, identified with Hoechst staining, is shown in blue. Scale bar is 10µm.

**Supplementary Figure 11.** **Nucleoli alteration following PIP2 metabolism perturbation in MCF7-wt cell line.** (A) Immunofluorescence-based quantification nuclear PIP5K1A mean intensities across cell cycle phases using Hoechst integrated intensity in MCF7-wt cell line expressing shNTC or shPIP5K1A-UTR. 3 biological replicates were analyzed (*n*_shNTC_G1 = 2637, *n*_shPIP5K1A_G1 = 1008, *n*_shNTC_S = 854, *n*_shPIP5K1A_S = 244, *n*_shNTC_G2M = 661, *n*_shPIP5K1A_G2M = 254; outliers removed, 3 SD; unpaired two-tailed Wilcoxon test). (B) Immunofluorescence-based quantification cytoplasmic PIP5K1A mean intensities across cell cycle phases using Hoechst integrated intensity in MCF7-wt cell line expressing shNTC or shPIP5K1A-UTR. 3 biological replicates were analyzed (*n*_shNTC_G1 = 2652, *n*_shPIP5K1A_G1 = 1018, *n*_shNTC_S = 858, *n*_shPIP5K1A_S = 245, *n*_shNTC_G2M = 667, *n*_shPIP5K1A_G2M = 254; outliers removed, 3 SD; unpaired two-tailed Wilcoxon test). (C-D) Immunofluorescence-based quantification of nuclear (C) or cytoplasmic (D) HA intensities across cell cycle phases using Hoechst mean intensity in MCF7 expressing shPIP5K1A-UTR and HA-tagged PIP5K1A wt, NLS or NES variants. shNTC and HA-Empty were used as negative control for knock-down and overexpression respectively. 3 biological replicates were analyzed (*n*_shNTC_HA-Empty*_*G1 = 23655, *n*_shPIP5K1A_HA-Empty*_*G1 = 20705, *n*_shPIP5K1A_HA-PIP5K1A-wt*_*G1 = 12599, *n*_shPIP5K1A_HA-PIP5K1A-NLS*_*G1 = 2612, *n*_shPIP5K1A_HA-PIP5K1A-NES*_*G1 = 1715, *n*_shNTC_HA-Empty*_*S = 8650, *n*_shPIP5K1A_HA-Empty*_*S = 5902, *n*_shPIP5K1A_HA-PIP5K1A-wt*_*S = 4521, *n*_shPIP5K1A _HA-PIP5K1A-NLS*_*S = 771, *n*_shPIP5K1A_HA-PIP5K1A-NES*_*S = 503, *n*_shNTC_HA-Empty*_*G2 = 11731, *n*_shPIP5K1A_HA-Empty*_*G2 = 8047, *n*_shPIP5K1A_HA-PIP5K1A-wt*_*G2 = 6221, *n*_shPIP5K1A_HA-PIP5K1A-NLS*_*G2 = 1294, *n*_shPIP5K1A_HA-PIP5K1A-NES*_*G2 = 696; outliers removed, 3 SD; unpaired two-tailed Wilcoxon test).

**Supplementary Figure 12.** **PIP5K1A inhibition arrests cell cycle and alters PIP2 in nucleoli.** (A) Dose-response curve of U2OS FUCCI-3 cells treated with ISA-2011B (PIP5K1A inhibitor) for 48h to determine the IC50. (B) Growth curve of U2OS FUCCI-3 cells treated with DMSO (negative control) or ISA-2011B (PIP5K1A inhibitor; 41.33 μM). FUCCI-3 U2OS cells were treated for 60 hours and nuclei were identified by the expression of FUCCI-3 fluorescent markers and counted over time to determine cell growth. Tracking was performed with a minimum of 150 cells per treatment. (C) FUCCI-3 plot representing the cell cycle alterations of U2OS FUCCI-3 cells treated with ISA-2011B (PIP5K1A inhibitor; 41.33 μM) for 60 hours. (D) Live cell imaging of cells treated with ISA-2011B (PIP5K1A inhibitor; 41.33 μM) monitored for 60 hours. Changes in the number of Clover-Geminin negative (G1) and positive (S & G2) cells are shown in the ridge plot, illustrating the cell cycle distribution from the beginning of the treatment and every 6 hours. (E) Quantification of time spent by U2OS FUCCI-3 cells in the phase that they were at the moment of starting the treatment with DMSO or ISA-2011B (PIP5K1A inhibitor; 41.33 μM). 3 biological replicates were analyzed (*n*DMSO_G1 = 279, *n*ISA-2011B_G1 = 500, *n*DMSO_S = 507, *n*ISA-2011B = 847, *n*DMSO_G2M = 266, *n*ISA-2011B_G2M = 361, *n*DMSO_M = 23, *n*ISA-2011B_M = 35; outliers removed, 3 SD; unpaired two-tailed Wilcoxon test). (F) FUCCI-3 plots representing the cell cycle integrity of the U2OS FUCCI-3 cell population fixed and stained for PIP5K1A quantification after treatment with DMSO or ISA-2011B (PIP5K1A inhibitor; 41.33 μM) for 48h. (G) Representation of PIP5K1A nuclear intensity measured by immunofluorescence and plotted according to FUCCI-3-determined cell cycle phases, after treatment with DMSO or ISA-2011B (PIP5K1A inhibitor; 41.33 μM) for 48h. (H) Immunofluorescence-based quantification of the nuclear intensity of PIP5K1A across cell cycle phases determined with the FUCCI-3 system in cells treated with DMSO or ISA-2011B (41.33 μM).3 biological replicates were analyzed (*n*DMSO_G0 = 906, *n*ISA-2011B_G0 = 1460, *n*DMSO_G1 = 2081, *n*ISA-2011B_G1 = 1662, *n*DMSO_S = 1355, *n*ISA-2011B = 722, *n*DMSO_G2M = 878, *n*ISA-2011B_G2M = 622, *n*DMSO_M = 107, *n*ISA-2011B_M = 33; outliers removed, 3 SD; unpaired two-tailed Wilcoxon test). (I) Representative images showing the changes of PIP5K1A in the nucleus during cell cycle progression, as well as the corresponding signal of the FUCCI-3 fluorescent marker of the depicted cell, confirming the assigned cell cycle phase in cells treated with DMSO or ISA-2011B (41.33 μM). (J) Immunofluorescence-based quantification of the mean intensity of PIP2 in nuclei, performed in cell cycle stratified population based on Hoechst integrated intensity after DMSO or ISA-2011B (41.33 μM) treatments. 3 biological replicates were analyzed (*n*DMSO_G1 = 568, *n*ISA-2011B_G1 = 471, *n*DMSO_S = 77, *n*ISA-2011B_S = 49, *n*DMSO_G2M = 188 and *n*ISA-2011B_G2M = 144; (outliers removed, 3 SD; unpaired two-tailed Wilcoxon test). (K) Representative confocal images showing the intensity and distribution of Fibrillarin (green) and PIP2 (red) in the G1 phase in cells treated with DMSO or ISA-2011B (41.33 μM). The nucleus, identified with Hoechst staining, is shown in blue. The segmentation of the Fibrillarin region is shown as a green outline in the merged image, while PIP2 is shown in FIRE_lut. Scale bar is 10µm. (L) Immunofluorescence-based quantification of the mean intensity of PIP2 in nucleoli, performed in cell cycle stratified population based on Hoechst integrated intensity after DMSO or ISA-2011B (41.33 μM) treatments. 3 biological replicates were analyzed (*n*DMSO_G1 = 1115, *n*ISA-2011B_G1 = 932, *n*DMSO_S = 154, *n*ISA-2011B_S = 100, *n*DMSO_G2M = 372 and *n*ISA-2011B_G2M = 287; (outliers removed, 3 SD; unpaired two-tailed Wilcoxon test).

**Supplementary Figure 13. H4K20me1 oscillating during cell cycle in U2OS and MCF7 FUCCI-3 cell lines.** (A) FUCCI-3 plot representing the cell cycle integrity of the U2OS FUCCI-3 cell population fixed and stained for H4K20me1 quantification. (B) Representation of H4K20me1 nuclear intensity measured by immunofluorescence and plotted according to FUCCI-3-determined cell cycle phases. (C) Immunofluorescence-based quantification of the mean intensity of H4K20me1 across cell cycle phases determined with the FUCCI-3 system. 3 biological replicates were analyzed (*n*G0 = 1325, *n*G1 = 2795, *n*S = 1192, *n*G2/M = 623, *n*M = 118; outliers removed, 3 SD; unpaired two-tailed Wilcoxon test). (D) Immunofluorescence-based quantification of the integrated intensity of H4K20me1 across cell cycle phases determined with the FUCCI-3 system. 3 biological replicates were analyzed (*n*G0 = 1322, *n*G1 = 2772, *n*S = 1188, *n*G2/M = 623, *n*M = 119; outliers removed, 3 SD; unpaired two-tailed Wilcoxon test). (E) FUCCI-3 plot representing the cell cycle integrity of the MCF7 FUCCI-3 cell population fixed and stained for H4K20me1 quantification. (F) Representation of H4K20me1 nuclear intensity measured by immunofluorescence and plotted according to FUCCI-3-determined cell cycle phases. (G) Immunofluorescence-based quantification of the mean intensity of H4K20me1 across cell cycle phases determined with the FUCCI-3 system. 3 biological replicates were analyzed (*n*G0 = 2747, *n*G1 = 8164, *n*S = 3944, *n*G2/M = 2200, *n*M = 761; outliers removed, 3 SD; unpaired two-tailed Wilcoxon test). (H) Immunofluorescence-based quantification of the integrated intensity of H4K20me1 across cell cycle phases determined with the FUCCI-3 system. 3 biological replicates were analyzed (*n*G0 = 2730, *n*G1 = 8099, *n*S = 3929, *n*G2/M = 2198, *n*M = 761; outliers removed, 3 SD; unpaired two-tailed Wilcoxon test).

**Supplementary Figure 14. Methylation defects linked to PIP2 metabolism perturbation.** (A) FUCCI-3 plot representing the cell cycle integrity of the U2OS cells treated with DMSO or ISA-2011B (41.33 μM) for 48 hours, fixed, and stained for H4K20me1. (B) Representation of H4K20me1 nuclear intensity measured by immunofluorescence and plotted according to FUCCI-3-determined cell cycle phases in U2OS cells treated either with DMSO or ISA-2011B (41.33 μM) for 48 hours. (C) Immunofluorescence-based quantification of H4K20me1 intensities across cell cycle phases determined with the FUCCI-3 system in U2OS cells treated with DMSO or ISA-2011B (41.33 μM). 3 biological replicates were analyzed (*n*DMSO_G0 = 878, *n*ISA-2011B_G0 = 962, *n*DMSO_G1 = 1644, *n*ISA-2011B_G1 = 1206, *n*DMSO_S = 933, *n*ISA-2011B = 423, *n*DMSO_G2M = 823, *n*ISA-2011B_G2M = 469, *n*DMSO_M = 72, ISA-2011B_M = 27; outliers removed, 3 SD; unpaired two-tailed Wilcoxon test). The lower panels display representative images showing the changes of H4K20me1 in the nucleus during cell cycle progression, as well as the corresponding signal of the FUCCI-3 fluorescent marker of the depicted cell, confirming the assigned cell cycle phase. Scale bar is 20µm. (D) FUCCI-3 plot representing the cell cycle integrity of the U2OS cells treated with DMSO or ISA-2011B (41.33 μM) for 48 hours, fixed, and stained for H3K9me3. (E) Representation of H3K9me3 nuclear intensity measured by immunofluorescence and plotted according to FUCCI-3-determined cell cycle phases in U2OS cells treated either with DMSO or ISA-2011B (41.33 μM) for 48 hours. (F) Immunofluorescence-based quantification of H3K9me3 intensities across cell cycle phases determined with the FUCCI-3 system in U2OS cells treated with DMSO or ISA-2011B (41.33 μM). 3 biological replicates were analyzed (*n*DMSO_G0 = 933, *n*ISA-2011B_G0 = 1475, *n*DMSO_G1 = 1686, *n*ISA-2011B_G1 = 1573, *n*DMSO_S = 1000, *n*ISA-2011B = 671, *n*DMSO_G2M = 857, *n*ISA-2011B_G2M = 720, *n*DMSO_M = 91, ISA-2011B_M = 39; outliers removed, 3 SD; unpaired two-tailed Wilcoxon test). The lower panels display representative images showing the changes of H3K9me3 in the nucleus during cell cycle progression, as well as the corresponding signal of the FUCCI-3 fluorescent marker of the depicted cell, confirming the assigned cell cycle phase. Scale bar is 20µm. (G) FUCCI-3 plot representing the cell cycle integrity of the U2OS cells treated with DMSO or ISA-2011B (41.33 μM) for 48 hours, fixed, and stained for H3K27me3. (H) Representation of H3K27me3 nuclear intensity measured by immunofluorescence and plotted according to FUCCI-3-determined cell cycle phases in U2OS cells treated either with DMSO or ISA-2011B (41.33 μM) for 48 hours. (I) Immunofluorescence-based quantification of H3K27me3 intensities across cell cycle phases determined with the FUCCI-3 system in U2OS cells treated with DMSO or ISA-2011B (41.33 μM). 3 biological replicates were analyzed (*n*DMSO_G0 = 1033, *n*ISA-2011B_G0 = 1309, *n*DMSO_G1 = 1878, *n*ISA-2011B_G1 = 1436, *n*DMSO_S = 1051, *n*ISA-2011B = 631, *n*DMSO_G2M = 944, *n*ISA-2011B_G2M = 568, *n*DMSO_M = 116, ISA-2011B_M = 28; outliers removed, 3 SD; unpaired two-tailed Wilcoxon test). The lower panels display representative images showing the changes of H3K27me3 in the nucleus during cell cycle progression, as well as the corresponding signal of the FUCCI-3 fluorescent marker of the depicted cell, confirming the assigned cell cycle phase. Scale bar is 20µm.

**Supplementary Figure 15. Methylation defects linked to PIP2 metabolism perturbation.** (A) Immunofluorescence-based quantification of H4K20me1 mean intensities across cell cycle phases using Hoechst integrated intensity in MCF7-wt cell line expressing shNTC or shPIP5K1A-UTR. 3 biological replicates were analyzed (*n*_shNTC_G1 = 7010, *n*_shPIP5K1A_G1 = 5267, *n*_shNTC_S = 2431, *n*_shPIP5K1A_S = 1542, *n*_shNTC_G2M = 3369, *n*_shPIP5K1A_G2M = 1909; outliers removed, 3 SD; unpaired two-tailed Wilcoxon test). (B) Immunofluorescence-based quantification of H3K9me3 mean intensities across cell cycle phases using Hoechst integrated intensity in MCF7-wt cell line expressing shNTC or shPIP5K1A-UTR. 3 biological replicates were analyzed (*n*_shNTC_G1 = 6227, *n*_shPIP5K1A_G1 = 5262, *n*_shNTC_S = 2266, *n*_shPIP5K1A_S = 1427, *n*_shNTC_G2M = 2886, *n*_shPIP5K1A_G2M = 1981; outliers removed, 3 SD; unpaired two-tailed Wilcoxon test). (C) Immunofluorescence-based quantification of H3K27me3 mean intensities across cell cycle phases using Hoechst integrated intensity in MCF7-wt cell line expressing shNTC or shPIP5K1A-UTR. 3 biological replicates were analyzed (*n*_shNTC_G1 = 4873, *n*_shPIP5K1A_G1 = 4956, *n*_shNTC_S = 1898, *n*_shPIP5K1A_S = 1347, *n*_shNTC_G2M = 2569, *n*_shPIP5K1A_G2M = 1933; outliers removed, 3 SD; unpaired two-tailed Wilcoxon test). (D) Immunofluorescence-based quantification of H3K4me3 mean intensities across cell cycle phases using Hoechst integrated intensity in MCF7-wt cell line expressing shNTC or shPIP5K1A-UTR. 3 biological replicates were analyzed (*n*_shNTC_G1 = 5544, *n*_shPIP5K1A_G1 = 5174, *n*_shNTC_S = 2053, *n*_shPIP5K1A_S = 1544, *n*_shNTC_G2M = 2764, *n*_shPIP5K1A_G2M = 2065; outliers removed, 3 SD; unpaired two-tailed Wilcoxon test).
