# Supplementary material for: Cell Cycle Control of Nuclear Metabolism Couples Phosphatidylinositol Signaling to Histone Methylation
Source: Adv Sci (Weinh). 2026 Apr 21;13(34):e01083. doi: 10.1002/advs.202501083 (PMC13285136; doi:10.1002/advs.202501083)
Supplement: Supplementary file 2 — Supporting File 2: advs75068‐sup‐0002‐FigureS1‐S15.pdf. [file ADVS-13-e01083-s001.pdf]

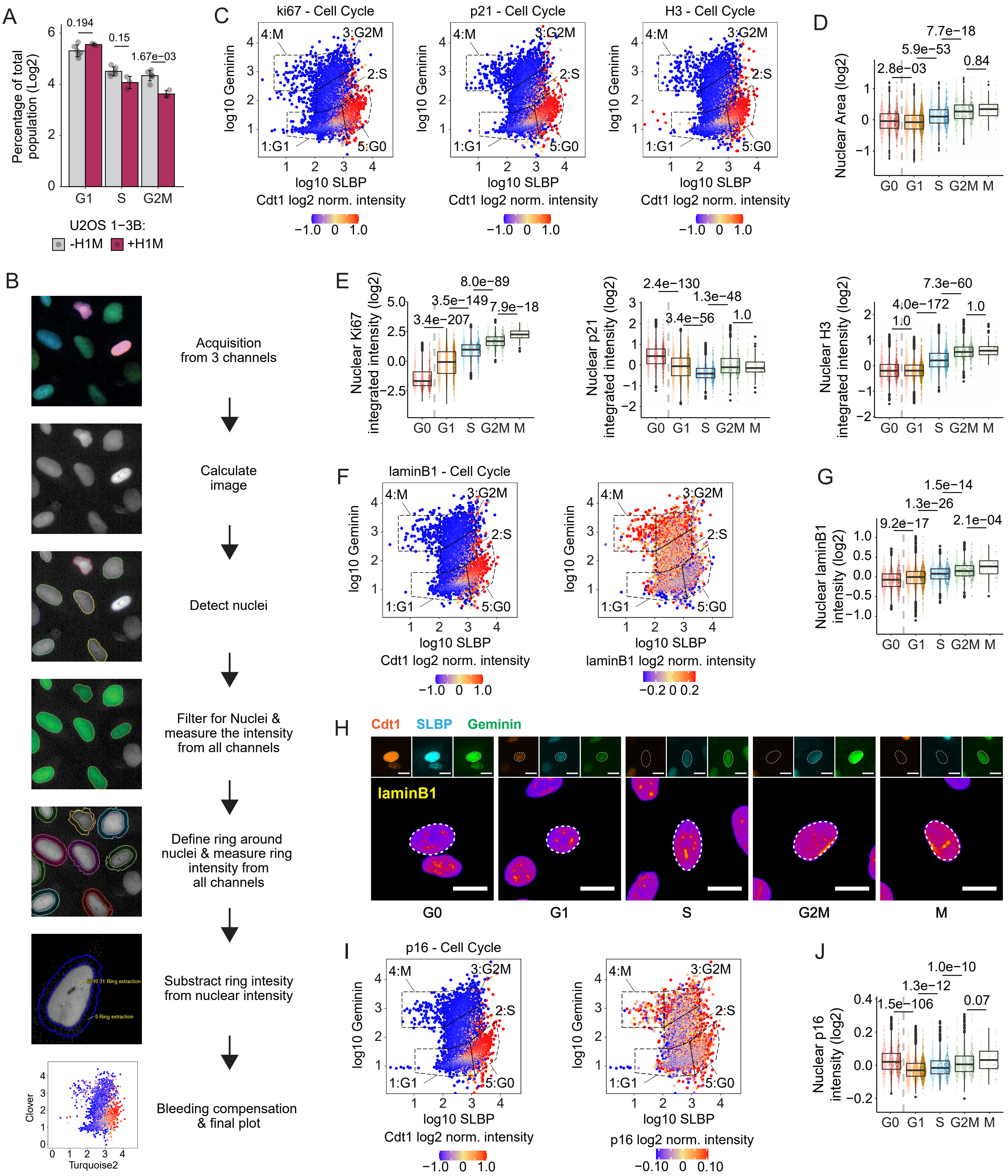

A
Percentage of total population (Log2)
0.194
0.15
1.67e-03
G1
S
G2M
U2OS 1-3B:
-H1M
+H1M
C
ki67 - Cell Cycle
p21 - Cell Cycle
H3 - Cell Cycle
log10 Geminin
log10 SLBP
1:G1
2:S
3:G2M
4:M
5:G0
Cdt1 log2 norm. intensity
D
Nuclear Area (log2)
2.8e-03
5.9e-53
7.7e-18
0.84
G0 G1 S G2M M
B
Acquisition from 3 channels
Calculate image
Detect nuclei
Filter for Nuclei & measure the intensity from all channels
Define ring around nuclei & measure ring intensity from all channels
Substract ring intesity from nuclear intensity
Bleeding compensation & final plot
Clover
Turquoise2
E
Nuclear Ki67 integrated intensity (log2)
3.4e-207
3.5e-149
8.0e-89
7.9e-18
Nuclear p21 integrated intensity (log2)
2.4e-130
3.4e-56
1.3e-48
1.0
Nuclear H3 integrated intensity (log2)
1.0
4.0e-172
7.3e-60
1.0
F
laminB1 - Cell Cycle
laminB1 log2 norm. intensity
G
Nuclear laminB1 intensity (log2)
9.2e-17
1.3e-26
1.5e-14
2.1e-04
H
Cdt1
SLBP
Geminin
laminB1
G0
G1
S
G2M
M
I
p16 - Cell Cycle
p16 log2 norm. intensity
J
Nuclear p16 intensity (log2)
1.5e-106
1.3e-12
1.0e-10
0.07

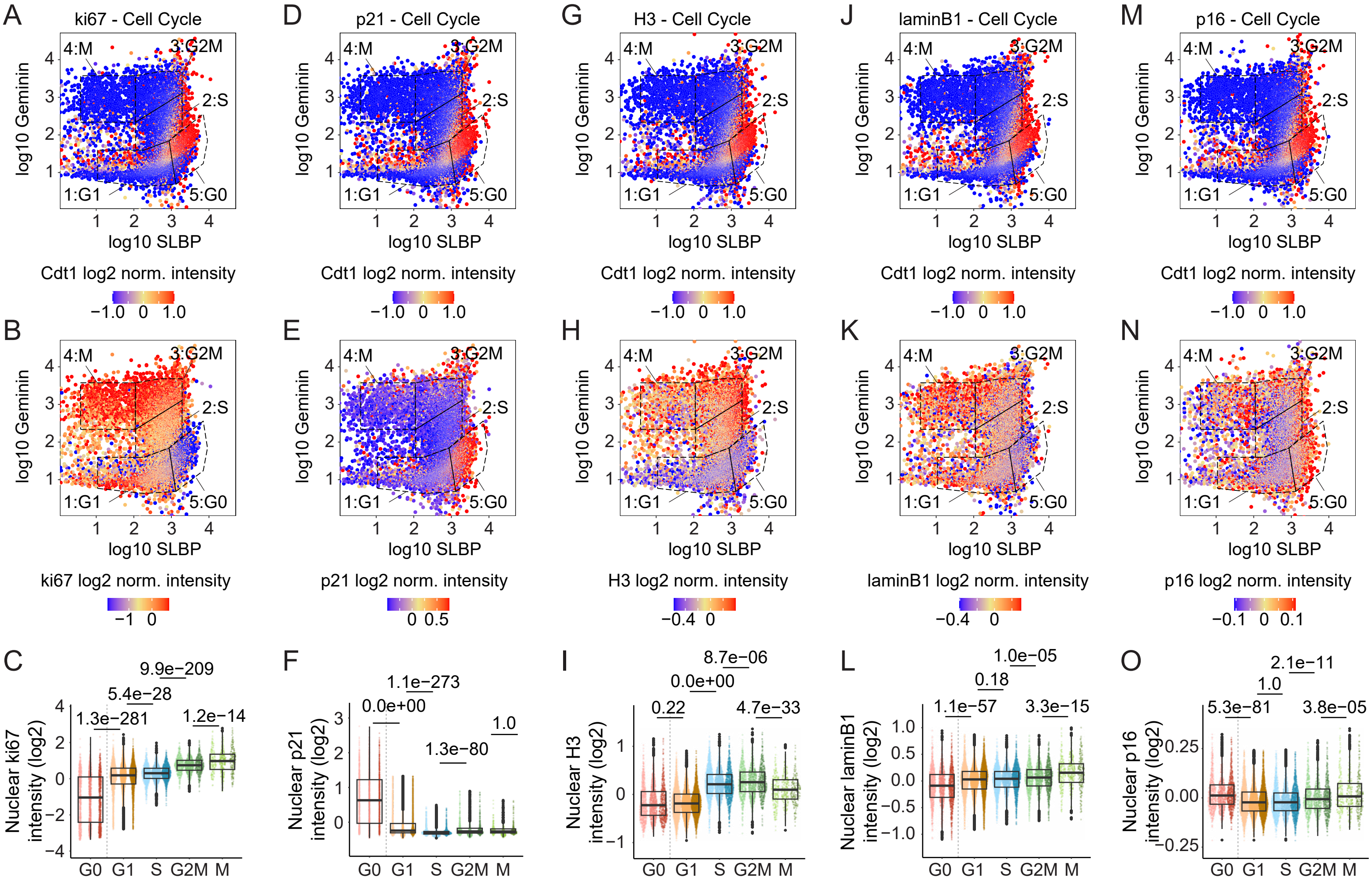
A
ki67 - Cell Cycle
D
p21 - Cell Cycle
G
H3 - Cell Cycle
J
laminB1 - Cell Cycle
M
p16 - Cell Cycle
4:M
3:G2M
2:S
1:G1
5:G0
log10 Geminin
log10 SLBP
Cdt1 log2 norm. intensity
−1.0 0 1.0
B
E
H
K
N
ki67 log2 norm. intensity
−1 0
p21 log2 norm. intensity
0 0.5
H3 log2 norm. intensity
−0.4 0
laminB1 log2 norm. intensity
−0.4 0
p16 log2 norm. intensity
−0.1 0 0.1
C
9.9e−209
5.4e−28
1.3e−281
1.2e−14
Nuclear ki67 intensity (log2)
F
1.1e−273
0.0e+00
1.0
1.3e−80
Nuclear p21 intensity (log2)
I
8.7e−06
0.0e+00
0.22
4.7e−33
Nuclear H3 intensity (log2)
L
1.0e−05
0.18
1.1e−57
3.3e−15
Nuclear laminB1 intensity (log2)
O
2.1e−11
1.0
5.3e−81
3.8e−05
Nuclear p16 intensity (log2)
G0 G1 S G2M M

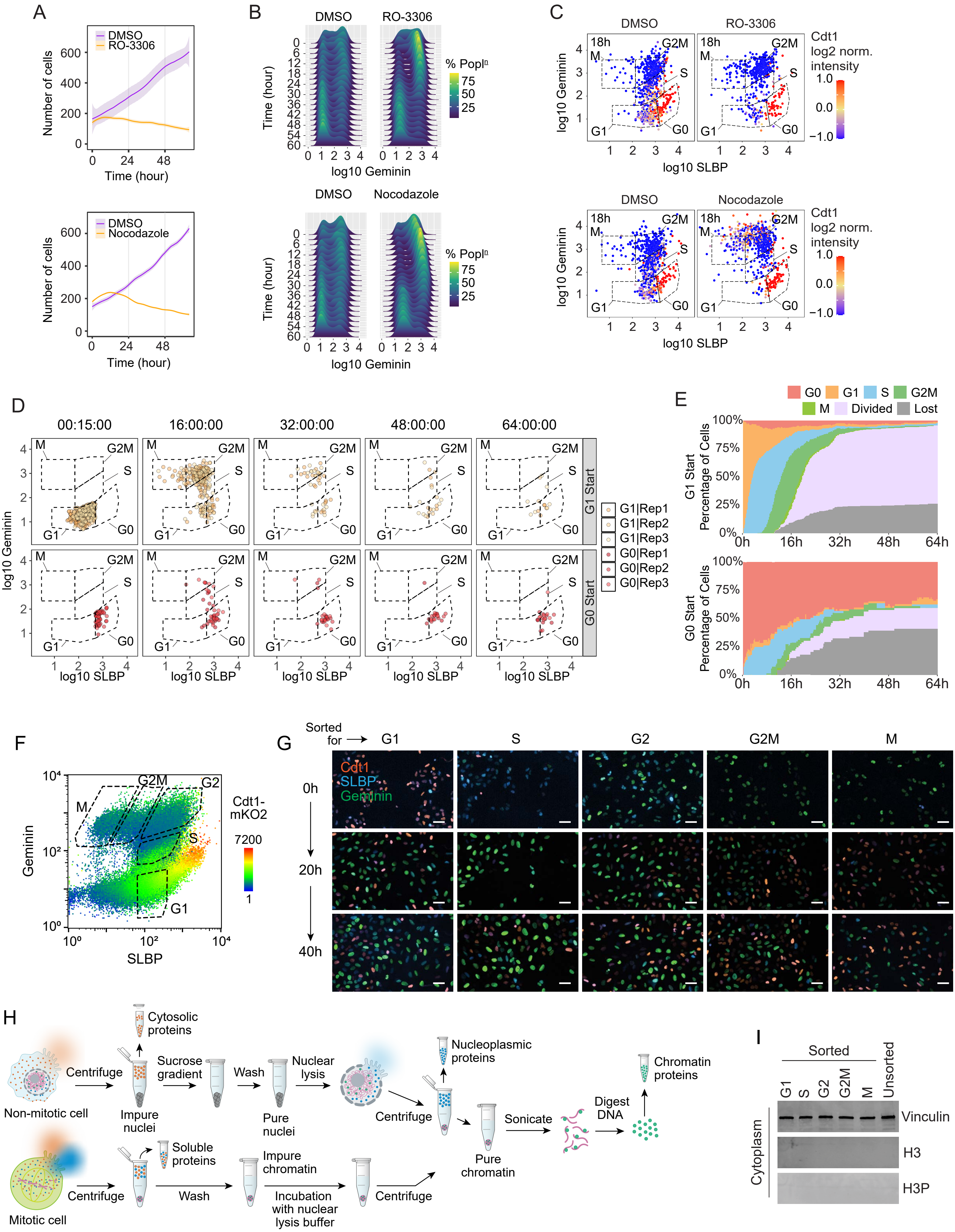

A
DMSO
RO-3306
Nocodazole
Number of cells
Time (hour)
B
DMSO
RO-3306
Nocodazole
Time (hour)
log10 Geminin
% Popln
C
18h
G2M
M
S
G1
G0
log10 Geminin
log10 SLBP
Cdt1 log2 norm. intensity
D
00:15:00
16:00:00
32:00:00
48:00:00
64:00:00
G1 Start
G0 Start
G1|Rep1
G1|Rep2
G1|Rep3
G0|Rep1
G0|Rep2
G0|Rep3
E
G0
G1
S
G2M
M
Divided
Lost
G1 Start Percentage of Cells
G0 Start Percentage of Cells
0h
16h
32h
48h
64h
F
Geminin
SLBP
Cdt1-mKO2
G2
G
Sorted for
G1
S
G2
G2M
M
Cdt1
SLBP
Geminin
0h
20h
40h
H
Non-mitotic cell
Centrifuge
Cytosolic proteins
Impure nuclei
Sucrose gradient
Wash
Pure nuclei
Nuclear lysis
Nucleoplasmic proteins
Pure chromatin
Sonicate
Digest DNA
Chromatin proteins
Mitotic cell
Soluble proteins
Impure chromatin
Incubation with nuclear lysis buffer
I
Sorted
Unsorted
Cytoplasm
Vinculin
H3
H3P

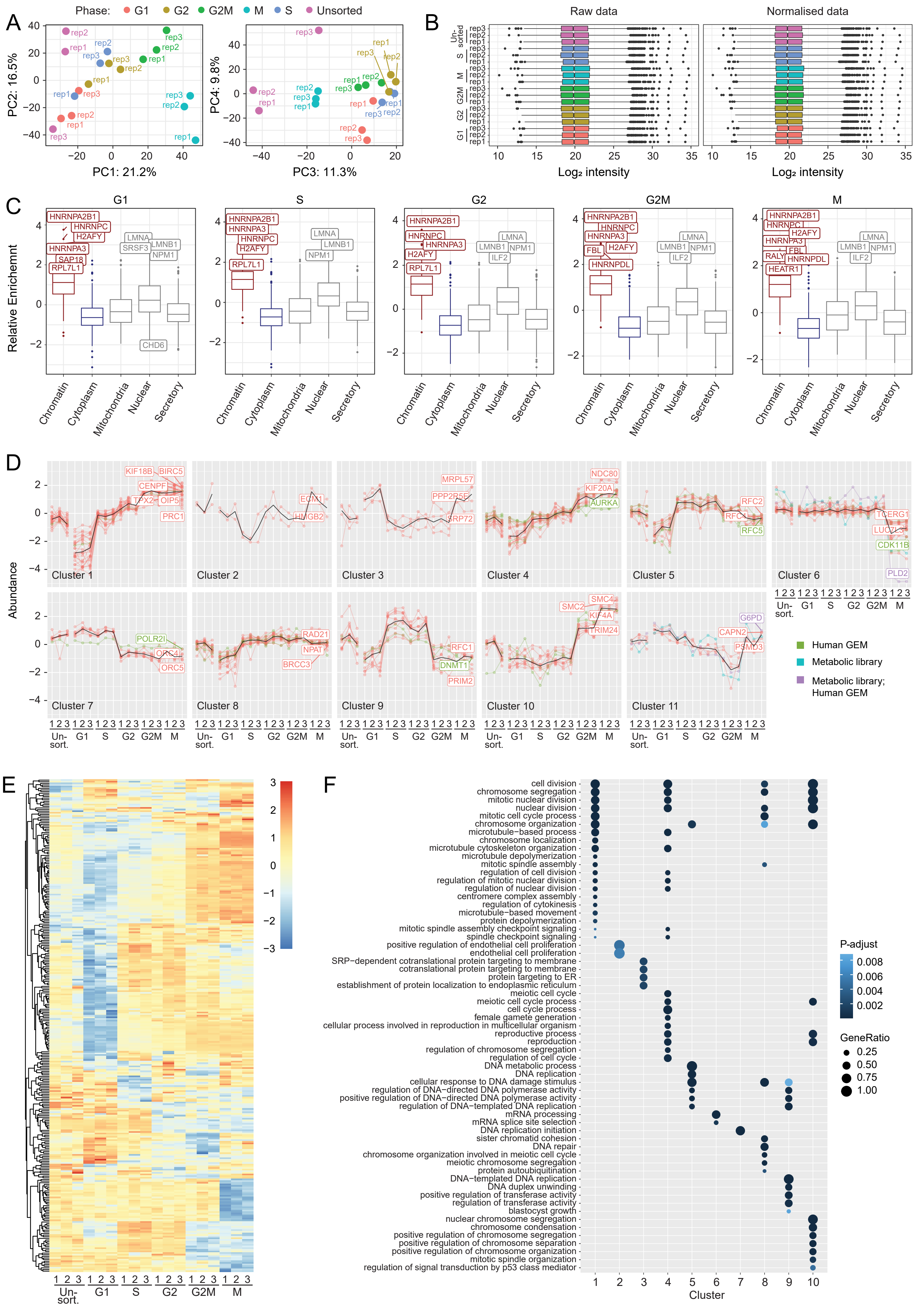
A
Phase: G1 G2 G2M M S Unsorted
PC1: 21.2%
PC2: 16.5%
PC3: 11.3%
PC4: 9.8%
B
Raw data
Normalised data
Log2 intensity
Un-sorted
C
G1
S
G2
G2M
M
Relative Enrichemnt
Chromatin
Cytoplasm
Mitochondria
Nuclear
Secretory
D
Abundance
Cluster 1
Cluster 2
Cluster 3
Cluster 4
Cluster 5
Cluster 6
Cluster 7
Cluster 8
Cluster 9
Cluster 10
Cluster 11
Human GEM
Metabolic library
Metabolic library; Human GEM
E
F
cell division
chromosome segregation
mitotic nuclear division
nuclear division
mitotic cell cycle process
chromosome organization
microtubule−based process
chromosome localization
microtubule cytoskeleton organization
microtubule depolymerization
mitotic spindle assembly
regulation of cell division
regulation of mitotic nuclear division
regulation of nuclear division
centromere complex assembly
regulation of cytokinesis
microtubule−based movement
protein depolymerization
mitotic spindle assembly checkpoint signaling
spindle checkpoint signaling
positive regulation of endothelial cell proliferation
endothelial cell proliferation
SRP−dependent cotranslational protein targeting to membrane
cotranslational protein targeting to membrane
protein targeting to ER
establishment of protein localization to endoplasmic reticulum
meiotic cell cycle
meiotic cell cycle process
cell cycle process
female gamete generation
cellular process involved in reproduction in multicellular organism
reproductive process
reproduction
regulation of chromosome segregation
regulation of cell cycle
DNA metabolic process
DNA replication
cellular response to DNA damage stimulus
regulation of DNA−directed DNA polymerase activity
positive regulation of DNA−directed DNA polymerase activity
regulation of DNA−templated DNA replication
mRNA processing
mRNA splice site selection
DNA replication initiation
sister chromatid cohesion
DNA repair
chromosome organization involved in meiotic cell cycle
meiotic chromosome segregation
protein autoubiquitination
DNA−templated DNA replication
DNA duplex unwinding
positive regulation of transferase activity
regulation of transferase activity
blastocyst growth
nuclear chromosome segregation
chromosome condensation
positive regulation of chromosome segregation
positive regulation of chromosome separation
positive regulation of chromosome organization
mitotic spindle organization
regulation of signal transduction by p53 class mediator
Cluster
P-adjust
GeneRatio

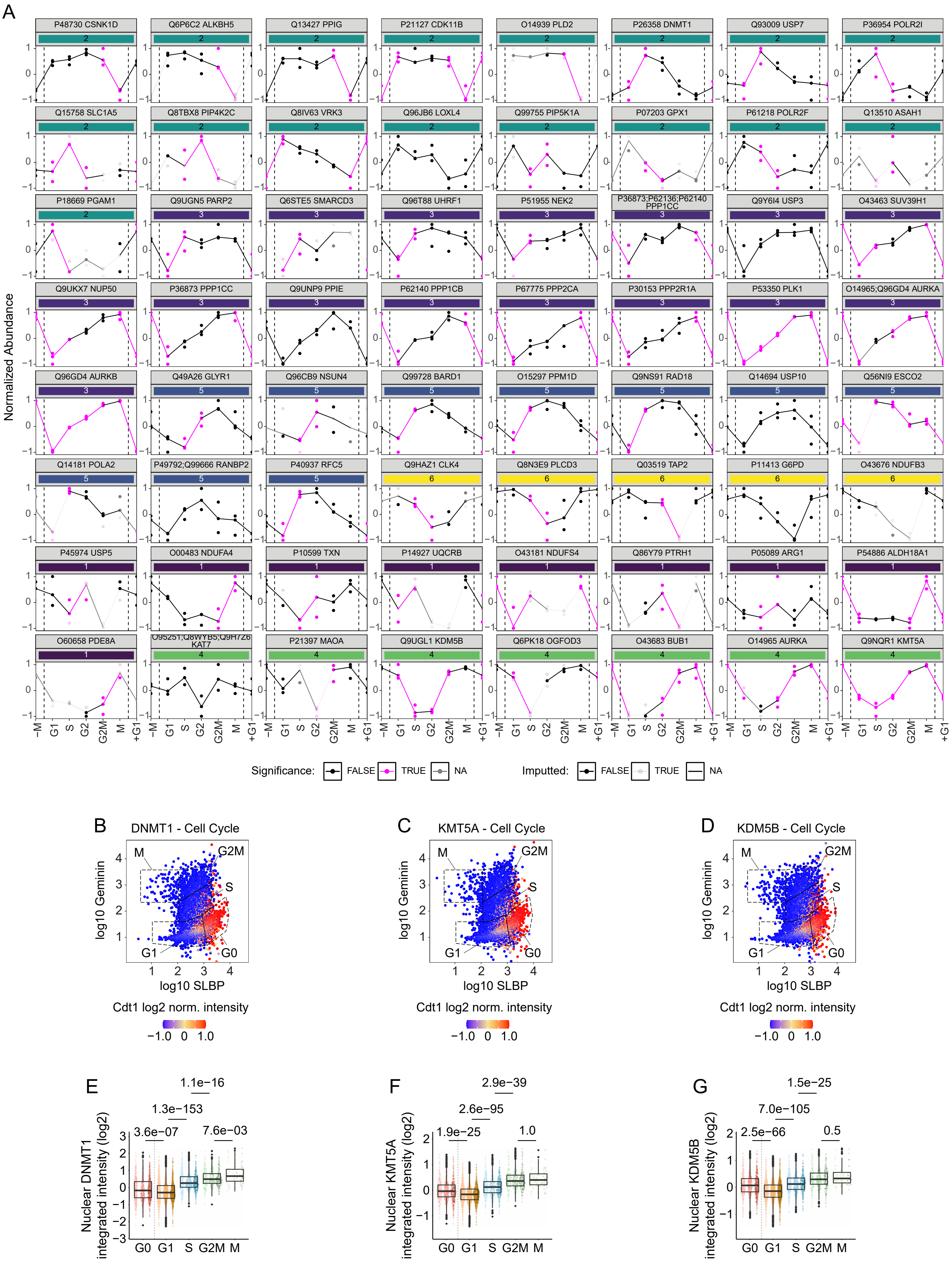
A
P48730 CSNK1D
Q6P6C2 ALKBH5
Q13427 PPIG
P21127 CDK11B
O14939 PLD2
P26358 DNMT1
Q93009 USP7
P36954 POLR2I
Q15758 SLC1A5
Q8TBX8 PIP4K2C
Q8IV63 VRK3
Q96JB6 LOXL4
Q99755 PIP5K1A
P07203 GPX1
P61218 POLR2F
Q13510 ASAH1
P18669 PGAM1
Q9UGN5 PARP2
Q6STE5 SMARCD3
Q96T88 UHRF1
P51955 NEK2
P36873;P62136;P62140 PPP1CC
Q9Y6I4 USP3
O43463 SUV39H1
Q9UKX7 NUP50
P36873 PPP1CC
Q9UNP9 PPIE
P62140 PPP1CB
P67775 PPP2CA
P30153 PPP2R1A
P53350 PLK1
O14965;Q96GD4 AURKA
Q96GD4 AURKB
Q49A26 GLYR1
Q96CB9 NSUN4
Q99728 BARD1
O15297 PPM1D
Q9NS91 RAD18
Q14694 USP10
Q56NI9 ESCO2
Q14181 POLA2
P49792;Q99666 RANBP2
P40937 RFC5
Q9HAZ1 CLK4
Q8N3E9 PLCD3
Q03519 TAP2
P11413 G6PD
O43676 NDUFB3
P45974 USP5
O00483 NDUFA4
P10599 TXN
P14927 UQCRB
O43181 NDUFS4
Q86Y79 PTRH1
P05089 ARG1
P54886 ALDH18A1
O60658 PDE8A
O95251;Q8WYB5;Q9H7Z6 KAT7
P21397 MAOA
Q9UGL1 KDM5B
Q6PK18 OGFOD3
O43683 BUB1
O14965 AURKA
Q9NQR1 KMT5A
Normalized Abundance
−M G1 S G2 G2M M +G1
Significance: FALSE TRUE NA
Imputted: FALSE TRUE NA
B
DNMT1 - Cell Cycle
C
KMT5A - Cell Cycle
D
KDM5B - Cell Cycle
M
G2M
S
G1
G0
log10 Geminin
log10 SLBP
Cdt1 log2 norm. intensity
−1.0 0 1.0
E
1.1e−16
1.3e−153
3.6e−07
7.6e−03
Nuclear DNMT1 integrated intensity (log2)
F
2.9e−39
2.6e−95
1.9e−25
1.0
Nuclear KMT5A integrated intensity (log2)
G
1.5e−25
7.0e−105
2.5e−66
0.5
Nuclear KDM5B integrated intensity (log2)
G0 G1 S G2M M

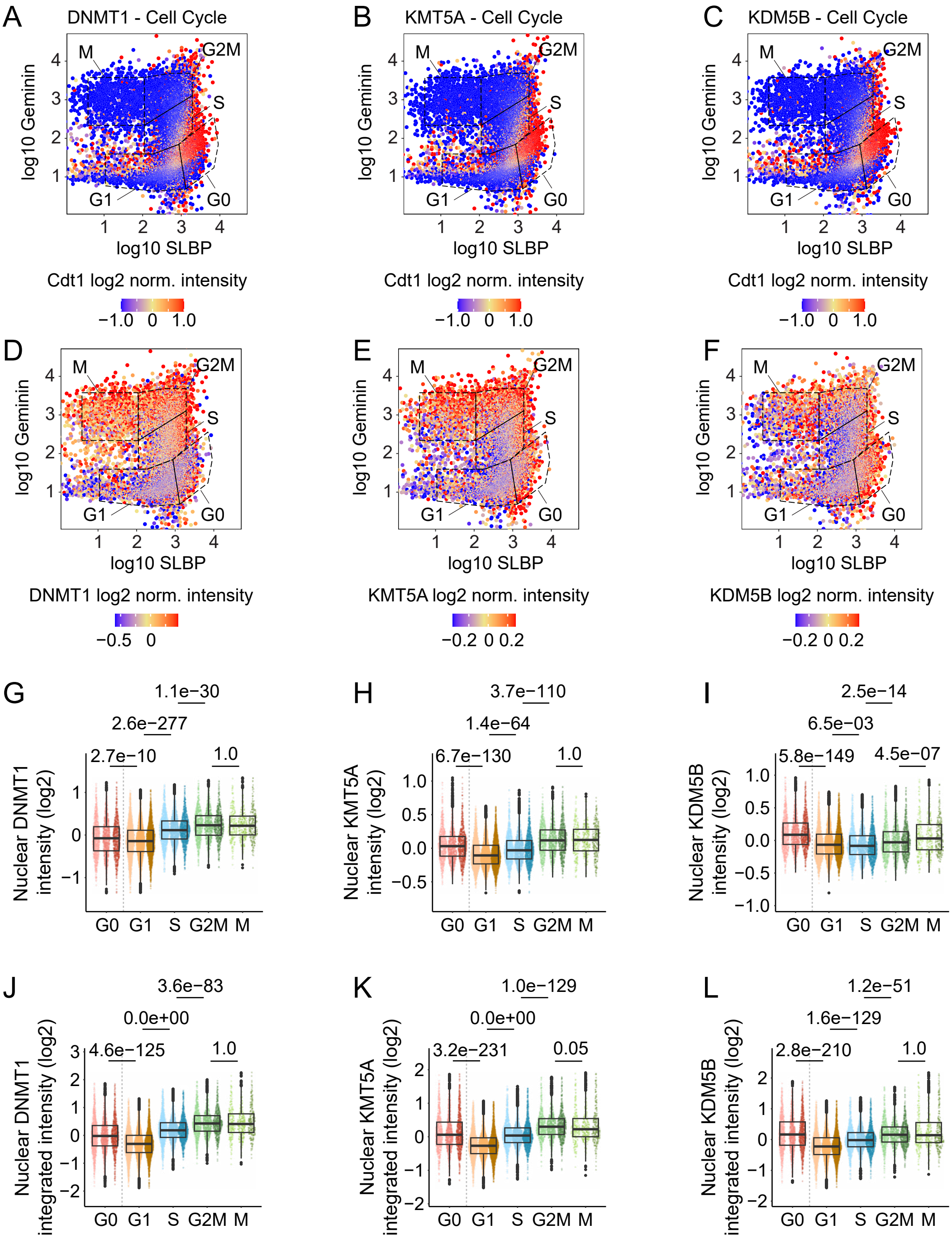
A DNMT1 - Cell Cycle
B KMT5A - Cell Cycle
C KDM5B - Cell Cycle
log10 Geminin
log10 SLBP
M
G2M
S
G1
G0
Cdt1 log2 norm. intensity
−1.0 0 1.0
D
E
F
DNMT1 log2 norm. intensity
−0.5 0
KMT5A log2 norm. intensity
−0.2 0 0.2
KDM5B log2 norm. intensity
−0.2 0 0.2
G
1.1e−30
2.6e−277
2.7e−10
1.0
Nuclear DNMT1 intensity (log2)
H
3.7e−110
1.4e−64
6.7e−130
1.0
Nuclear KMT5A intensity (log2)
I
2.5e−14
6.5e−03
5.8e−149
4.5e−07
Nuclear KDM5B intensity (log2)
G0 G1 S G2M M
J
3.6e−83
0.0e+00
4.6e−125
1.0
Nuclear DNMT1 integrated intensity (log2)
K
1.0e−129
0.0e+00
3.2e−231
0.05
Nuclear KMT5A integrated intensity (log2)
L
1.2e−51
1.6e−129
2.8e−210
1.0
Nuclear KDM5B integrated intensity (log2)

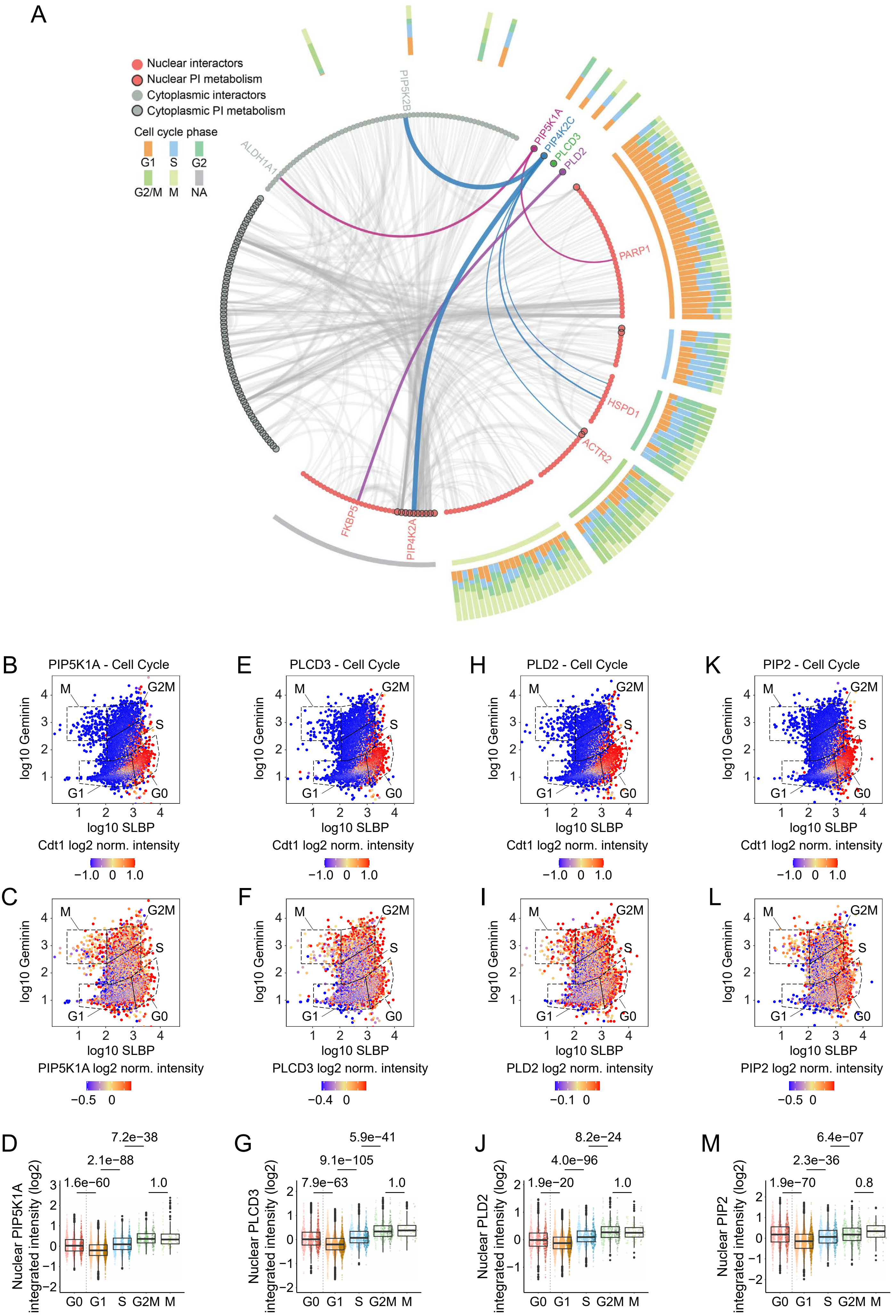

A
Nuclear interactors
Nuclear PI metabolism
Cytoplasmic interactors
Cytoplasmic PI metabolism
Cell cycle phase
G1
S
G2
G2/M
M
NA
PIP5K2B
ALDH1A1
PIP5K1A
PIP4K2C
PLCD3
PLD2
PARP1
HSPD1
ACTR2
FKBP5
PIP4K2A
B
PIP5K1A - Cell Cycle
E
PLCD3 - Cell Cycle
H
PLD2 - Cell Cycle
K
PIP2 - Cell Cycle
log10 Geminin
log10 SLBP
M
G2M
S
G1
G0
Cdt1 log2 norm. intensity
−1.0 0 1.0
C
F
I
L
PIP5K1A log2 norm. intensity
−0.5 0
PLCD3 log2 norm. intensity
−0.4 0
PLD2 log2 norm. intensity
−0.1 0
PIP2 log2 norm. intensity
−0.5 0
D
Nuclear PIP5K1A integrated intensity (log2)
7.2e−38
2.1e−88
1.6e−60
1.0
G
Nuclear PLCD3 integrated intensity (log2)
5.9e−41
9.1e−105
7.9e−63
1.0
J
Nuclear PLD2 integrated intensity (log2)
8.2e−24
4.0e−96
1.9e−20
1.0
M
Nuclear PIP2 integrated intensity (log2)
6.4e−07
2.3e−36
1.9e−70
0.8
G0 G1 S G2M M

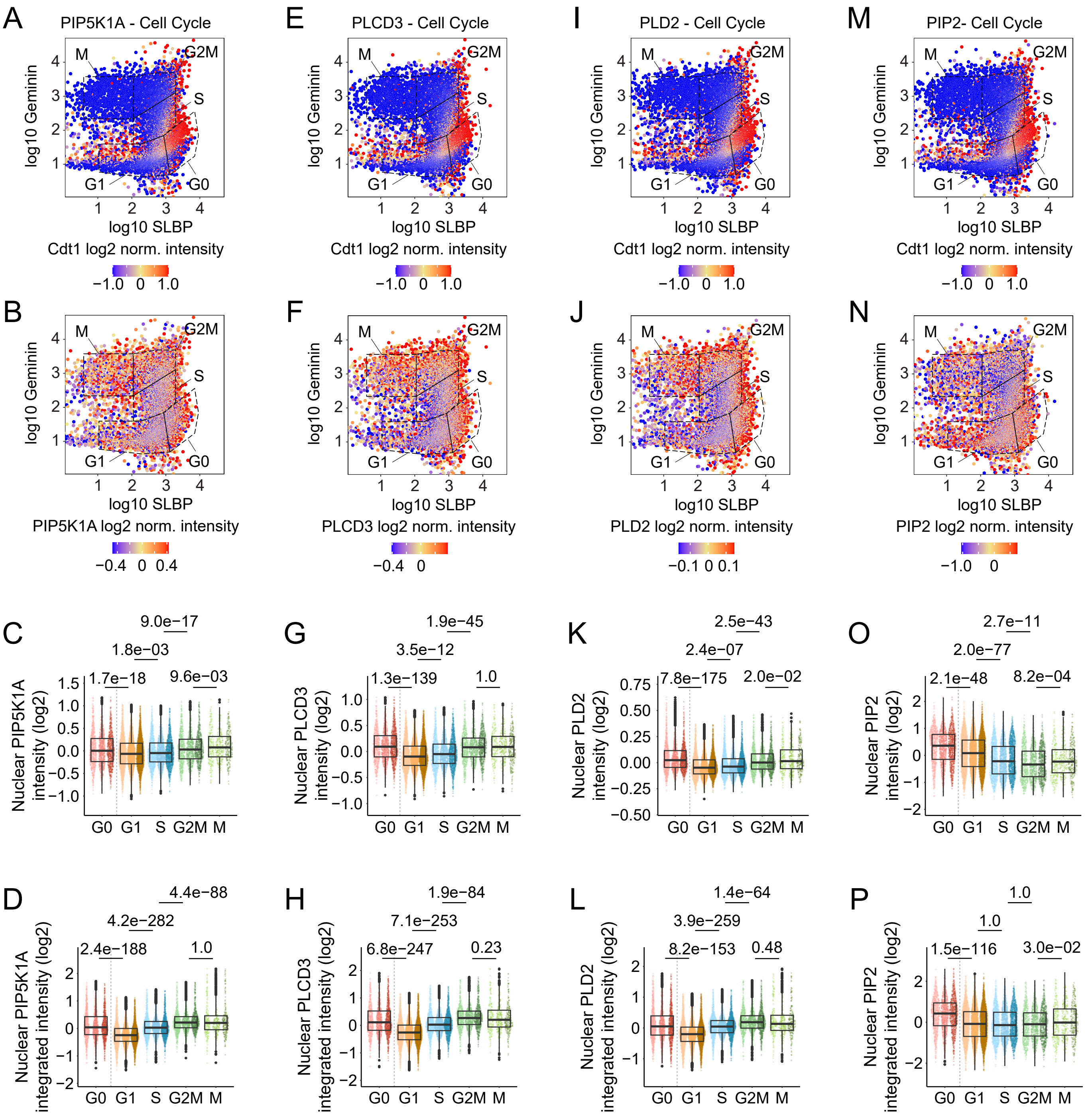

A
PIP5K1A - Cell Cycle
E
PLCD3 - Cell Cycle
I
PLD2 - Cell Cycle
M
PIP2- Cell Cycle
log10 Geminin
log10 SLBP
M
G2M
S
G1
G0
Cdt1 log2 norm. intensity
B
F
J
N
PIP5K1A log2 norm. intensity
PLCD3 log2 norm. intensity
PLD2 log2 norm. intensity
PIP2 log2 norm. intensity
C
G
K
O
Nuclear PIP5K1A intensity (log2)
Nuclear PLCD3 intensity (log2)
Nuclear PLD2 intensity (log2)
Nuclear PIP2 intensity (log2)
9.0e−17
1.8e−03
1.7e−18
9.6e−03
1.9e−45
3.5e−12
1.3e−139
1.0
2.5e−43
2.4e−07
7.8e−175
2.0e−02
2.7e−11
2.0e−77
2.1e−48
8.2e−04
D
H
L
P
Nuclear PIP5K1A integrated intensity (log2)
Nuclear PLCD3 integrated intensity (log2)
Nuclear PLD2 integrated intensity (log2)
Nuclear PIP2 integrated intensity (log2)
4.4e−88
4.2e−282
2.4e−188
1.0
1.9e−84
7.1e−253
6.8e−247
0.23
1.4e−64
3.9e−259
8.2e−153
0.48
1.0
1.0
1.5e−116
3.0e−02
G0 G1 S G2M M

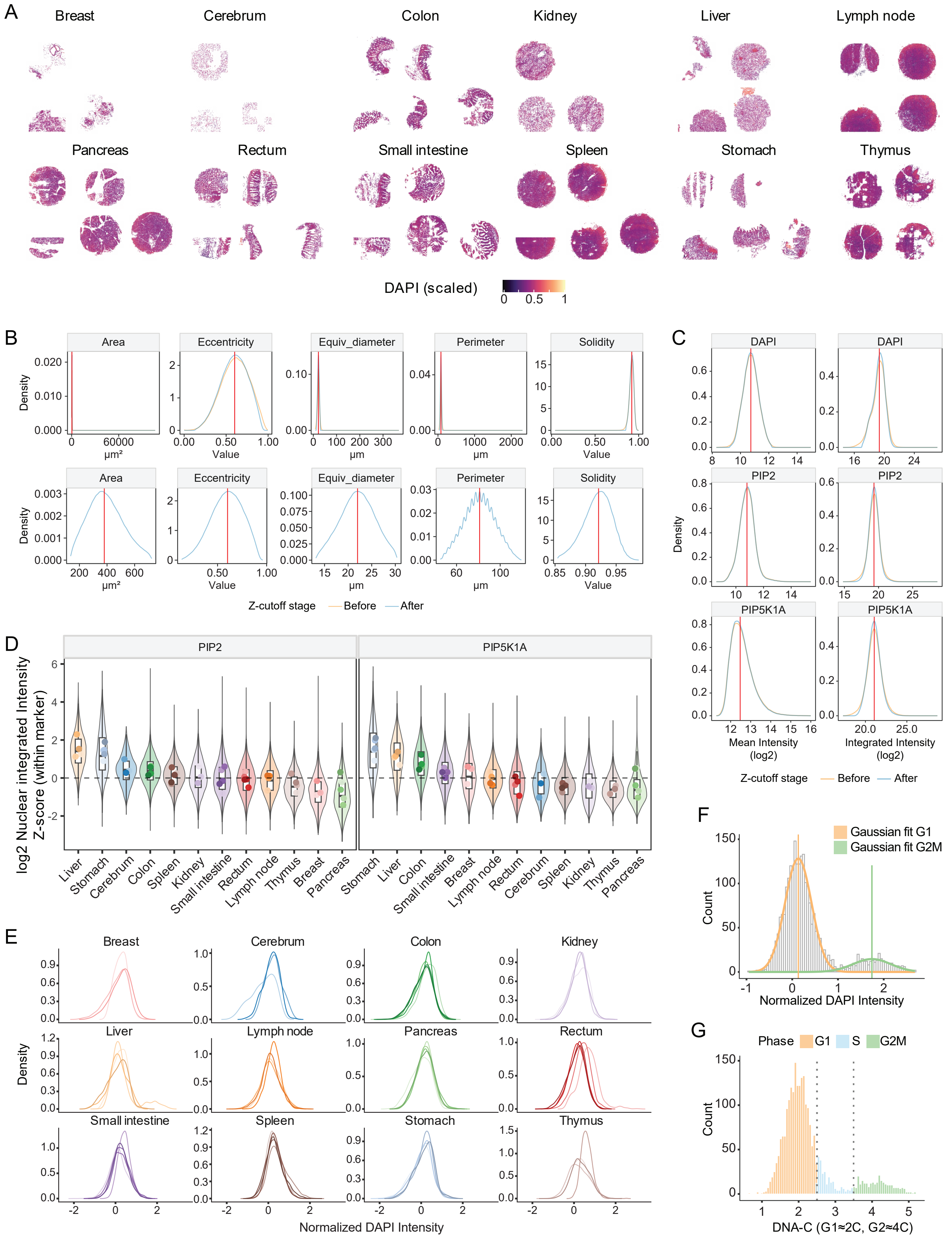
A
Breast
Cerebrum
Colon
Kidney
Liver
Lymph node
Pancreas
Rectum
Small intestine
Spleen
Stomach
Thymus
DAPI (scaled)
0 0.5 1
B
Area
Eccentricity
Equiv_diameter
Perimeter
Solidity
Density
μm²
Value
μm
Z-cutoff stage
Before
After
C
DAPI
PIP2
PIP5K1A
Mean Intensity (log2)
Integrated Intensity (log2)
D
PIP2
PIP5K1A
log2 Nuclear integrated Intensity Z-score (within marker)
Liver
Stomach
Cerebrum
Colon
Spleen
Kidney
Small intestine
Rectum
Lymph node
Thymus
Breast
Pancreas
E
Normalized DAPI Intensity
F
Gaussian fit G1
Gaussian fit G2M
Count
Normalized DAPI Intensity
G
Phase
G1
S
G2M
DNA-C (G1≈2C, G2≈4C)

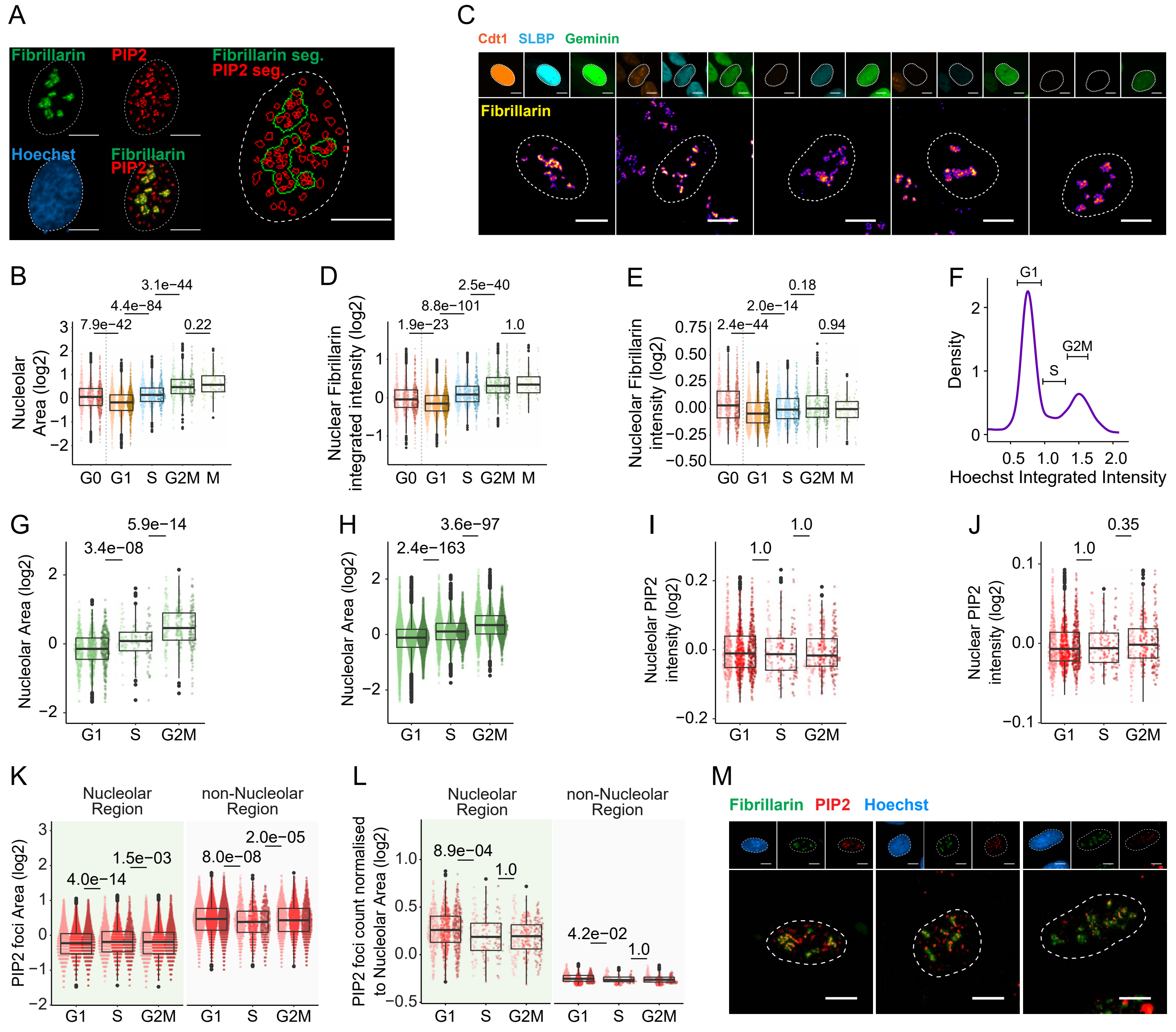
A
Fibrillarin
PIP2
Fibrillarin seg.
PIP2 seg.
Hoechst
Fibrillarin
PIP2
C
Cdt1 SLBP Geminin
Fibrillarin
B
Nucleolar Area (log2)
7.9e−42
4.4e−84
3.1e−44
0.22
G0 G1 S G2M M
D
Nuclear Fibrillarin integrated intensity (log2)
1.9e−23
8.8e−101
2.5e−40
1.0
G0 G1 S G2M M
E
Nucleolar Fibrillarin intensity (log2)
2.4e−44
2.0e−14
0.18
0.94
G0 G1 S G2M M
F
Density
G1
S
G2M
Hoechst Integrated Intensity
G
Nucleolar Area (log2)
3.4e−08
5.9e−14
G1 S G2M
H
Nuclear Area (log2)
2.4e−163
3.6e−97
G1 S G2M
I
Nucleolar PIP2 intensity (log2)
1.0
1.0
G1 S G2M
J
Nuclear PIP2 intensity (log2)
1.0
0.35
G1 S G2M
K
Nucleolar Region
non-Nucleolar Region
PIP2 foci Area (log2)
4.0e−14
1.5e−03
8.0e−08
2.0e−05
G1 S G2M G1 S G2M
L
Nucleolar Region
non-Nucleolar Region
PIP2 foci count normalised to Nucleolar Area (log2)
8.9e−04
1.0
4.2e−02
1.0
G1 S G2M G1 S G2M
M
Fibrillarin PIP2 Hoechst

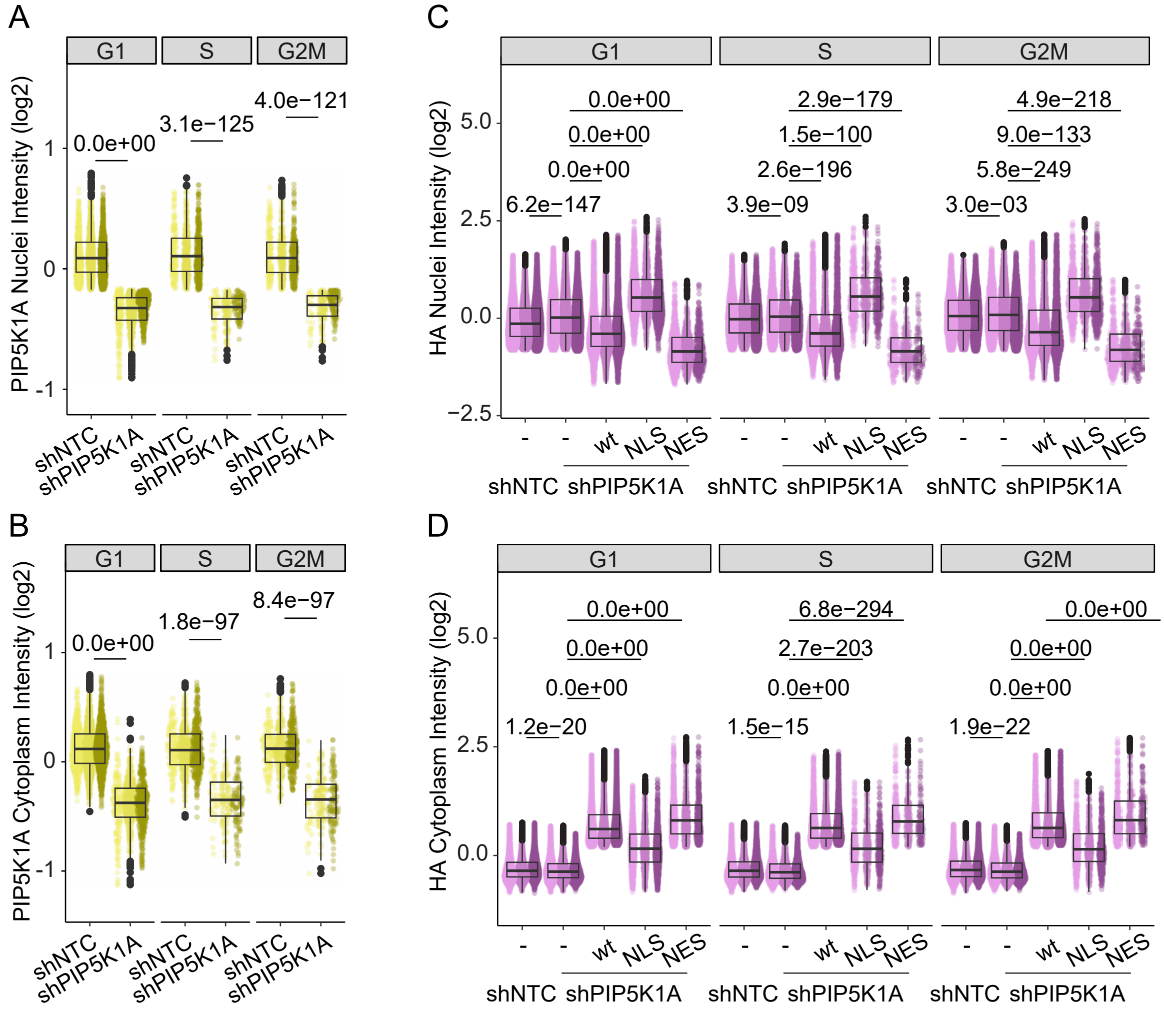
A
G1
S
G2M
PIP5K1A Nuclei Intensity (log2)
1
0
-1
0.0e+00
3.1e−125
4.0e−121
shNTC
shPIP5K1A
shNTC
shPIP5K1A
shNTC
shPIP5K1A
B
G1
S
G2M
PIP5K1A Cytoplasm Intensity (log2)
1
0
-1
0.0e+00
1.8e−97
8.4e−97
shNTC
shPIP5K1A
shNTC
shPIP5K1A
shNTC
shPIP5K1A
C
G1
S
G2M
HA Nuclei Intensity (log2)
5.0
2.5
0.0
−2.5
0.0e+00
0.0e+00
0.0e+00
6.2e−147
2.9e−179
1.5e−100
2.6e−196
3.9e−09
4.9e−218
9.0e−133
5.8e−249
3.0e−03
- - wt NLS NES
shNTC shPIP5K1A
D
G1
S
G2M
HA Cytoplasm Intensity (log2)
5.0
2.5
0.0
0.0e+00
0.0e+00
0.0e+00
1.2e−20
6.8e−294
2.7e−203
0.0e+00
1.5e−15
0.0e+00
0.0e+00
0.0e+00
1.9e−22
- - wt NLS NES
shNTC shPIP5K1A

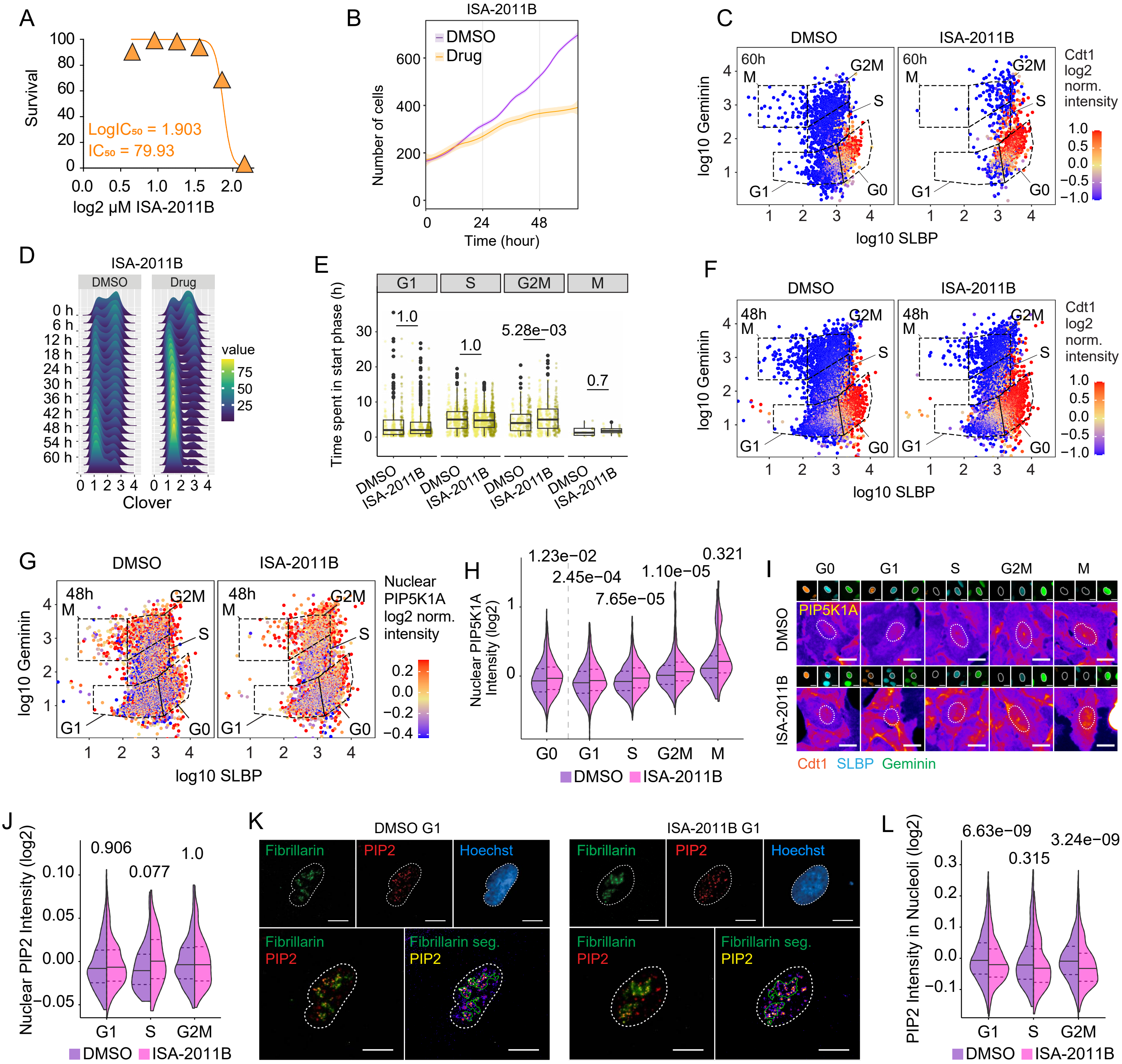
A
Survival
LogIC50 = 1.903
IC50 = 79.93
log2 μM ISA-2011B
B
ISA-2011B
DMSO
Drug
Number of cells
Time (hour)
C
DMSO
ISA-2011B
60h
M
G2M
S
G1
G0
log10 Geminin
log10 SLBP
Cdt1 log2 norm. intensity
D
ISA-2011B
DMSO
Drug
value
Clover
E
G1
S
G2M
M
Time spent in start phase (h)
1.0
1.0
5.28e−03
0.7
DMSO
ISA-2011B
F
DMSO
ISA-2011B
48h
Cdt1 log2 norm. intensity
log10 Geminin
log10 SLBP
G
DMSO
ISA-2011B
48h
Nuclear PIP5K1A log2 norm. intensity
log10 Geminin
log10 SLBP
H
1.23e−02
2.45e−04
7.65e−05
1.10e−05
0.321
Nuclear PIP5K1A Intensity (log2)
G0
G1
S
G2M
M
DMSO
ISA-2011B
I
G0
G1
S
G2M
M
DMSO
ISA-2011B
PIP5K1A
Cdt1
SLBP
Geminin
J
0.906
0.077
1.0
Nuclear PIP2 Intensity (log2)
G1
S
G2M
DMSO
ISA-2011B
K
DMSO G1
ISA-2011B G1
Fibrillarin
PIP2
Hoechst
Fibrillarin seg.
L
6.63e−09
0.315
3.24e−09
PIP2 Intensity in Nucleoli (log2)
G1
S
G2M
DMSO
ISA-2011B

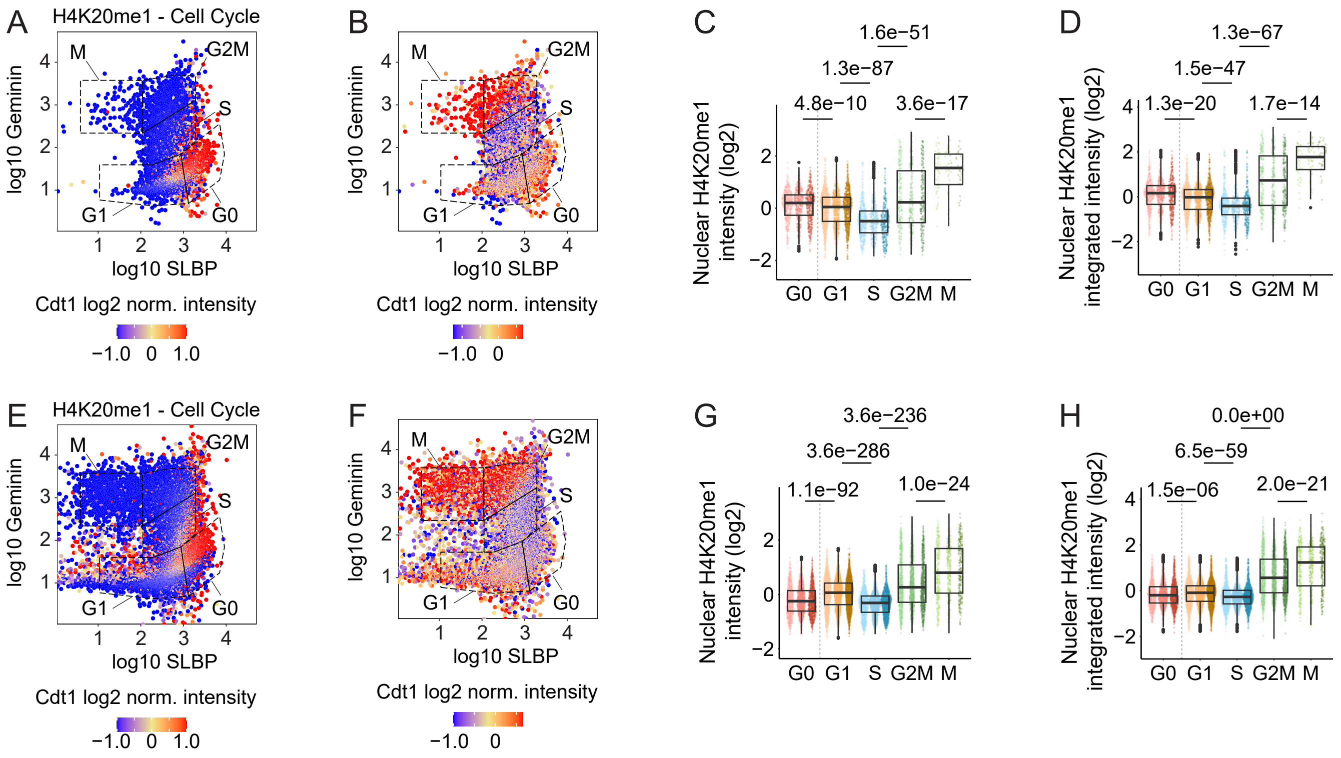
A
H4K20me1 - Cell Cycle
M
G2M
S
G1
G0
log10 Geminin
log10 SLBP
Cdt1 log2 norm. intensity
−1.0 0 1.0
B
M
G2M
S
G1
G0
log10 Geminin
log10 SLBP
Cdt1 log2 norm. intensity
−1.0 0
C
1.6e−51
1.3e−87
4.8e−10
3.6e−17
Nuclear H4K20me1 intensity (log2)
G0 G1 S G2M M
D
1.3e−67
1.5e−47
1.3e−20
1.7e−14
Nuclear H4K20me1 integrated intensity (log2)
G0 G1 S G2M M
E
H4K20me1 - Cell Cycle
M
G2M
S
G1
G0
log10 Geminin
log10 SLBP
Cdt1 log2 norm. intensity
−1.0 0 1.0
F
M
G2M
S
G1
G0
log10 Geminin
log10 SLBP
Cdt1 log2 norm. intensity
−1.0 0
G
3.6e−236
3.6e−286
1.1e−92
1.0e−24
Nuclear H4K20me1 intensity (log2)
G0 G1 S G2M M
H
0.0e+00
6.5e−59
1.5e−06
2.0e−21
Nuclear H4K20me1 integrated intensity (log2)
G0 G1 S G2M M

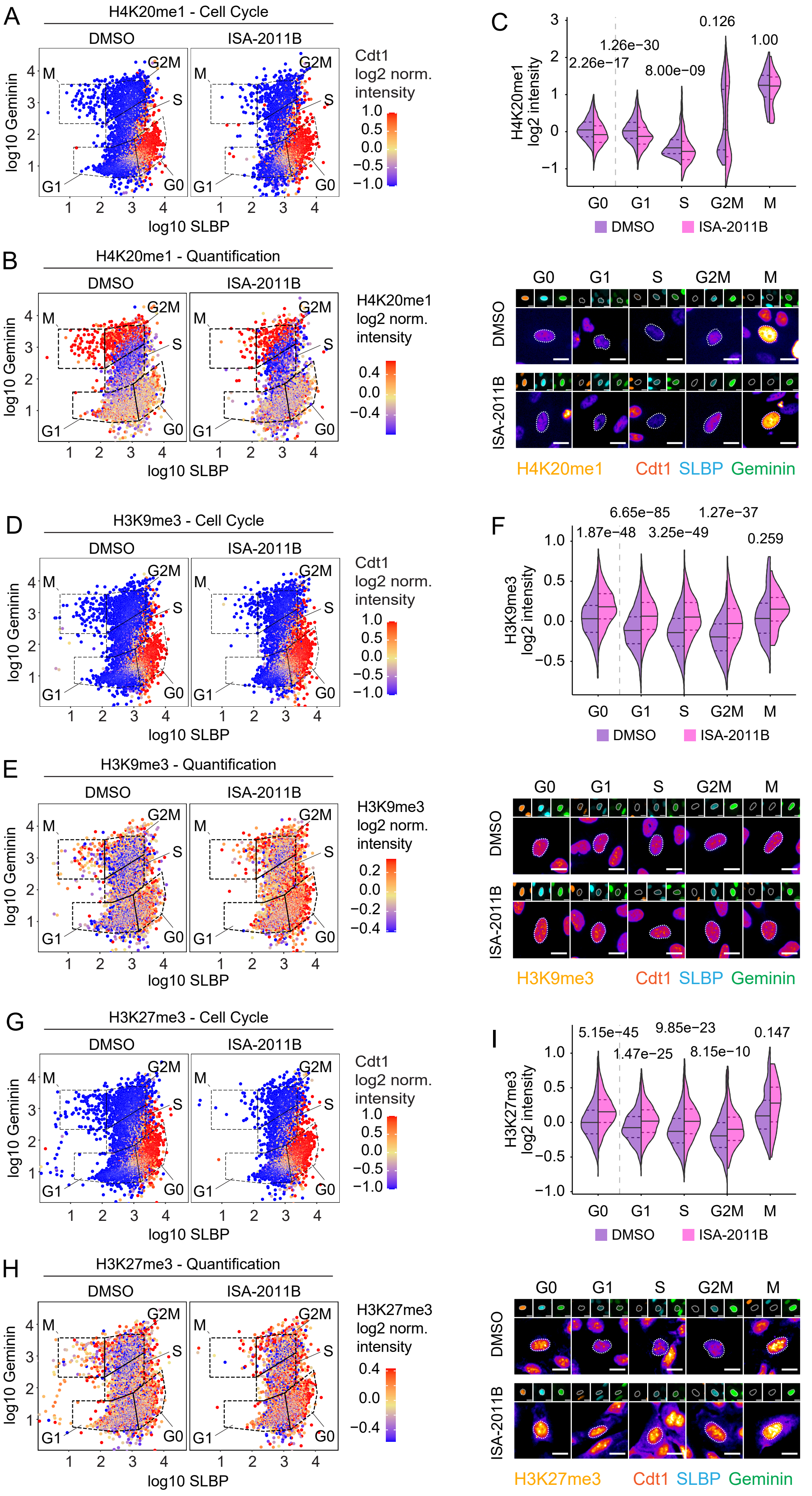
A
H4K20me1 - Cell Cycle
DMSO
ISA-2011B
Cdt1 log2 norm. intensity
log10 Geminin
log10 SLBP
M
G2M
S
G1
G0
B
H4K20me1 - Quantification
H4K20me1 log2 norm. intensity
C
H4K20me1 log2 intensity
2.26e−17
1.26e−30
8.00e−09
0.126
1.00
G0
G1
S
G2M
M
DMSO
ISA-2011B
H4K20me1
Cdt1
SLBP
Geminin
D
H3K9me3 - Cell Cycle
E
H3K9me3 - Quantification
H3K9me3 log2 norm. intensity
F
H3K9me3 log2 intensity
1.87e−48
6.65e−85
3.25e−49
1.27e−37
0.259
H3K9me3
G
H3K27me3 - Cell Cycle
H
H3K27me3 - Quantification
H3K27me3 log2 norm. intensity
I
H3K27me3 log2 intensity
5.15e−45
1.47e−25
9.85e−23
8.15e−10
0.147
H3K27me3

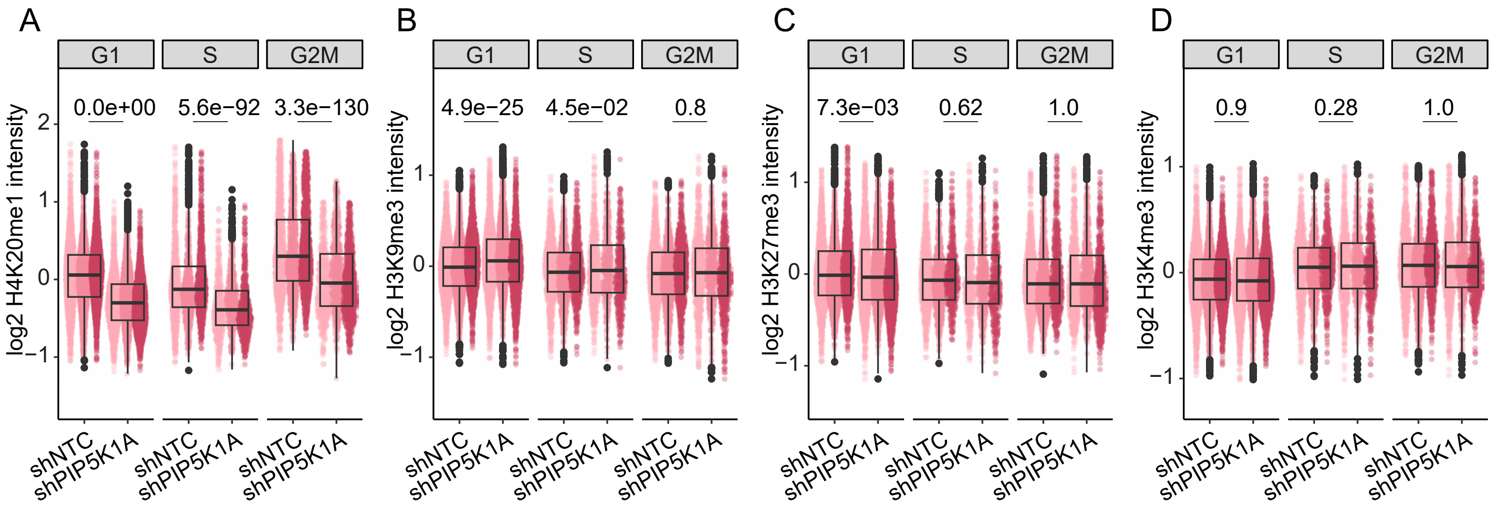
A
G1
S
G2M
0.0e+00
5.6e−92
3.3e−130
log2 H4K20me1 intensity
2
1
0
−1
shNTC
shPIP5K1A
B
4.9e−25
4.5e−02
0.8
log2 H3K9me3 intensity
C
7.3e−03
0.62
1.0
log2 H3K27me3 intensity
D
0.9
0.28
1.0
log2 H3K4me3 intensity
